# Dynamic signal processing by ribozyme-mediated RNA circuits to control gene expression

**DOI:** 10.1093/nar/gkv287

**Published:** 2015-04-27

**Authors:** Shensi Shen, Guillermo Rodrigo, Satya Prakash, Eszter Majer, Thomas E. Landrain, Boris Kirov, José-Antonio Daròs, Alfonso Jaramillo

**Affiliations:** 1 Institute of Systems and Synthetic Biology, Université d’Évry-Val-d'Essonne, CNRS, F-91000 Évry, France; 2 School of Life Sciences, University of Warwick, Coventry CV4 7AL, UK; 3 Instituto de Biología Molecular y Celular de Plantas, CSIC – Universidad Politécnica de Valencia, 46022 Valencia, Spain

## Abstract

Organisms have different circuitries that allow converting signal molecule levels to changes in gene expression. An important challenge in synthetic biology involves the *de novo* design of RNA modules enabling dynamic signal processing in live cells. This requires a scalable methodology for sensing, transmission, and actuation, which could be assembled into larger signaling networks. Here, we present a biochemical strategy to design RNA-mediated signal transduction cascades able to sense small molecules and small RNAs. We design switchable functional RNA domains by using strand-displacement techniques. We experimentally characterize the molecular mechanism underlying our synthetic RNA signaling cascades, show the ability to regulate gene expression with transduced RNA signals, and describe the signal processing response of our systems to periodic forcing in single live cells. The engineered systems integrate RNA–RNA interaction with available ribozyme and aptamer elements, providing new ways to engineer arbitrary complex gene circuits.

## INTRODUCTION

Natural signal transduction systems allow organisms to adapt to fluctuating environments, often by exploiting subcellular localization, molecular cascades and protein allostericity (1,2). A major challenge in synthetic biology involves the engineering of novel signaling systems that sense, process and transmit information. Most engineering efforts have relied on the translational fusion of known protein domains with specific interaction or catalytic functionalities (2). However, this approach is limited by the availability of known natural interaction domains that are specific enough to avoid cross-talk with other molecules in the cellular context. Alternatively, the use of RNA as programmable molecules would allow engineering an unlimited number of interaction partners (3,4). This way, we propose to engineer synthetic signal transduction systems relying on RNA by using a transcriptional fusion strategy, exploiting sequence fragments with definite interaction and catalytic properties. In protein-based signaling, localized folding domains facilitate the engineering (or re-engineering) of multiple functions (5,6). Similarly, there are well-known RNA folding structures that are stable and capable to interact specifically with signaling molecules (aptamers) or to catalyze reactions (ribozymes) (4). In addition, the use of computational tools allows the prediction of conformational changes in many cases, opening the door to the engineering of signal transduction systems based on RNA (7). As a proof of concept, we here develop a system (to control gene expression with a molecular signal) that consists in the fusion of an aptazyme, acting as a molecular sensing element, with a riboregulator, acting as a signal mediator. To simplify the terminology, in the following we refer to this multifunctional RNA molecule as regazyme.

In this direction, pioneering work in synthetic biology inserted known aptamer domains into 5′ untranslated regions (UTRs) of messenger RNAs (mRNAs) to sense small molecules (10), and also exploited riboregulation in combination with small-molecule-responsive promoters to control gene networks and metabolic pathways (8,9). More recently, important steps towards RNA-based sensing have been carried out by engineering aptazymes in the 5′ or 3′ UTRs to sense both small molecules (11,12) and small RNAs (sRNAs) (13). Moreover, previous work has combined aptamers with riboregulators to create novel sensing devices (13–15). Those works exploit the programmability of RNA function through strand-displacement reactions and induced conformational changes. Here, our strategy allows engineering a one-to-two-component signal transduction system, where emerging RNA function is achieved by incorporating self-cleavage ability into a *trans*-acting riboregulator. This corresponds to a four-molecule system, where the first one is the signal molecule, either a small molecule or sRNA, and the last one is a *cis*-regulated mRNA as system's readout. The other two molecules (two components) correspond to the sensor and mediator, which can be switched ON/OFF in presence/absence of the signal molecule, respectively.

The devised system shares properties with natural signaling systems (1). On the one hand, it is a one-component system from the input viewpoint. Thus, it has the advantage of subcellular localization independence. On the other hand, it is a two-component system from the output perspective (the sensor and mediator are different molecules after cleavage). Thanks to the modularity offered by the independence of the sensor and mediator domains, we could have a palette of domains with alternative functionalities. Compared to endogenous sensors (e.g. receptors), our sensor is not limited to the cell membrane, meanwhile the mediator (i.e., riboregulator) works like a phosphorylated transcription factor but at post-transcriptional level (interacting with a 5′ UTR rather than with a promoter). Our approach to engineer a signal transduction system combines the fusion of functional RNA elements together with the computational prediction of each conformational state. This is also possible with a protein-based system (16,17), although it could become much harder, requiring experimental screening towards appropriate transduction properties (5).

In this work, we show that RNA structure is predictable enough to allow a computational design strategy. In general, the tremendous size of the system's sequence space prevents the *de novo* design without automation. We have previously demonstrated that an automated design methodology is able to generate *de novo* riboregulation in live cells (18). Therefore, we here propose to generalize such methodology to design RNA-mediated signal transduction systems. For that, we assume that any interaction between two RNAs is triggered by a seed (or toehold) sequence (18). In the case of a regazyme, the signal molecule induces a catalytic process that releases a riboregulator, which in turn induces a conformational change in the 5′ UTR that initiates interaction with the 16S ribosomal unit (18,19) in *Escherichia coli*. This way, we enforce a hierarchical mode of action consisting in switching ON each functional module, which is initially OFF.

In the following, we will provide a detailed description of the computational methodology to design a hierarchical system with functional RNA modules that couples molecular signals in the cell (either from the environment or from upstream biological systems) with gene expression. We will first describe the development of a methodology for nucleotide sequence design, and then we will present a mechanistic characterization to assess the self-cleavage activity of the regazyme. Subsequently, we will show results assessing signal transduction with time-dependent induction in bacterial cells, allowing the characterization of the dynamic regulatory properties at both population and single cell levels.

## MATERIALS AND METHODS

### Sequence design

We developed a Monte Carlo Simulated Annealing (20) optimization algorithm to design the transducer modules of regazymes provided the sequences of given aptazymes (or sRNA-induced ribozymes) and riboregulators (Supplementary Figure S6). For that, we constructed a basic energy model that involved three variables (to be minimized): the energy of activation corresponding to the catalytic activity of the aptazyme, the degree of accessibility of the riboregulator seed before cleavage, and the degree of obstruction of the seed after cleavage. The exposure or obstruction of the riboregulator seed is governed by secondary structure, but the aptazyme involves tertiary contacts. We here simplified the problem by only considering the secondary structure of the aptamer to calculate the energy of activation for cleavage. Rounds of random mutations (replacements, additions or deletions) were applied and selected with the energy-based objective function. We used the Vienna RNA package (21) for energy and structure calculation (see further details in Supplementary Materials and Methods). The sequences of the engineered regazymes in this work are shown in Supplementary Tables S1–S3.

### Plasmid construction

The different RNA devices were chemically synthesized and cloned in plasmid pSynth (pUC replication origin, ampicillin resistance) and then subcloned into plasmids pSTC1 or pSTC2. These two plasmids contain a pSC101m replication origin (a mutated pSC101 ori giving a high copy number) and a kanamycin resistance marker (Supplementary Figures S1 and S2). The pSTC2 vector is based on our previously reported vector pSTC1 (18) by removing the mRFP coding sequence and tagging the carboxyl terminus of the superfolder GFP (sfGFP) (22) with the *ssr*A degradation tag (23). Dysfunctional regazymes were constructed by PCR-based site-directed mutagenesis (see Supplementary Materials and Methods). Strains and plasmids used in this study are listed in Supplementary Table S6.

### Intracellular catalytic activity

Processing extent of regazyme at different time points (0, 2, 4, 8, 16 and 32 min) was analyzed by northern blot hybridization using a complementary [^32^P]-labeled RNA probe after separating the different RNA samples by denaturing polyacrylamide gel electrophoresis (PAGE). RNA preparations were mixed with formamide loading buffer for denaturation, followed by PAGE separation in 5% polyacrylamide gels including 8 M urea and TBE buffer. Gels were stained with ethidium bromide. Membranes were hybridized overnight, imaged by autoradiography, and then hybridization signals quantified by phosphorimetry (Fujifilm FLA-5100). See more details in Supplementary Materials and Methods.

### Fluorescence quantification

Cells were grown overnight in LB medium, and were refreshed in culture tubes with LB medium in order to reach stationary phase. Cells were then diluted 1:200 in 200 μl of M9 minimal medium in each well of the plate (Custom Corning Costar). The plate was incubated in an Infinite F500 multi-well fluorometer (TECAN) at 37°C with shaking. It was assayed with an automatic repeating protocol of absorbance measurements (600 nm absorbance filter) and fluorescence measurements (480/20 nm excitation filter – 530/25 nm emission filter for sfGFP) every 15 min. All samples were present in triplicate on the plate (see further details in Supplementary Materials and Methods).

### Single cell microfluidics analysis

The design of our microfluidics device (Supplementary Figure S16) (24), which was performed in AUTOCAD (AUTODESK), was adapted from the previous one reported by Hasty *et al*. (25). All images were acquired using Zeiss Axio Observer Z1 microscopy (Zeiss). The microscope resolution was 0.24 μm with Optovariation 1.6×, resulting total magnification 1600× for both bright field and fluorescent images. Images were analyzed with MATLAB (MathWorks). Cells were tracked by defining a cell-to-cell distance matrix and the cell lineages were reconstructed. Finally, the fluorescence level of each cell in each fluorescence frame was extracted (see further details in Supplementary Materials and Methods).

## RESULTS

### Computational design of RNA-mediated signal transduction systems to control gene expression

Our modular strategy consists in designing switchable functional RNA domains, which is implemented by exploiting strand-displacement principles together with the engineering of allosteric conformational states. This way, we can design chains of several domains that are activated in cascade. Without loss of generality, we considered a system composed of two transcriptional units: regazyme and mRNA of a reporter gene (e.g. a gene coding for a green fluorescent protein − GFP), but our methodology could be generalized to an arbitrary number of transcriptional units containing switchable functional elements. To engineer such a synthetic RNA system implementing the transduction of molecular signals into changes in gene expression, we took advantage of a standard physicochemical model (based on Watson-Crick and wobble pairing) predicting RNA secondary structure and free energy (26) to be used in an optimization algorithm to select for the hierarchical activation of functional RNA modules in the cascade (7).

**Figure 1.**
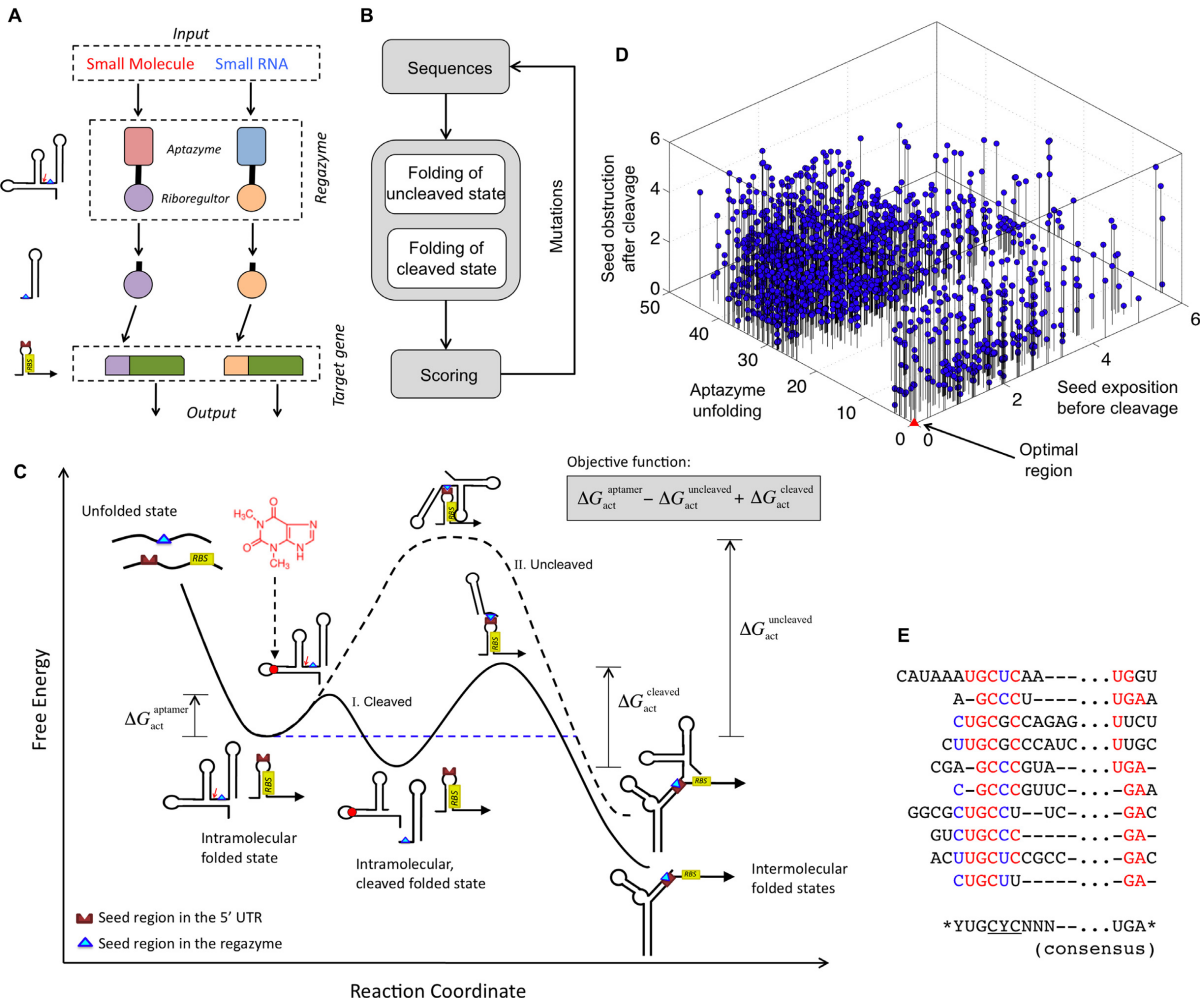
Computational design of the regazyme signaling pathway. (**A**) Scheme of the modular system, where a signal molecule (either a small molecule or a sRNA) induces a catalytic reaction that releases a riboregulator able to activate gene expression. Each signaling pathway is viewed as a wire carrying information. (**B**) Scheme of the optimization loop, where a regazyme sequence is iteratively mutated and evaluated according to an objective function. (**C**) Energy landscape of the signaling pathway showing the different conformational states (intra- and intermolecular), together with the three free energy terms of the objective function, in terms of a reaction coordinate. Solid line illustrates the trajectory corresponding to the ligand-induced cleavage of the regazyme and subsequent binding of the riboregulator to the mRNA. Dashed line corresponds to the trajectory where the uncleaved regazyme binds to the mRNA. (**D**) Computation of the free energy terms of the objective function for 1,000 random sequences to evaluate their distribution. (**E**) Alignment of different optimized sequences with aptazyme theoHHAz and riboregulator RAJ12. Highly conserved nucleotides are highlighted in red or blue, and the consensus sequence is shown.

In particular, our system corresponds to a cascade of three modules: sensor (aptazyme designed to specifically respond to a given ligand), mediator (riboregulator designed to specifically activate a *cis*-repressed ribosome-binding site − RBS), and actuator (mRNA with *cis*-repressed RBS) (Figure 1a). To create the regazyme, we fused an aptazyme element, acting as a molecular sensing device, with a riboregulator, acting as a signal mediator, into the same transcriptional unit. This fusion is performed with flanking sequences that form a stem and function as a transducer module, in the same way as when designing allosteric aptamers (27). The sensor domain (aptazyme) is initially in a state OFF (catalytically inactive) and is switched to ON (catalytically active) only when it acquires its functional conformation, which is induced by the signal molecule. The mediator domain (riboregulator) will be in a state ON when its seed sequence is exposed to the solvent. We designed the transducer module to ensure that the sensor and mediator were ON/OFF in presence/absence of the signal molecule. This prevents any premature release of the mediator or any direct activation of gene expression by the regazyme. Afterward, the input signal produces a stabilization of an alternative conformation where the aptazyme is active. Once the aptazyme is active, it will self-cleave releasing the mediator domain, which is then switched on (i.e. the riboregulator seed sequence becomes exposed). Once the mediator domain is active, it will diffuse towards its target genes (in particular, to interact with 5′ UTRs), similarly to phosphorylated transcription factors in the conventional two-component systems (1). To be noted, the independence between the sensor and mediator domains favors expanding the functional repertoire, which allows them to be exchanged with alternative domains.

We here propose a new methodology to engineer one-to-two-component signal transduction, which combines the advantages of subcellular independence of one-component systems and of modularity of two-component systems. We constructed a combinatorial optimization problem to explore the sequence space of the transducer module (Figure 1b), where a nucleotide-level energy model considering the conformational states (uncleaved and cleaved) of the regazyme was used to evaluate the performance of the generated sequences. For each state, the model accounts for its free energy and its secondary structure. As objectives to be optimized (computed as Hamming distances), the algorithm considers the energy of activation corresponding to the catalytic activity of the aptazyme (which we assume depends on the correct formation of the aptamer in the uncleaved state), and the degree of exposure to the solvent of the riboregulator seed before and after cleavage (28). Figure 1c illustrates the energy landscape associated to the molecular mechanism of the regazyme, reporting the different conformational states and their corresponding free energy levels (see also Supplementary Figure S4). The reaction coordinate was defined here as the number of intermolecular hydrogen bonds, on one side, between the ligand and the aptazyme and, on the other side, between the riboregulator and the 5′ UTR (in terms of base-pairs). In absence of signal molecule, the progression of the reaction is limited by the presence of a high-energy intermediate that prevents the interaction between the regazyme and the 5′ UTR. However, when the signal molecule is at sufficient concentration, a cleavage is produced and then the activation energy for the resulting riboregulatory element is lowered, which speeds up the reaction (29).

As shown by a random sampling of 1000 sequences (Figure 1d), an optimal score (zero, as our score is considered as a penalty) is very unlikely to be obtained arbitrarily. This means that this is a difficult design problem for a manual approach, requiring automated computation for efficient sequence design. Our algorithm designs by optimization the sequences implementing the intended signal transduction according to the objective function. Even though distinct solutions can be equally good computationally (i.e., according to the objective function), experiments could distill differences in performance among them. We observed, for the sampled sequences, that the aptamer (in the uncleaved state) is correctly formed only for a small subset of sequences (Supplementary Figure S5). Moreover, the resulting distribution is apparently bimodal (Sarle's bimodality coefficient BC = 0.630 > 5/9) (30), which may be explained by an all-or-none formation of the functional structure of the aptazyme. Such pre-organized conformations will favor ligand binding and subsequent cleavage, whereas structures requiring considerable rearrangements will be offside due to a given free energy barrier (29). The distribution of score values along the axis representing the seed exposure in the uncleaved state is more homogeneous (BC = 0.467 < 5/9), whereas the distribution in the cleaved state shows substantial heterogeneity (BC = 0.639 > 5/9). This may be explained by an interaction of the seed region with part of the 5′ end after cleavage (see, for example, Supplementary Figure S8).

### Modularity in the design of regazymes

**Figure 2.**
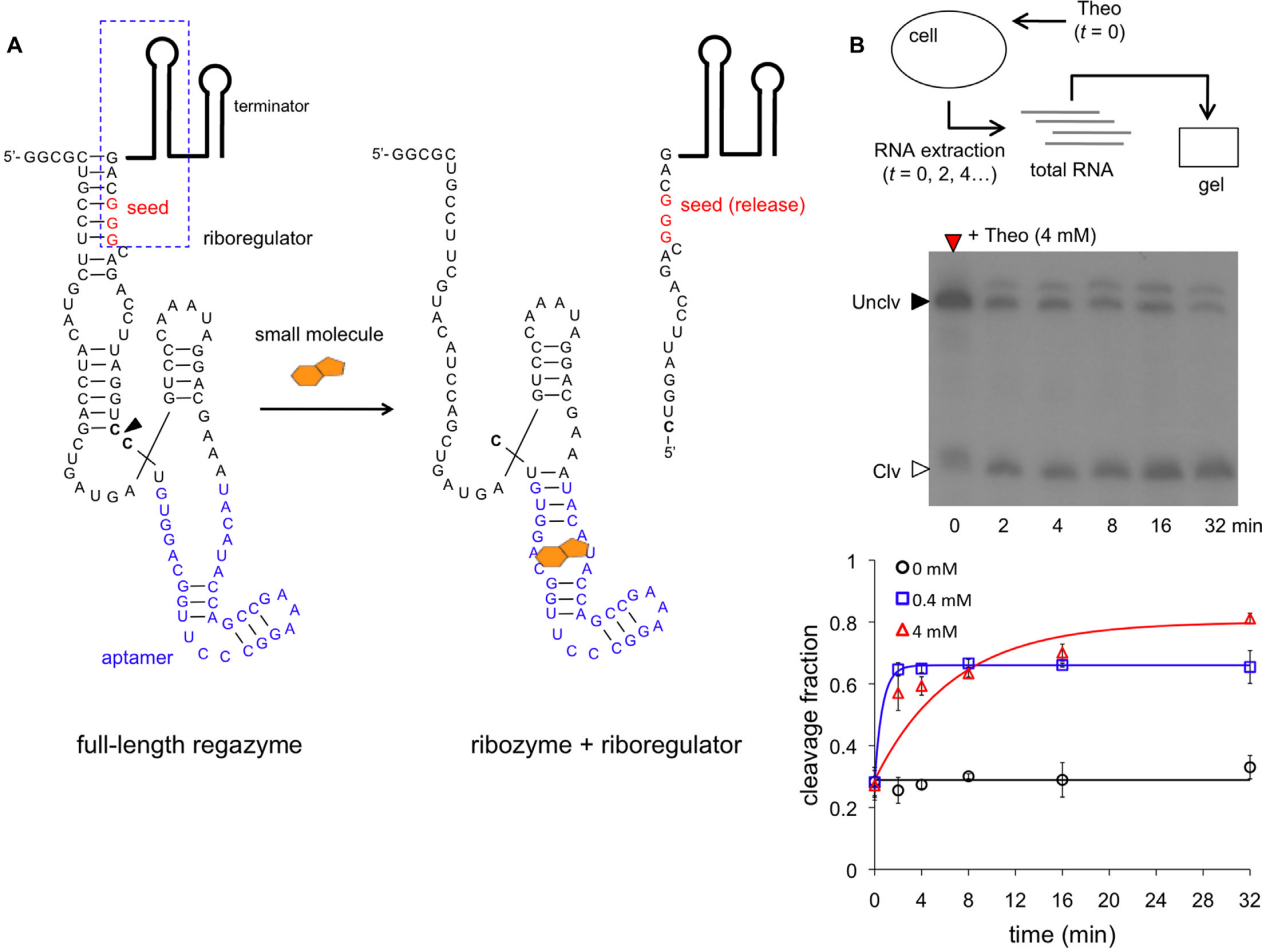
Molecular characterization of small-molecule-sensing regazyme. (**A**) Sequence and structure of the regazyme theoHHAzRAJ12. A small molecule (Theo) binds to the regazyme to reconstitute the active conformation of the ribozyme and then produce the cleavage. An arrow marks the cleavage site, between the transducer module and the ribozyme core. The seed of the riboregulator is paired in the uncleaved state. (**B**) Time-dependent electrophoretic analysis of cellular RNA extracts taken at different time points; gel shown for 4 mM Theo. Quantification of dynamic RNA processing for different concentrations of the signal molecule (Theo). Data fitted with a generalized exponential decay model with production, where the temporal factor is (1 − exp(−*λt*))^*m*^, with *m* ≈ 1. Error bars represent standard deviations over replicates.

**Figure 3.**
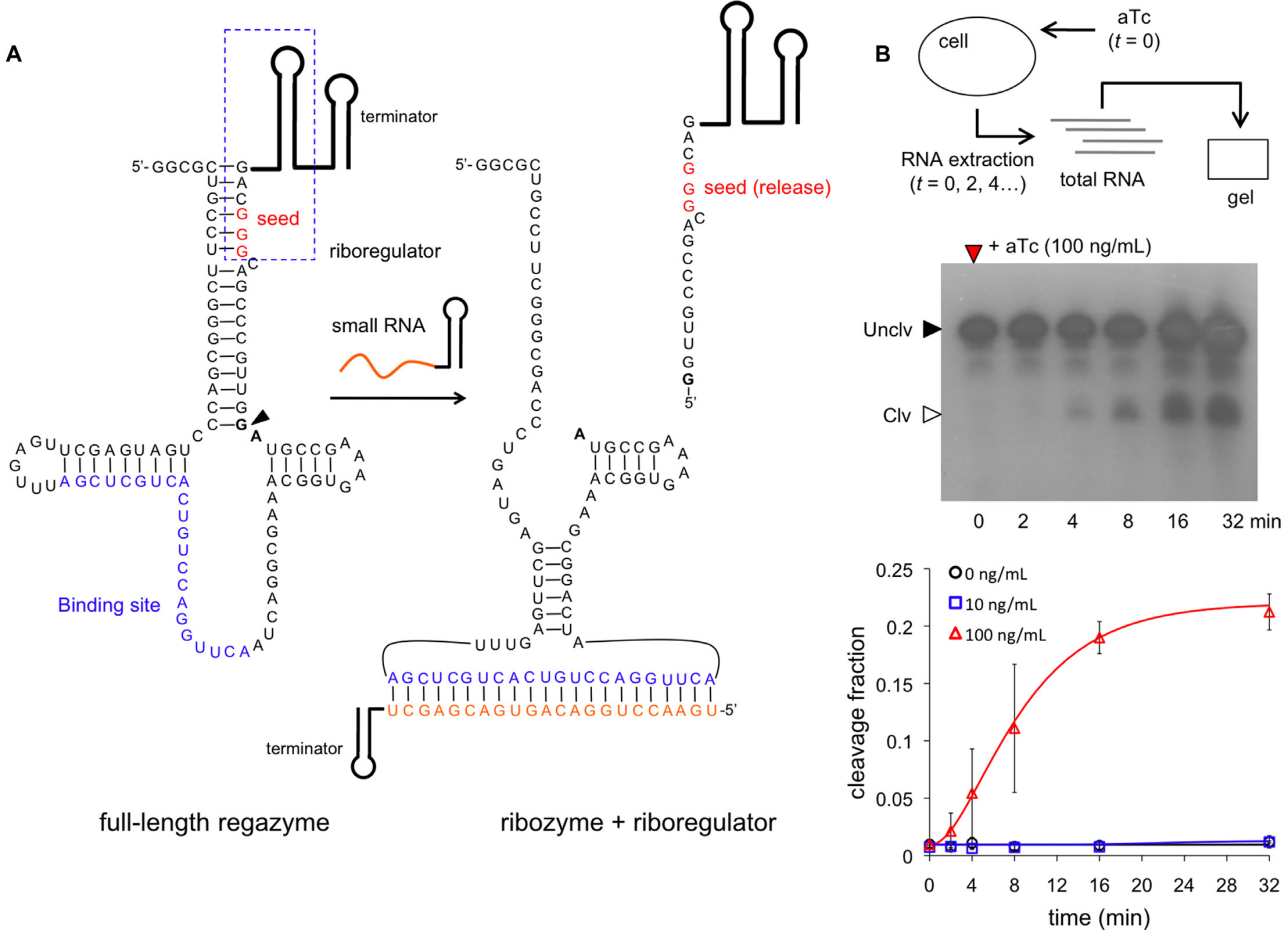
Molecular characterization of sRNA-sensing regazyme. (**B**) Sequence and structure of the regazyme breakHHRzRAJ12. A sRNA binds to the regazyme to reconstitute the active conformation of the ribozyme and then produce the cleavage. An arrow marks the cleavage site, between the transducer module and the ribozyme core. The seed of the riboregulator is paired in the uncleaved state. (**B**) Time-dependent electrophoretic analysis of cellular RNA extracts taken at different time points; gel shown for 100 ng/ml aTc. Quantification of dynamic RNA processing for different concentrations of the signal molecule (aTc). Data fitted with a generalized exponential decay model with production, where the temporal factor is (1 − exp(-*λt*))^*m*^, with *m* ≈ 2. Error bars represent standard deviations over replicates.

In this work, we considered three possible sensor domains, two sensing a small molecule (theophylline − Theo − and thiamine pyrophosphate − TPP, Figure 2 and Supplementary Figure S7), and another sensing a specific sRNA (Break1, Figure 3). This sRNA is induced with anhydrotetracycline (aTc) in our system. More specifically, each sensor is composed of a binding domain (e.g., an aptamer) and a catalytic domain (e.g. a hammerhead ribozyme). Our ligand-induced ribozymes (aptazymes) are theoHHAz and tppHHAz for sensing small molecules (11,31), and breakHHRz for sensing sRNA (32) (Supplementary Figure S3). For the mediator domain, we considered three synthetic riboregulators known to activate the initiation of translation, two engineered in Rodrigo *et al*. (RAJ11 and RAJ12) (18) and one in Isaacs *et al*. (RR12) (19) (Supplementary Figure S8). We then designed the rest of the regazyme sequence according to the specifications required to generate the RNA signaling cascade. Exploiting the modularity of this system, we engineered the following regazymes: theoHHAzRAJ11, theoHHAzRAJ12, theoHHAzRR12, tppHHAzRAJ12 and breakHHAzRAJ12. The regazyme produces a mediator molecule (riboregulator) that is independent of the signal and sensor molecules. In the following, we investigate, on the one hand, how different signal molecules (Theo, TPP and Break1) activate a common mediator (RAJ12), and, on the other hand, how different implementations of the wire (RAJ11, RAJ12 and RR12) transduce the information from a common signal molecule (Theo).

Our computational approach allowed us to investigate the designability (defined as the number of sequences that have the desired biochemical function) of the solution space for a particular couple of aptazyme and riboregulator. We expect, nevertheless, a higher designability when sequences are allowed to vary in length, as it is the case for our algorithm. It is instructive to align multiple solutions to reveal conserved nucleotide positions. Figure 1e shows, for different designs, the consensus sequence for a given choice (aptazyme theoHHAz and riboregulator RAJ12). Sequences (corresponding to the 5′ and 3′ regions of the aptazyme, see Supplementary Figure S6a) were aligned by using the anti-seed consensus sequence (CYC in this case; note that the seed sequence is GGG) as reference. In addition, this modularity would allow a hierarchical design of the regazyme molecule. To create a suitable pipeline, we can exploit computational algorithms to: (i) design the binding domain (aptamer in particular) for a specific signal molecule (33), (ii) design the riboregulator and cognate 5′ UTR (18), and (iii) apply the methodology developed in this work to design the appropriate transducer module. Experimental screenings or directed evolution techniques (34) could also be applied, especially to link the binding and catalytic domains (see ‘Discussion’ section).

### Molecular characterization of RNA-mediated signal transduction

To analyze the mechanism of the signaling pathway, we first carried out a kinetic and dose-dependent study of the catalytic activity. The predicted secondary structure of the small-molecule-sensing regazyme theoHHAzRAJ12 in the uncleaved state (Figure 2a) shows, as designed, that the aptamer is already arranged for Theo sensing, and that the seed region of the riboregulator is blocked by the transducer module. After cleavage at the CC dinucleotide site between the transducer module and the ribozyme core (Figure 2a, marked by an arrow), the seed region is released, which allows the riboregulator to interact downstream with the cognate 5′ UTR of the reporter gene. An analogous seed-based structural mechanism is illustrated for the sRNA-sensing regazyme breakHHRzRAJ12 (Figure 3a). In this case, the binding domain is only partially paired to allow an efficient interaction with the signal sRNA (Break1), and the cleavage is done at the GA dinucleotide site (Figure 3a, marked by an arrow). Indeed, there is a seed-mediated interaction between Break1 and the regazyme, similar to the interaction between the riboregulator and the 5′ UTR. Of note, our regazyme breakHHRzRAJ12 implements for the first time an RNA cascade in live cells (independent of any protein-based machinery).

To monitor the dynamic RNA processing of the system, we performed a gel assay from cellular RNA extracts. Cells expressing regazyme theoHHAzRAJ12 or breakHHRzRAJ12 were induced with different concentrations of Theo or aTc and lysed at several time points. The gel assays in both cases showed fast dynamic RNA processing, reaching steady states in almost 16 min. The observed cleavage rate (fitted with a model of exponential decay with production) is 0.15 min^−1^ for theoHHAzRAJ12 with 4 mM Theo, although the model does not capture finely the experimental trend (Figure 2b and Supplementary Figure S9a). In this case, the band corresponding to the released riboregulator (of 114 nt, accounting for the terminator) migrates faster than expected. This band was not observed without Theo, indicating that indeed it is a product of the cleavage reaction. Moreover, we note that our probe did not detect the 5′ fragment after cleavage, suggesting a fast degradation of this new species. In those conditions, the maximal cleavage fraction is ∼80%, which is >2.5-fold increase with respect to the basal state (∼30%). With 0.4 mM Theo, the observed cleavage rate is 1.5 min^−1^ with a more accurate fitting (note that the discrepancy between the rates at 4 and 0.4 mM is indeed due to the model fitting; at 4 min the fraction cleaved is ∼60% in both cases). According to previous experimental results *in vitro* without RNA production and degradation (11), the observed cleavage rates of theoHHAz in absence and presence (4 mM) of Theo are 1.3 and 3.6 min^−1^, respectively, with a maximal cleavage fraction of 90%. Certainly, the dynamic response *in vivo* faces additional challenges due to the balance between production and degradation. For breakHHRzRAJ12, the observed cleavage rate is 0.17 min^−1^ with 100 ng/ml aTc, but no activity is reported for lower concentrations of this inducer (Figure 3b and Supplementary Figure S9b). Here, the band corresponding to the released riboregulator (of 112 nt, also accounting for the terminator) migrates slower than expected, although it was not observed (as before) without aTc, indicating that indeed it is a product of the cleavage reaction. These anomalous migrations could be due to a difference in expected length (e.g. unpredicted transcription termination, as most of the cleavage occurs co-transcriptionally) or to a residual structure in the released riboregulator even after using 8 M urea in the gel, among other possibilities. In case of breakHHRzRAJ12, previous assays *in vitro* (32) revealed a rate of 0.11 min^−1^ with single-stranded DNA as ligand (3 μM). The maximal cleavage fraction reported here is almost 25% (∼1% for the basal state), which shows a big discrepancy with those previous *in vitro* results (∼75%). One possible explanation is that the expression of the sRNA with 100 ng/ml aTc does not saturate the system, because the regazyme is expressed from a strong constitutive promoter, and also because of a high effective dissociation constant. Of relevance, this regazyme has much lower leakage (1% versus 30%, although maintaining similar fold-changes), which could be important in case of sensitive systems. Moreover, we observed higher heterogeneity in the dynamic response (from cell to cell) for this sRNA-sensing regazyme, which could be a result of a heterogeneous expression of the sRNA or even of a certain heterogeneous sRNA–regazyme interaction. As a result, by predicting RNA structures and quantifying cellular RNA extracts, we have shown the precise signal sensing and subsequent cleavage to release a functional riboregulator.

To further confirm that our devices behave as expected, we performed *in vitro* transcription of systems theoHHAzRAJ12 and breakHHRzRAJ12. The experiments showed similar cleavage fractions (with respect to the *in vivo* assays) after 30 min of reaction (Supplementary Figure S10a). For theoHHAzRAJ12, 70% of the molecules were cleaved *in vitro*, whereas 80% were *in vivo*. For breakHHRzRAJ12, 25% of the molecules were cleaved both *in vitro* and *in vivo*. We also observed that theoHHAzRAJ12 was cleaved in higher extent in absence of ligand. Because *in vitro* we can neglect degradation, the cleavage fraction is expected to increase with time, in presence of ligand and also in absence of it due to the basal activity of the ribozyme. We further performed a time-course assay to study the cleavage of the regazymes. As shown in our experimental results, the fraction of cleaved products of theoHHAzRAJ12 in test tubes was only ∼10% larger when Theo was present (Supplementary Figure S10b). However, when the same RNA was monitored in bacterial cells, the apparent level of induction of cleavage activity by theophylline was >2.5-fold. The difference in cleavage *in vitro* in case of breakHHRzRAJ12 with respect to the presence or not of Break1 (introduced as DNA oligo) was more remarkable (Supplementary Figure S10c). There is certain number of reasons why an RNA might exhibit different behaviors *in vitro* than *in vivo*. For example, *in vitro* the regazyme might exist in thermodynamic equilibrium with its ligand, resulting in a different effective dissociation rate, or differ slightly in length from the strands *in vivo*. In addition, *in vitro* we used T3 polymerase for transcription (without terminators) instead of *Escherichia coli* polymerase and a higher Mg^2+^ concentration than *in vivo*, which might result in differences in folding and cleavage kinetics of the regazyme. In the following, we present the net effect of regazyme cleavage and riboregulator release on GFP expression *in vivo* (both at the population and single cell levels) with and without the ligand, showing a regulatory behavior as designed.

### Regulation of gene expression in live cells with transduced RNA signal

**Figure 4.**
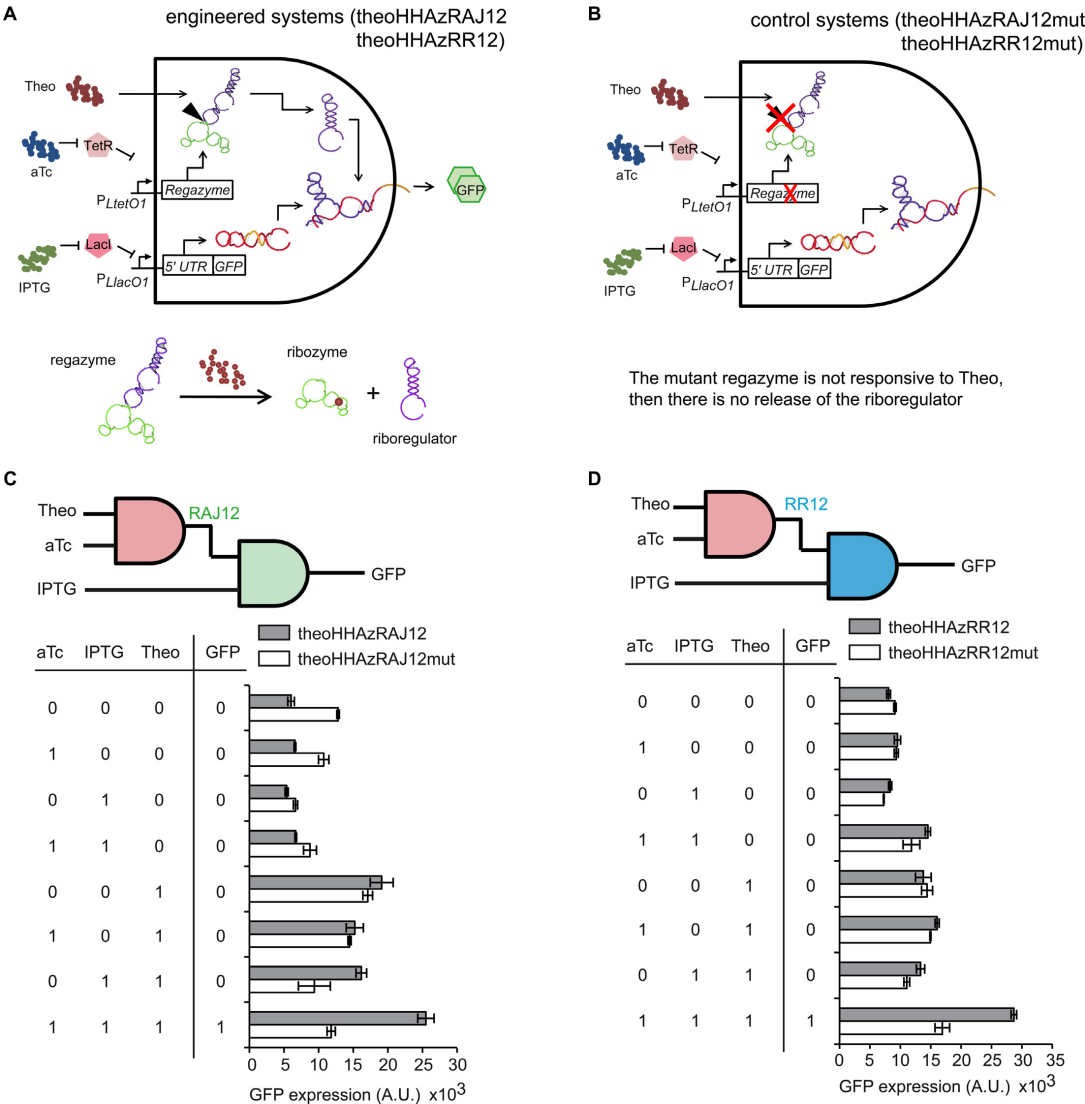
Functional characterization of small-molecule-sensing regazymes. (**A**, **B**) Schemes of the engineered RNA-based circuit to sense a small molecule and its corresponding control. (**C**, **D**) Digital scheme, associated Truth table, and fluorescence results of regazymes theoHHAzRAJ12 and theoHHAzRR12 (gray bars), and of their dysfunctional mutants (white bars) for all possible combinations of inducers. Error bars represent standard deviations over replicates.

To characterize the dynamic range of our engineered systems, we placed the transcriptional units corresponding to the regazyme and mRNA of the GFP reporter gene under the control of tunable promoters (35). These promoters can be induced with isopropyl-β-d-thiogalactopyranoside (IPTG) and aTc in *E. coli* cells expressing constitutively the repressors LacI and TetR. Thus, our systems implement multi-input AND logic circuits (Figure 4a). Moreover, a control system was implemented by using a dysfunctional mutated regazyme (Figure 4b). In the implementation for small-molecule signaling, aTc and IPTG control the expression of the regazyme and the mRNA, and Theo is the signal molecule that induces the cleavage of the regazyme to release the riboregulator. Figure 4c shows the fluorescence results of the system based on regazyme theoHHAzRAJ12 for all possible combinations of inducers (IPTG, aTc and Theo). The observed weak activation of fluorescence in absence of the signal molecule, but in presense of IPTG and aTc, can be explained by the leakage of self-cleavage of the regazyme (see also Supplementary Figure S11). The dysfunctional regazyme, obtained by a two-nucleotide mutation in the aptamer domain that abolishes ligand binding (Supplementary Figure S7a), was shown to significantly decrease GFP expression (Figure 4c). Furthermore, a single-nucleotide mutation in the ribozyme catalytic core (theoHHAzRAJ12AGm, A to G in Supplementary Figure S7a) (46) that inhibits the self-cleavage activity showed decreased GFP expression (Supplementary Figure S11c). An additional inactivating point mutation (theoHHAzRAJ12Cm and theoHHAzRR12Cm, U to G in Supplementary Figure S7a) (11) also revealed decreased GFP expression with respect to the native sequence (Supplementary Figure S11a,b). We also engineered and characterized systems based on riboregulators RR12 (Figure 4d) and RAJ11 (Supplementary Figure S11d), although the riboregulatory activity of theoHHAzRAJ11 with respect to its dysfunctional mutant was more moderate. Furthermore, these three regazymes for Theo-signaling have responsiveness in a dose-dependent manner (Supplementary Figure S14) with an effective dissociation constant of about 1 mM. Another engineered system for TPP signaling (regazyme tppHHAzRAJ12) showed no significant riboregulatory activity (Supplementary Figure S15).

**Figure 5.**
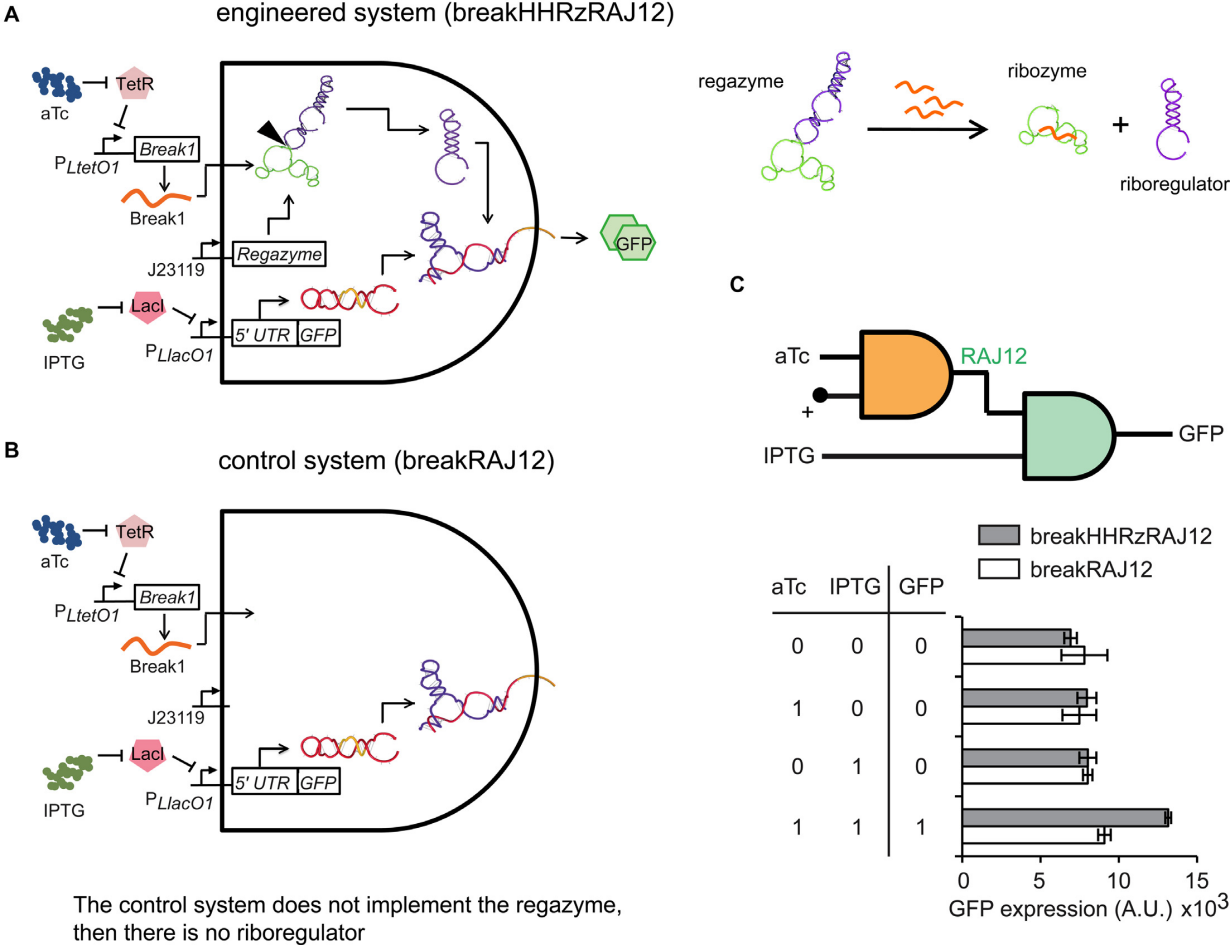
Functional characterization of sRNA-sensing regazyme. (**A**, **B**) Schemes of the engineered RNA-based circuit to sense a sRNA and its corresponding control. (**C**) Digital scheme, associated Truth table, and fluorescence results of regazyme breakHHRzRAJ12 (gray bars), and of its dysfunctional mutant (white bars) for all possible combinations of inducers. Error bars represent standard deviations over replicates.

In the implementation for sRNA signaling, aTc and IPTG control the expression of the sRNA working as signal molecule and the mRNA, whereas the regazyme is expressed from a strong constitutive promoter (Figure 5a). In this case, the cleavage of the regazyme is induced by that sRNA. Figure 5c shows the fluorescence results of the system based on regazyme breakHHRzRAJ12 for all possible combinations of inducers (IPTG and aTc). This logic circuit could further be expanded to integrate more inputs by replacing the constitutive promoter of the regazyme to other tunable promoter. To exclude the possibility that the sRNA Break1 could directly activate the *cis*-repressed reporter gene, we generated a control system (breakRAJ12) by using a dysfunctional mutant removing the regazyme element (Figure 5b and Supplementary Figure S13), revealing no significant riboregulatory activity in this case (Figure 5c).

All together, these results demonstrate the modularity of our designs: (i) the same sensor module can transduce the signal to different mediators (riboregulators), and (ii) the same riboregulator (RAJ12 in this case) can be coupled with different sensor modules. We also analyzed experimentally the orthogonality between regazymes. To this end, we constructed new genetic systems based on non-cognate pairs (between riboregulators and 5′ UTRs), in order to test *in vivo* the eventual cross-talk in regulation of gene expression (36). Computational predictions showed no interference between the riboregulators RAJ11, RAJ12 and RR12 (Supplementary Figure S22a), and previous experimental work revealed no apparent activation of sRNA of system RAJ11 on the 5′ UTR of system RAJ12 (18). However, as shown in Supplementary Figure S22b, signaling cross-talk through regazymes can appear (e.g. between RAJ12 and RR12 riboregulatory systems), probably, as a consequence of non-Watson-Crick pairing not covered in the physicochemical model. A further computational design methodology will account for RNA 3D models to better predict RNA-RNA interaction (7) and then engineer RNA circuits with multiple wires. For *N* different sensor modules and *M* orthogonal riboregulators, we could generate, in theory, *NM* regazymes. Importantly, as the output of one regazyme can be the input of another regazyme, we could have at most (*NM*)*^P^* different implementations of circuits with *P* regazymes, including cascades and feedback loops (Supplementary Figure S23).

### Time-dependent RNA-mediated signal transduction in single cells

**Figure 6.**
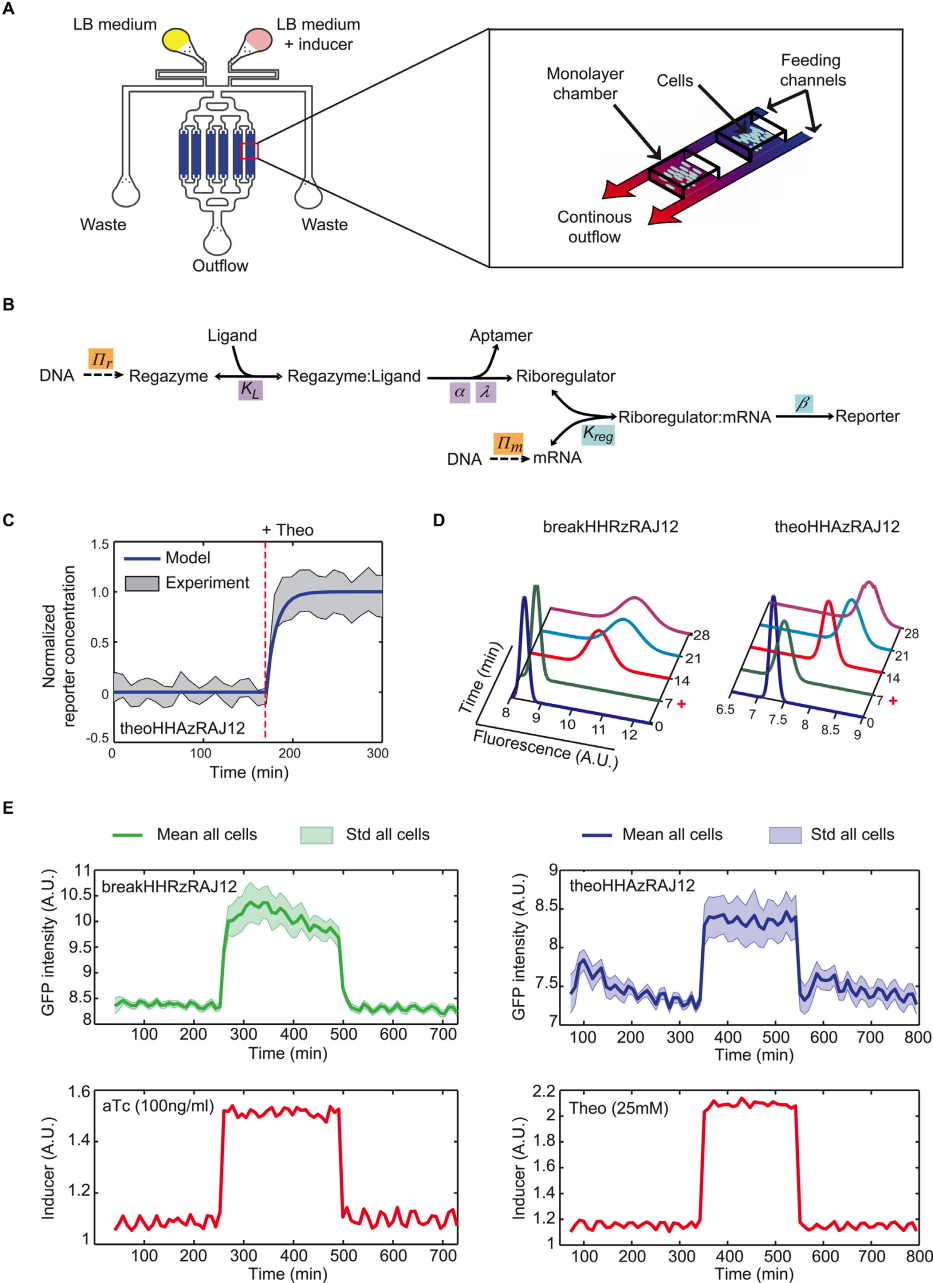
Microfluidics-based single cell analysis of the Theo- and sRNA-sensing regazymes. (**A**) Scheme of the device used in this work to monitor time-dependent GFP expression in single cells. (**B**) Scheme of the reactions of the system, together with the relevant parameters, which serve to construct a mathematical model. (**C**) Prediction of the dynamic response for regazyme theoHHAzRAJ12 (solid line). Shade represents ± one standard deviation from experimental data. (**D**) Distributions of fluorescence with time across a population of cells and fitted with a Gaussian model. Cells were induced at 7 min. (**E**) Single cell tracking in one microchamber of fluorescence under a pulse of aTc (100 ng/ml) for breakHHRzRAJ12 or Theo (25 mM) for theoHHAzRAJ12. A constant amount of IPTG (1 mM) was established during the whole experiment. Sulforhodamine B was used to monitor the inducer time-dependent profile.

To characterize the dynamic response of the designed regazymes at the single cell level, we constructed microfluidics devices according to previous work (36,37). There, single cells were monitored during dozens of cell divisions, using appropriate device geometries to maintain a single layer of cells within the microscope focal plane and a continuous cell growth in exponential phase (Figure 6a, Supplementary Figure S16). Bacterial cells expressing the designed regazymes were loaded into the device, and the composition of the medium was able to incorporate the appropriate time-dependent, small-molecule concentration (25 mM Theo or 100 ng/ml aTc). This way, we can create step functions, pulses or even square waves to force the system. The resulting time series of GFP served to calibrate a mathematical model to further understand the dynamics of the signaling pathway. At this point, we constructed a simplified model, based on first-order kinetics and quasi-steady states assumptions, able to simulate changes in gene expression upon variations in the concentration of the signal molecule (Figure 6b). In essence, the dynamics is modulated by the rates of regazyme cleavage and protein degradation. According to our model, the dynamics can be reduced to an exponential decay with production when the time scale of protein degradation is dominant (Supplementary Figure S21). However, it follows a linear trend in the opposite case. Other parameters such as the cleaved fraction upon signal induction, the effective dissociation constant between the riboregulator and the 5′ UTR, or the gene copy number only affect the stationary level, but not the dynamics. Even though the model can capture small changes in gene expression, the activity could be not discernable in a cellular context when these parameters are suboptimal. Based on our own data, the characteristic times of HHAz (and also HHRz) cleavage and GFP degradation are about 6 and 12 min, respectively (GFP has a degradation tag for riboregulatory devices RAJ12 and RR12). Moreover, the RNA species must also be short lived (about 2 min of half-life) to not exert a significant effect on the dynamics of the systems. In turn, we observed that the response of the system is very fast and without delay, reaching the steady state (criterion of 95%) after 26 min in the case of theoHHAzRAJ12 (Figure 6c). In addition, there is no significant difference in dynamics when inducing with small molecule or sRNA (Figure 6e, see also Supplementary Figures S17 and S18). We also analyzed the dynamic response of regazymes theoHHAzRAJ11 (Supplementary Figure S19) and theoHHAzRR12 (Supplementary Figure S20), showing higher dynamic range in these cases and also higher cell-to-cell variability.

By collecting all single cell measurements, we can analyze the response of the population and its heterogeneity (quantified as the coefficient of variation − CV − in the state ON). The fluorescence is significantly shifted in all cases after addition of the inducers (Figure 6d). Then, we calculated CV = 0.15 for theoHHAzRAJ12 and CV = 0.17 for breakHHRzRAJ12, revealing a similar heterogeneity in the dynamic response in both cases. However, regazyme theoHHAzRR12 has the wider distribution of the fluorescence (with CV = 0.49), which might be a consequence of a higher dynamic range. Indeed, as the enzymatic degradation machinery limits noise in gene expression (and then cell-to-cell variability) (38), high expression levels can saturate this machinery and then expose the system to noise sources related to growth rate (in the case of theoHHAzRR12, such a saturation is indicated by a slow dynamic response, Supplementary Figure S20). Of importance for further developments, the engineered RNA-mediated signaling pathways are highly responsive to multiple variations in the signal molecule.

## DISCUSSION

We have developed a new kind of synthetic RNA molecules (here termed regazymes) able to transduce signals in a modular way in live cells, introducing the one-to-two-component signal transduction paradigm (Figure 1). The design strategy relies on the hierarchical activation/inactivation of RNA elements with specific function. The design of these cascades of interactions requires, in general, automated design algorithms, which we have implemented thanks to an effective energy model. We have exemplified the strategy by designing several regazymes, engaging different sensor modules responsive to small molecules or sRNAs, and also different mediator modules that work as independent regulatory wires. In turn, each mediator regulates a given reporter gene (here a given 5′ UTR of a common mRNA). The sensor and mediator modules are fused with a transducer module (computationally designed) to form the regazyme molecule. Upon sensing the signal molecule, the aptazyme undergoes a conformational change that activates its self-cleavage activity to release the riboregulator, which is fused in the 3′ end (Figures 2 and 3). This riboregulator is then emitted to the cellular context to further activate a downstream *cis*-repressed gene, which may allow the sensing and regulation in different subcellular localizations.

The use of an optimization algorithm, relying on intra- and intermolecular structures and their corresponding free energies as model variables, has allowed us to solve the design problem of the fusion between RNA-based sensors and regulators. However, the computational methods used here to predict free energies and conformational states do not consider three-dimensional contacts neither intermolecular contacts arising in cellular environments (i.e. analysis of the RNA molecules in isolation), which partly limits the predictability of the performance of our designs. By exploring the sequence space, we have identified ideal regions to design functional regazymes without affecting the stability and specificity of the aptamers and riboregulators. The three energetic terms for deriving the objective function have proved effective to capture the functionality of the system (Figure 1). Although we have shown regazymes that sense particular small molecules and sRNAs in live cells, there are more possibilities to widen the scope of their use with the development of new aptazymes. For example, different RNA aptamers sensing chemicals like arginine, nucleotides like flavin, or proteins like HIV-1 Rev (39) encourage the design of the corresponding regazymes by combining computational and experimental screening techniques. This would allow the creation of systems to diagnose the physiological state of a cell, e.g., if it has a new metabolic route in action, if it grows faster than normal, or if it is infected by a virus. In addition, we have demonstrated the versatility of our regazymes by drawing on different riboregulators, with the toehold region in the 5′ end, as mediators. This modularity has the potential to increase the fan-in or fan-out of the system (i.e. the number of inputs and outputs), by disposing in battery regazymes with various sensor domains (signal integration) or with various mediator domains (signal spread). This will result in RNA-mediated signaling hubs. The decoupling of the activities of the aptazyme and riboregulator, besides allowing the straightforward creation of novel systems in a combinatorial way, can enhance the predictability of the dynamic response and the quality of the transmitted information. Similarly, a cleavage-based decoupling of the 5′ UTR sequence from upstream regulatory elements (mainly a promoter) was recently proposed for a protein expression context (40).

Nucleotide-level energy models, such as the one presented in this work, can provide the underlying explanations for molecular interactions, which can then be used in the construction of higher-level biological systems (7). Our study has shown the capability of RNA to mediate in signaling pathways in a cellular context similar to proteins. However, engineering signal transduction with RNA bypasses the challenges related to membrane localization, complex assembly, competition, and limitation of interaction modules regular to protein-based two-component systems (2). Then, the use of regazymes, as pre-engineered modules, may allow devising more complex RNA circuits, at the same time they illustrate new ways to engineer arbitrary complexity. We have shown that a regazyme can be used to engineer multi-input AND logic gates, through the combination of transcriptional and post-transcriptional regulations (Figures 4 and 5). Yet, nothing prevents, in theory, using complementary riboregulatory elements such as RNA-binding/processing proteins (41) or translation-transcription control adaptors (42) to generate feedback or feedforward loops. Therefore, a regazyme could serve as an information transmission platform to control over network connections in live cells, which promises future synthetic biology developments, such as metabolic control and rewiring (9).

In addition, we have measured the dynamic response of the catalytic activity of our designed regazymes in cellular RNA extracts (Figures 2 and 3), obtaining comparable values than those previously measured *in vitro* with the same ribozyme core (11,32). These results have revealed a fast cleavage rate, which is within the same time-scale as protein modifications in synthetic systems (5). Moreover, we have observed higher heterogeneity of the dynamic response (from cell to cell) for the sRNA-sensing regazyme, pointing out an effect of heterogeneous gene expression or even RNA-RNA interaction across cells. Using a mathematical model, we have also analyzed the impact on the dynamics of key parameters governing this RNA-mediated signaling pathway. We have further characterized in detail the dynamic response of the riboregulated gene by monitoring a single cell expressing a regazyme using a microfluidics platform (Figure 6). Systems with higher dynamic range also exhibit higher heterogeneity in their dynamic response (from cell to cell). Our results have shown, in agreement with the catalytic assays, a fast activation of protein expression.

The modularity of our approach, where the sensing and mediator modules can be designed independently through computational design of a proper interface, would allow us to replace the mediator module to control eukaryotic gene expression. Perhaps the best strategy would consist in expressing the regazyme only in the nucleus, using a suitable promoter (e.g. a promoter transcribed by RNA polymerase III). One example would consist in exploiting as mediator module a sRNA able to guide the CRISPR-associated catalytically inactive dCas9 protein to block transcription initiation or elongation (recently proposed as CRISPR interference in both prokaryotes and eukaryotes (43,44)). This RNA-guided DNA targeting would allow the addition of a transcription regulation layer into the RNA-mediated signal transduction. Another example of application in eukaryotic cells would consist in fusing micro RNAs (miRNAs) rather than bacterial riboregulators with sensor domains. There, an aptazyme engineered in the 3′ UTR of the primary miRNA controlling the poly(A) tail could serve to link the mediator to the signal molecule (12). In conclusion, we envision our computationally designed regazymes as a start point for engineering more sophisticated RNA-mediated signaling with particular applications. This would allow the synthetic biology community to use RNA devices to incorporate new signaling functions into cells or rewire natural protein signaling pathways (45).

## SUPPLEMENTARY DATA

Supplementary Data are available at NAR Online.

SUPPLEMENTARY DATA
