# Supplementary material for: Dynamic signal processing by ribozyme-mediated RNA circuits to control gene expression
Source: Nucleic Acids Res. 2015 Apr 27;43(10):5158–70. doi: 10.1093/nar/gkv287 (PMC4446421; doi:10.1093/nar/gkv287)
Supplement: SUPPLEMENTARY DATA [file supp_43_10_5158__index.html]

Dynamic signal processing by ribozyme-mediated RNA circuits to control gene expression — Dynamic signal processing by ribozyme-mediated RNA circuits to control gene expression — SUPPLEMENTARY DATA 

# Dynamic signal processing by ribozyme-mediated RNA circuits to control gene expression

## SUPPLEMENTARY DATA

**Files in this Data Supplement:**

- SUPPLEMENTARY DATA
- SUPPLEMENTARY DATA
- SUPPLEMENTARY DATA
