# Supplementary material for: Dynamic signal processing by ribozyme-mediated RNA circuits to control gene expression
Source: Nucleic Acids Res. 2015 Apr 27;43(10):5158–70. doi: 10.1093/nar/gkv287 (PMC4446421; doi:10.1093/nar/gkv287)
Supplement: SUPPLEMENTARY DATA [file supp_gkv287_nar-01998-y-2014-File008.pdf]

Supplementary Information

Dynamic signal processing by ribozyme-mediated RNA circuits to control gene expression

Shensi Shen[1,†], Guillermo Rodrigo[1,†], Satya Prakash[1], Eszter Majer[2], Thomas E. Landrain[1], Boris Kirov[1], José-Antonio Daròs[2], and Alfonso Jaramillo[1,3,*]

[1] Institute of Systems and Synthetic Biology, Université d'Évry Val d'Essonne, CNRS, F-91000 Évry, France

[2] Instituto de Biología Molecular y Celular de Plantas, CSIC, Universidad Politécnica de Valencia, 46022 València, Spain

[3] School of Life Sciences, University of Warwick, Coventry CV4 7AL, United Kingdom

[†] Equal contribution to the work.

[*] Corresponding author: Alfonso.Jaramillo@warwick.ac.uk

Contents

Supplementary Materials and Methods

Plasmids construction

PCR-based mutagenesis

Strains, reagents and cell cultures

Quantification of *in vivo* fluorescent protein synthesis

Quantification of *in vivo* catalytic activity

Quantification of *in vitro* self-cleavage activity

Microfluidics device construction

Time-lapse microscopy and image analysis

Optimization algorithm

Mathematical model

Mathematical analysis of the dynamic response

Supplementary Figure Legends

Supplementary Figure 1. Maps of the plasmid vectors used in this work for expressing the designed regazymes.

Supplementary Figure 2. Summary of the gene cassettes used in this work.

Supplementary Figure 3. Summary of the signaling pathways generated in this study.

Supplementary Figure 4. Detailed energy landscape in terms of a reaction coordinate.

Supplementary Figure 5. Energy landscape in terms of thermodynamic works.

Supplementary Figure 6. Scheme of the algorithm for regazyme sequence design.

Supplementary Figure 7. Sequences and secondary structures of the aptamers considered in this work.

Supplementary Figure 8. Structural detail of the catalytic reaction of the regazymes.

Supplementary Figure 9. The complete gel images of *in vitro* northern blot corresponding to Figure 2b and 2d.

Supplementary Figure 10. *In vitro* transcribed regazyme self-cleavage assay.

Supplementary Figure 11. Digital diagram of the regazyme theoHHAzRAJ12, theoHHAzRR12 and theoHHAzRAJ11 together with their characterizations.

Supplementary Figure 12. Characterization of regazyme theoHHAzRAJ12 with LacZα reporter.

Supplementary Figure 13. Scheme of the sRNA-sensing regazyme breakHHRzRAJ12 and its dysfunctional mutant.

Supplementary Figure 14. Dose-dependent activation of the three theophylline sensing regazymes.

Supplementary Figure 15. Characterization of regazyme tppHHAzRAJ12.

Supplementary Figure 16. Microfluidics device constructed for this study.

Supplementary Figure 17. Microfluidics-based single cell analysis of the theophylline sensing regazyme theoHHAzRAJ12.

Supplementary Figure 18. Microfluidics-based single cell analysis of the sRNA-sensing regazyme breakHHRzRAJ12.

Supplementary Figure 19. Microfluidics-based single cell analysis of the regazyme theoHHAzRAJ11.

Supplementary Figure 20. Microfluidics-based single cell analysis of the regazyme theoHHAzRR12.

Supplementary Figure 21. Model simulations for different parameter values.

Supplementary Figure 22. Orthogonality analysis between regazymes.

Supplementary Figure 23. Schemes of expanded application of regazyme-based circuits.

Supplementary Tables

Supplementary Table 1. Sequences of regazymes theoHHAzRAJ11, theoHHAzRAJ12 and theoHHAzRR12 designed in this work.

Supplementary Table 2. Sequences of regazymes tppHHAzRAJ12 and breakHHRzRAJ12 designed in this work.

Supplementary Table 3. Sequences of dysfunctional mutant regazymes.

Supplementary Table 4: Sequences and structures of the 5' UTRs.

Supplementary Table 5: Parameter values for the mathematical model.

Supplementary Table 6: Strains and plasmids used in this work.

Supplementary Movies

Supplementary Movie 1. Single cell dynamic response to theophylline of system theoHHAzRAJ12.

Supplementary Movie 2. Single cell dynamic response to aTc of system breakHHRzRAJ12.

Supplementary References

Supplementary Materials and Methods

Plasmids construction

To express and characterize the RNA-based signal transduction, we have generated a modified plasmid vector, pSTC2 containing a pSC101m origin of replication (a mutated pSC101 ori giving a high copy number) and a kanamycin resistance selection marker (**Supplementary Figure 1**). The pSTC2 vector is based on our previously reported vector pSTC1[1] by removing the mRFP coding sequence and tagging the carboxyl terminus of the superfolder GFP (sfGFP)[2] with the *ssr*A degradation tag (ASAANDENYALAA)[3] by polymerase chain reaction (PCR). The underlined AS dipeptide coding sequence corresponds to an NheI restriction site and acts as a linker, while the *ssr*A tag targets proteins to the ClpXP degradation pathway, significantly increasing their degradation rates and therefore dynamic behaviors[3,4].

For engineering our gene cassettes, the pSTC2 vector was made so that independent promoters could drive the expression of the regazyme and the corresponding mRNA. We used the inducible promoters P_{LlacO1} (regulated by LacI and modulated externally by the chemical inhibitor isopropyl-β-D-thiogalactopyranoside, IPTG) and P_{LtetO1} (regulated by TetR and modulated externally by the chemical inhibitor anhydrotetracycline, aTc)[5]. Note that both promoters were placed in opposite directions to avoid transcriptional interference[1]. The regazymes were under the control of the promoter P_{LtetO1}, and the reporter mRNAs (with the *cis*-regulating elements) were under the control of the promoter P_{LlacO1}. For the sRNA-sensing regazyme (breakHHRzRAJ12), the signal sRNA (break1) was placed under control of the promoter P_{LtetO1} and the regazyme was under the control of the constitutive promoter J23119. The sequences of the regazymes and the corresponding 5' UTRs of the targeted mRNAs are presented in the **Supplementary Tables 1-4**. The list of plasmids used in this study is given in the **Supplementary Table 6**. All plasmid manipulations were performed using standard

molecular biology techniques[6]. All enzymes used for plasmid digestions were from Thermo Scientific, USA. All oligonucleotides were synthesized from Integrated DNA Technologies, USA. The different RNA devices (from the terminator of the regazyme to the 5' UTR of the mRNA, see **Supplementary Figure 2**) were chemically synthesized and cloned in plasmid pIDTSMART (pUC replication origin, ampicillin resistance marker) and then subcloned into pSTC1 or pSTC2.

PCR-based mutagenesis

Dysfunctional regazymes (both core catalytic mutations and theophylline binding activity mutations in the aptamers, see **Supplementary Figure 7**) were constructed using PCR-based site-directed mutagenesis with Phusion high fidelity DNA polymerase (Thermo Scientific, USA), followed by template digestion with DpnI (Thermo Scientific, USA) for 1 h at 37 °C. The final products were transformed into chemical competent *E. coli* cells. Mutations were further confirmed by plasmid sequencing (GATC®, Germany). The primers for PCR-based mutagenesis in the theophylline aptamer were 5'-aatccaggacacccgcccagggcctttcggc-3' and 5'-gccgaaaggccctgggcgggtgtcctggatt-3' for theoHHAzRAJ12, 5'-aatccaggacacccgcccagggcctttcggc-3' and 5'-gccgaaaggccctgggcgggtgtcctggatt-3' for theoHHAzRAJ11, and finally 5'-gccgaaaggccctgggcgggtgtcctggatt-3' and 5'-aatccaggacacccgcccagggcctttcggc-3' for theoHHAzRR12. In addition, for breakHHRzRAJ12 we used the following primers 5'-ctcgtcgatccctccctatcagtgatagagattg-3' and 5'-caatctctatcactgatagggagggatcgacgag-3' to excise the regazyme sequence in order to have a dysfunctional system (see **Supplementary Figure 13c**).

Strains, reagents and cell cultures

Strains used in this study are listed in the **Supplementary Table 6**. *E. coli* strain DH5α (Invitrogen, USA) was used for plasmid construction purposes as described in the protocol[6]. Characterization experiments were performed in *E. coli* K-12 JS006 cells (MG1655 Δ*araC* Δ*lacI*)[7], and/or in *E. coli* K-12 MG1655Z1 (or simply MGZ1) cells (MG1655 $lacI^+$ $tetR^+$ $araC^+$ Sp^R)[8] for control over the promoters P_{LlacO1} and P_{LtetO1}. Cells were grown aerobically in Luria-Bertani (LB) broth or in a modified M9 minimum medium, prepared with M9 salts (Sigma, Germany), glycerol (0.8%, vol/vol) as only carbon source, $CaCl_2$ (100 μM), $MgSO_4$ (2 mM), and $FeSO_4$ (100 μM). Cultures were grown overnight at 37 °C and at 225 rpm from single-colony isolates before being diluted for *in vivo* characterization. When appropriate, kanamycin concentration was 50 μg/mL and ampicillin concentration was 100 μg/mL. In the case of MG1655Z1 cells, 1 mM IPTG (Thermo Scientific, USA) was used for full activation of promoter P_{LlacO1} when needed, and 100 ng/mL aTc (Sigma, Germany) was used for full activation of promoter P_{LtetO1}. 4 mM theophylline (Sigma, Germany) and 0.5 mM thiamine hydrochloride (Sigma, Germany) were used for general characterization of the small molecule sensing regazymes in the fluorometer. We also used gradients of aTc, theophylline, and thiamine to perform dose-dependent assays. For microfluidic cell cultures, cells were grown aerobically in fresh LB broth or in LB supplemented with 0.05% sulforhodamine B (Sigma, Germany) and (i) 25 mM theophylline or (ii) 100 ng/mL aTc.

Quantification of *in vivo* fluorescent protein synthesis

Cells were grown overnight in 5 mL of LB medium, and were refreshed in culture tubes with LB medium in order to reach stationary phase. Cells were then diluted 1:200 in 200 μL of M9 minimal medium in each well of the plate (Custom Corning Costar 96 well microplate, black transparent bottom with lid). The plate was incubated in an Infinite F500 multi-well fluorometer (TECAN, Switzerland) at 37 °C with shaking (orbital mode, frequency of 33

rpm, 2 mm of amplitude). It was assayed with an automatic repeating protocol of absorbance measurements (600 nm absorbance filter) and fluorescence measurements (480/20 nm excitation filter - 530/25 nm emission filter for sfGFP) every 15 min. All samples were present in triplicate on the plate. Each measurement was repeated on independent days to verify reproducibility. All data analyses were done using values harvested when cells were in exponential growth phase (OD_{600} between 0.1 and 0.6). Growth rates were calculated as the slope of a linear regression between the values of ln(OD_{600}) and time. The normalized fluorescence was obtained by two methods: (*i*) as the slope of the linear regression between the values of absolute fluorescence (*F*) and OD_{600}, and (*ii*) as the ratio between absolute fluorescence and OD_{600} (i.e., $\frac{F-\langle F_{\text{medium}}\rangle}{OD-\langle OD_{\text{medium}}\rangle}$, where *brakets* denote average per samples). The normalized fluorescence of plain cells (transformed with a plasmid without GFP) was also considered for background subtraction when appropriate, and then obtaining the stationary protein expression value (magnitude per cell).

Quantification of *in vivo* catalytic activity

To *in vivo* quantify the catalytic activity of the two versions of the RAJ12-based regazyme that sense theophylline (theoHHAzRAJ12) and an effector RNA (breakHHRzRAJ12), we transformed *E. coli* (strain T7 Express in the case of theoHHAzRAJ12 and strain MG1655Z1 in the case of breakHHRzRAJ12) with the appropriate plasmids (pSCKtheoRAJ12 for theophylline-induced cleavage, and pUAbreak12 and pSCKbreak12 for sRNA-induced cleavage). Overnight cultures were grown at 28 ºC by inoculating LB liquid media containing 50 μg/mL kanamycin (theoHHAzRAJ12) or 50 μg/mL kanamycin and 50 μg/mL spectinomycin (breakHHRzRAJ12) with three different colonies in each case. These overnight cultures were used to inoculate new liquid cultures with 50 mL of the corresponding media at 0.1 OD_{600} and grown at 37 ºC to reach 0.6 OD_{600}. At this point each

culture was split into three aliquots of 15 mL, and theophylline or aTc was added when appropriate to obtain cultures with 0, 0.4 and 4 mM theophylline or 0, 10 and 100 ng/mL aTc. Incubation was continued at 37 ºC with shaking and 2 mL aliquots were taken at 0, 2, 4, 8, 16 and 32 min time points. Bacteria in the 2-mL aliquots were quickly pelleted by centrifuging for 2 min at 13,000 rpm and re-suspended in 50 µL of TE (10 mM Tris-HCl, pH 8.0, 1 mM EDTA). Bacteria were broken by adding 50 µL of a 1:1 phenol:chloroform (pH 8.0) mix and vortexing thoroughly. Bacterial RNA from each sample was recovered in the aqueous phase by centrifuging for 5 min at 13,000 rpm, re-extracted with 50 µL chloroform.

Processing extent of regazyme in each aliquot was analyzed by northern blot hybridization using a complementary [^{32}P]-labelled RNA probe after separating the different RNA samples by denaturing polyacrylamide gel electrophoresis (PAGE). 20 µL of the RNA preparations were mixed with one volume of formamide loading buffer (98% formamide, 10 mM Tris-HCl, pH 8.0, 1 mM EDTA, 0.0025% bromophenol blue, and 0.0025% xylene cyanol), denatured for 1.5 min at 95 ºC and snap cooled on ice. After this treatment, samples were separated by PAGE in 5% polyacrylamide (37.5:1 acrylamide:N,N'-methylenebisacrylamide) gels of 140 x 130 x 2 mm including 8 M urea and TBE buffer (89 mM Tris, 89 mM boric acid, 2 mM EDTA) for 1.5 h at 200 V. Gels were stained with ethidium bromide, photographed under UV light and the RNAs were electroblotted to positively charged nylon membranes (Nytran SPC; Whatman, USA) and cross-linked by irradiation with 1.2 J/cm^2 UV light (Vilber Lourmat). Membranes were hybridized overnight at 70 ºC in 50% formamide, 0.1% Ficoll, 0.1% polyvinylpyrrolidone, 100 ng/mL salmon sperm DNA, 1% SDS, 0.75 M NaCl, 75 mM sodium citrate, pH 7.0, and 10^5 cpm/mL of the [^{32}P]-labelled complementary RNA probe. Membranes were washed three times for 10 min with 2 x SSC (SSC is 150 mM NaCl, 15 mM sodium citrate, pH 7.0), 0.1% SDS at room temperature and once for 15 min at 55 ºC with 0.1 x SSC, 0.1% SDS. Membranes were

imaged by autoradiography and the hybridization signals quantified by phosphorimetry (Fujifilm FLA-5100, Japan).

To produce the [^{32}P]-labelled RNA probe in order to quantify regazyme processing, we firstly amplified by PCR a fragment of the regazyme cDNA using primers 5’-TGGCGCTGCCTTCGTACATCC-3’ and 5’-ACAGAAAAGCCCGCCTTTCGA-3’. The probe corresponds to the reverse complement of regazyme theoHHAzRAJ12 and was used to detect both the regazymes and cleaved products of systems theoHHAzRAJ12 and breakHHRzRAJ12. This cDNA was cloned in the appropriate orientation into a pUC18 (L08752.1)-derived plasmid flanked by a bacteriophage T3 RNA polymerase promoter and an XbaI restriction site. The *in vitro* transcription reaction consisted of 1 μg of the XbaI-linearized plasmid, 2 mM each ATP, CTP and GTP, 70 μCi of [α-^{32}P] UTP (800 Ci/mmol), 40 mM Tris-HCl (pH 8.0), 6 mM $MgCl_2$, 20 mM DTT, 2 mM spermidine, 20 U RNase inhibitor (Ribolock, Thermo Scientific, USA), 0.1 U yeast inorganic pyrophosphatase (Thermo Scientific, USA) and 50 U of T3 RNA polymerase (Epicentre, USA) in a final volume of 20 μL The reaction was incubated for 2 h at 37 ºC. Then, 10 U of DNase I (Thermo Scientific, USA) were added and incubated at 37 ºC continued for 10 min. The probe was finally purified by chromatography using a Sephadex G-50 spin column (Mini Quick Spin Column, Roche Applied Science) and quantified by Cerenkov.

Quantification of *in vitro* self-cleavage activity

We cloned the regazymes theoHHAzRAJ12 and breakHHRzRAJ12 (without transcription terminators) into new plasmids (pUC-like) under the control of a T3 promoter and by adding the sequences GGGAT in the 5' end and ATCTCTAG (this is XbaI restriction site) in the 3' end. Linearization of the plasmids was done with XbaI, followed by purification with silica-based columns (ZYMO). Reactions of *in vitro* transcription were carried out without and with

(4 mM) theophylline in case of theoHHAzRAJ12, and without and with (3 μM) the DNA oligo Break1 in case of breakHHRzRAJ12. 100 ng and 70 ng of plamids having regazymes theoHHAzRAJ12 and breakHHRzRAJ12 were used for the reactions, respectively. Reaction in a final volume of 20 μL was done with 2 μL transcriptase buffer 10x, 0.4 μL 0.5 M DTT, 1 μL 10 mM NTPs, 0.5 μL RNase inhibitor Ribolock (Thermo Scientific, 40 U/μL), 1 μL inorganic pyrophosphatase (Thermo Scientific, 0.1 U/μL), 1 μL T3 RNA polymerase (Roche, 20 U/μL). We incubated for 30 min at 37 °C. Then we added 20 μL (1 vol.) formamide buffer to stop the reaction. Samples were then heated 1.5 min at 95 ºC, followed by storage on ice. To load the gel (5% PAGE, 8 M urea, TBE 1x), we mixed half of the resulting sample (10 μL) with the appropriate buffer (10 μL). The conditions were 200 V and 1.5 h. We used the Thermo Scientific RiboRuler Low Range RNA Ladder.

For the *in vitro* time-course of self-cleavage assay, plasmid containing regazyme theoHHAzRAJ12 was linearized with XbaI and transcribed with T3 RNA polymerase in the presence of high NTPs concentration (2 mM each) to sequester free Mg^{2+} in the reaction and inhibit regazyme self-cleavage. Transcription products were separated with denaturing PAGE, and the uncleaved regazyme eluted from the gel by diffusion in the presence of 10 mM EDTA. Regazyme was precipitated with ethanol and finally resuspended in 50 mM Tris-HCl, pH 8.0, 1 mM EDTA. A time-course self-cleavage experiment in the presence or not of 4 mM theophylline was performed. Both reactions were started by adding $MgCl_2$ to 5 mM and aliquots taken at 0, 1, 2, 4, 8 and 16 min. Reactions were stopped using a buffer including formamide and EDTA. Finally, reaction products in the different aliquots were separated with denaturing PAGE in the presence of 8 M urea. The gel was stained with ethidium bromide and the bands corresponding to the uncleaved regazyme and the cleavage products quantified through the fluorescent emission under ultraviolet irradiation. A similar procedure was carried out to analyze the self-cleavage activity of regazyme breakHHRzRAJ12.

Microfluidics device construction

To understand the dynamic regulation of our regazyme devices, we have examined *in vivo* gene expression with single-cell, time-lapse fluorescence microscopy using a microfluidics device (**Supplementary Figure 16a**). This was designed to support monolayer growth of *E.coli* cells under constant nutrient flow. By coupling with cell tracking and fluorescence measurement, our microfluidics device allows us to generate fluorescence trajectories for single cells. The design of the microfluidics device was adapted from the previous one reported by Hasty and coworkers[7,9]. In brief, the *E. coli* cells were loaded from the cell inlet while keeping the media inlet at sufficiently high pressure to avoid contamination. Cells were loaded into the microchambers by manually applying pressure pulses to the syringe lines to induce a momentary flow change. After cell loading, the flow was then reversed to allow cells receiving fresh media with 0.075% Tween20 which prevented cells from adhering to the main channels and waste outlets. The microfluidics device contained three parallel channels with 10 μm height. Each channels contained two subchannels with microchambers (1 μm height) in the middle of them, which allowed the out growing cells been washed away by the flow. The width of the parallel chamber was limited to 30 μm to avoid the risk of the PDMS structural collapse of the chamber ceilings. For optimal *E. coli* growth, the microfluidics chip temperature was typically maintained at 37 °C by external tempcontrol system (PECON, Germany). For on-chip induction experiments, we used two media inlets (for two different input media, with and without chemical inducer), which directed the flow to the cells from one to another by means of pressure changes. For induction flow (medium with inducer), the media input speed was kept at 500 μL/h, and for relaxation flow (medium without inducer) was kept at 10 μL/h.

The design of the microfluidics device was performed in AUTOCAD software (AUTODESK, USA), and the printed wafer was fabricated by Veeco Instrument GmbH

(Veeco, Germany). Replica molds were created *in house* from master molds by mixing polydimethylsiloxane (PDMS)/Sylgard 184 (Dow Corning, USA) in a 10:1 ratio of elastomer base vs. curing agent. The molds were degassed by briefly centrifuge at 4000 rpm for 5 min, followed by degassing in a vacuum desiccator at -1 a.t.m. for 30 min, and curing in place over the master at 80 °C for 2 h. After removal of the PDMS monolith, chips were sectioned, bored at the fluidic ports in a clean culture hood, and then bonded to clean coverslips (Corning, USA) by exposure to O_2 plasma at 30 W for 30 s in a plasma asher (ACE-1, GaLa Instrumente, Germany). The bonded chips were cured in 80 °C incubator for at least 2 days before experiments.

Time-lapse microscopy and image analysis

All images were acquired using Zeiss Axio Observer Z1 microscopy (Zeiss, Germany), comprising a Pln Apo 100X/1.4 Oil Ph3 DICIII objective outfitted with fluorescence excitation and emission filter wheels (including 38HE GFP RL, 46HE YFP RL, 47HE CFP RL, and 64HE RFP RL). The microscope resolution was 0.24 μm with Optovariation 1.6X, resulting total magnification 1600X for both bright field and fluorescent images. The microscopy equipped with an X-Cite Series 120 fluorescent lamp (EXFO, illumination) and a HAMAMATSU EM-CCD C9100 digital camera (Hamamatsu Photonics, Japan) automated by a commercial application software (AxioVs 40 V4.8.1.0, Zeiss, Germany).

In each experiment, the microfluidics device mentioned above was mounted to the stage and loaded with *E. coli* cells. Trapped cells were allowed to grow overnight with normal LB medium flow at 500 μL/h. During exponential growth of the monolayer colony, images were collected at 100X magnification in the phase contrast every 30 s and GFP or RFP fluorescence channels every 7 min (GFP channel for monitoring the gene expression, RFP channel for monitoring the inducer diffusion) over a period of around 24 h (**Supplementary**

Figures 16b and 16c). In each experimental run, we have chosen four different cell microchambers to follow the gene expression dynamics. Focus was predefined by focus setting with contrast-based autofocus algorithms. Images were extracted and movies were generated by *in house* processing based MATLAB (MathWorks, USA) for both phase contrast and fluorescent frames respectively. Briefly, inverted phase contrast movies were used for threshold settings, followed by generating the binary threshold phase-contrast movies. To compensate the binary movies for minor movements of different frames due to the microscopy, large objects were found in order to track the stability. After this correction, the resulting binary image was processed with morphological distance transformation and watershed segmentation. Cells were tracked by defining a cell-to-cell distance matrix and the cell lineages were reconstructed. Finally the fluorescence level of each cell in each fluorescence frame was extracted.

Optimization algorithm

We developed an optimization algorithm to design regazymes provided the sequences of a given aptazyme and a riboregulator. On the one hand, the aptazyme responds to its ligand to cleave the RNA sequence at a given point[10]. On the other hand, the riboregulator is able to activate protein expression by inducing a conformational change in the 5' UTR of the mRNA. The sequence of a regazyme is composed of prefix and suffix sequences flanking the aptazyme followed by the riboregulator (**Supplementary Figure 6a**). These sequences constitute part of the transducer module. The aptazyme and riboregulator sequences are kept fixed. The only premise for the design is that the riboregulator needs to have the seed region in its 5' tail. Hence, the prefix and suffix are designed to get the seed paired within the structure of the full regazyme (state OFF), and unpaired (and then exposed to the solvent for RNA-RNA interaction) within the resulting structure after cleavage induced by the ligand (state ON). These sequences are totally variable in nucleotide composition and length.

Starting from random sequences for the prefix and suffix, the algorithm implements a heuristic optimization based on Monte Carlo Simulated Annealing (**Supplementary Figure 6b**)[10]. At each step, random mutations consisting in replacements, additions, or deletions are applied to evolve the sequence, and then selected with an objective function (ΔG_{score}). This way, we constructed the minimization problem

$$\min \Delta G_{\text{score}} = \Delta G_{\text{act}}^{\text{aptamer}} - \Delta G_{\text{act}}^{\text{uncleaved}} + \Delta G_{\text{act}}^{\text{cleaved}} \propto W_{\text{aptamer formed}}\Big|_{\substack{\text{before}\\\text{cleavage}}} + W_{\text{seed paired}}\Big|_{\substack{\text{before}\\\text{cleavage}}} + W_{\text{seed unpaired}}\Big|_{\substack{\text{after}\\\text{cleavage}}} \qquad \text{(Eq. S1)}$$

where W is the thermodynamic work required to get the aptamer formed to sense the ligand or get the seed unpaired or paired, and it is assumed to be proportional to the activation free energy (ΔG_{act}). We balanced equally these states. Denoting by Λ and Γ_0 the structures of the aptamer and seed within the regazyme before cleavage, and by Γ the structure of the seed after cleavage, we can calculate these works by

$$\begin{aligned} W_{\text{aptamer formed}}\Big|_{\substack{\text{before}\\\text{cleavage}}} &= G_n d(\Lambda, \text{Paired}) \\ W_{\text{seed paired}}\Big|_{\substack{\text{before}\\\text{cleavage}}} &= G_n d(\Gamma_0, \text{Paired}) \\ W_{\text{seed unpaired}}\Big|_{\substack{\text{after}\\\text{cleavage}}} &= G_n d(\Gamma, \text{Unpaired}) \end{aligned} \qquad \text{(Eq. S2)}$$

where G_n is the average free energy contribution per nucleotide (here we consider G_n = 1.28 Kcal/mol), and d is the Hamming distance between two secondary structures.

Typically, the convergence of the algorithm is fast, obtaining sequences with ΔG_{score} = 0 in some seconds or minutes. We used the Vienna RNA package with default parameters[11]. Our systems are based on conformational changes, but algorithms for multi-state RNA design are scarce. Our approach tackles this problem, allowing sequence and structure specifications, and exploiting RNA folding algorithms such as Vienna RNA. In this work, we just focused on the computational design of the transducer module, but nothing would prevent a full design of the molecule, including the riboregulator. A strategy of nesting design processes (one for the riboregulator and another for the regazyme) enhances the convergence of the corresponding

algorithms, as well as makes the designs more modular. To this end, our approach has the advantage of leaving unconstrained the sequence length. However, our approach has the limitation of just using 2D structure to model RNA conformational change and catalysis. Certainly, this type of mechanisms could involve pseudoknot interactions and even non-canonical base pairs, for which 3D models could better capture the interaction and processing features. Nevertheless, although the ribozyme has tertiary contacts, the exposition or blockage of the seed region in our design is governed by secondary structure. In addition, our model does not take into account kinetic binding effects, which might have an impact on the designs.

Mathematical model

The full set of biochemical reactions (constants in brackets) of the system theoHHAzRAJ12 (small molecule sensing) is

Ø → Regazyme (Π_r)

Regazyme → Ø ($\mu+\delta_r$)

Ø → mRNA (Π_m)

mRNA → Ø ($\mu+\delta_m$)

Theo + Regazyme ↔ Theo:Regazyme (k_{-theo} / k_{theo})

Theo:Regazyme → Ø ($\mu+\delta_r$)

Theo:Regazyme → Theo:Aptamer:Riboregulator (γ)

Regazyme → Aptamer:Riboregulator (γ_0)

Theo:Aptamer:Riboregulator → Ø ($\mu+\delta_r$)

Aptamer:Riboregulator → Ø ($\mu+\delta_r$)

Theo:Aptamer:Riboregulator ↔ Theo:Aptamer + Riboregulator (k_{-rel} / k_{rel})

Aptamer:Riboregulator ↔ Aptamer + Riboregulator (k_{-rel} / k_{rel})

Theo:Aptamer → Ø ($\mu+\delta_r$)

Aptamer → Ø ($\mu+\delta_r$)

Riboregulator → Ø ($\mu+\delta_s$)

Riboregulator + mRNA ←→ Riboregulator:mRNA (k_{-reg} / k_{reg})

Riboregulator:mRNA → Ø ($\mu+\delta_s$)

Riboregulator:mRNA → Riboregulator:mRNA + GFP (β)

mRNA → mRNA + GFP (β_0)

GFP → Ø ($\mu+\delta_g$)

Clearly, this model can be easily rewritten in case of sRNA sensing. Then, to quantitatively model the protein synthesis in the cells, we could construct a system of differential equations based on those reactions. However, due to the lack of reliable values for many of the parameters, we decided to take a quasi-steady state approach.

We first assumed that the global and intracellular concentration of external inducers is the same, and that they bind very fast (relative to other time scales in the system) to their target molecules (i.e., IPTG to LacI, aTc to TetR, and theophylline –Theo– to RNA aptamer). Therefore, the total concentrations of RNAs are taken in quasi-steady state, given by

$$[mRNA]_{total} = \frac{N\Pi_m}{\delta_m+\mu}\frac{\frac{1}{f_i}+\left(\frac{[IPTG]}{K_i}\right)^{n_i}}{1+\left(\frac{[IPTG]}{K_i}\right)^{n_i}}$$

$$[Regazyme]_{total} = \frac{N\Pi_r}{\delta_r+\mu}\frac{\frac{1}{f_a}+\left(\frac{[aTc]}{K_a}\right)^{n_a}}{1+\left(\frac{[aTc]}{K_a}\right)^{n_a}} \qquad \text{(Eqs. S3)}$$

where IPTG and aTc can vary with time. N is the plasmid copy number. Π_x (x = m or r) is the maximal transcription rate of the transcript (mRNA or regazyme), and δ_x (x = m or r) the corresponding degradation rate (mRNA or regazyme). The growth rate of the cells is μ. In addition, K_x (x = i or a) is the effective regulatory constant of the inducer (IPTG or aTc) that

inhibits the repressor action (of LacI or TetR), and n_x (x = i or a) the effective Hill coefficient (for IPTG or aTc).

Furthermore, because the concentration of theophylline is much higher than the one of regazyme, we can write

$$\left[Theo:Regazyme\right]=\frac{\left[Theo\right]}{K_{theo}+\left[Theo\right]}\left[Regazyme\right]_{total} \quad \text{(Eq. S4)}$$

where K_{theo} is the effective dissociation constant *in vivo* between the aptazyme and theophylline (i.e., $K_{theo} = k_{-theo} / k_{theo}$). The concentration of free regazyme is then [*Regazyme*] = $[Regazyme]_{total}$ – [*Theo:Regazyme*].

To simplify the model of the aptazyme cleavage, we introduce the following term

$$\left[Riboregulator\right]=\alpha_0\left[Regazyme\right]+\left(\alpha-\alpha_0\right)\left(1-e^{-\lambda t}\right)\left[Theo:Regazyme\right] \quad \text{(Eq. S5)}$$

where α_0 is the fraction of cleavage in absence of any ligand and α the maximal fraction in presence of theophylline. Moreover, λ is rate at which the catalytic reaction takes place (it is related to γ). Previous to the addition of theophylline, we can write [*Regazyme*] = $[Regazyme]_{total}$. After the addition of theophylline in high amount, we can consider [*Theo:Regazyme*] = $[Regazyme]_{total}$.

Once the riboregulator is released, it can interact with its mRNA target. We assume this reaction is faster than that of cleavage and does not introduce any delay. Hence, the concentration of the resulting complex is

$$\left[Riboregulator:mRNA\right]=$$
$$=\frac{1}{2}\left(\left[Riboregulator\right]+\left[mRNA\right]_{total}+K_{reg}\right)\left(1-\sqrt{1-\frac{4\left[Riboregulator\right]\left[mRNA\right]_{total}}{\left(\left[Riboregulator\right]+\left[mRNA\right]_{total}+K_{reg}\right)^2}}\right) \quad \text{(Eq. S6)}$$

where K_{reg} is the effective dissociation constant *in vivo* between the riboregulator and mRNA (i.e., $K_{reg} = k_{-reg} / k_{reg}$). The concentration of free mRNA is then [*mRNA*] = $[mRNA]_{total}$ – [*Riboregulator:mRNA*]. Therefore, the synthesis of GFP is governed by

$$\frac{d}{dt}[GFP] = \beta_0[mRNA] + \beta[Riboregulator:mRNA] - (\delta_g + \mu)[GFP] \qquad \text{(Eq. S7)}$$

where β_0 is the translation rate in absence of riboregulator and β the rate in presence of it. Finally, δ_g is the first-order protein degradation rate. $[GFP]_0 = 0$ could be taken as initial condition *in vitro*, but because the system is expressed in a cellular context we should take $[GFP]_0 = \beta[Riboregulator:mRNA]/(\delta_g + \mu)$ calculated with $[Riboregulator] = \alpha_0 M$.

Mathematical analysis of the dynamic response

To analyze the dynamic response of the regazyme system, we assume that the only inducer with time dependence is theophylline and that the total concentrations of mRNA and regazyme are in steady state. Thus, from Eqs. (S3) and considering $\delta_m = \delta_r$, we have

$$\begin{aligned} [mRNA]_{total} &= \frac{N\Pi_m}{\delta_m + \mu} = M \\ [Regazyme]_{total} &= \frac{N\Pi_r}{\delta_r + \mu} = M \end{aligned} \qquad \text{(Eqs. S8)}$$

where $M \approx 3000$ nM for a particular set of parameter values (**Supplementary Table 5**). We also assume that $[Theo] >> K_{theo}$ to write Eq. (S5) as

$$[Riboregulator] = \left(\alpha_0 + (\alpha - \alpha_0)\left(1 - e^{-\lambda t}\right)\right)M \qquad \text{(Eq. S9)}$$

In case of producing directly a riboregulator from promoter P_{LtetO1}, we would have $[Riboregulator] = M$. In addition, because the *cis*-repression is very efficient[1] and GFP has a degradation tag, we can take $\beta_0 << \beta$ and $\delta_g >> \mu$. Finally, we can write the time dependence of GFP as

$$[GFP](t) = [GFP]_0 e^{-\delta_g t} + \beta e^{-\delta_g t}\int_0^t e^{\delta_g \tau}[Riboregulator:mRNA](\tau)d\tau \qquad \text{(Eq. S10)}$$

to be integrated numerically in combination with Eqs (S6) and (S9).

Supplementary Figure Legends

Supplementary Figure 1: Maps of the plasmid vectors used in this work for expressing the designed regazymes. (**a**) Plasmid vector map for gene synthesis of the regazymes used in this study. This vector contains a high copy number pUC replication origin and an ampicillin resistance marker. (**b**) pSTC2 vector map. (**c**) pSTC1 vector map. Gene cassettes from pSynth can be cloned into these vectors by using restriction digestion with EcoRI and SpeI. Except otherwise indicated, regazymes were under the control of the inducible promoter P_{LtetO1}, and the reporter mRNAs (with the *cis*-regulating elements) were under the control of the inducible promoter P_{LlacO1}. Vectors pSTC1 and pSTC2 contain a high copy number pSC101m replication origin (mutated version of pSC101) and a kanamycin resistance marker. Maps were constructed with the free software Savvy (http://bioinformatics.org/savvy/).

Supplementary Figure 2: Summary of the gene cassettes used in this work. (**a**) Four small-molecule sensing regazymes were synthesized as indicated in the left panel. The right panel shows the corresponding two-nucleotide inactivation mutants generated by site-directed mutagenesis (see **Supplementary Materials and Methods**). (**b**) One sRNA-sensing regazyme was synthesized as indicated, and a control system. Here, the signal sRNA was placed under the control of P_{LtetO1}, the regazyme under the control of the constitutive promoter J23119. (**c**) The gene cassettes used in the orthogonality assay were constructed by using restriction enzyme digestion and T4 ligation. We generated six combinations of regazymes and *cis*-regulators. Maps were constructed with the free software Savvy (http://bioinformatics.org/savvy/).

Supplementary Figure 3: Summary of the signaling pathways generated in this study. (**a**) Regazymes were shown as ellipses with two different colors that correspond to the sensor domain (aptazyme) and the actuator domain (riboregulator). The input signals were two small molecules: theophylline (Theo) and thiamine pyrophosphate (TPP), and one sRNA: Break1. The actuators corresponded to three different riboregulators: sRNAs from systems RR12, RAJ11, and RAJ12, which link downstream to their corresponding *cis*-repressed gene expression platforms. (**b**) Illustration of a regazyme exploited for sensing and transducing environmental signals.

Supplementary Figure 4: Detailed energy landscape in terms of a reaction coordinate. The different intra- and intermolecular folding states are shown, as well as the cleaved and uncleaved states. All free energies gaps are illustrated. Three different trajectories over the landscape can be followed. One corresponds to the ligand-induced cleavage of the regazyme and subsequent binding of the riboregulator to the mRNA (solid line). Another corresponds to the natural self-cleavage of the regazyme (in absence of ligand) and also subsequent binding of the riboregulator (dotted line).For indication purposes, we present this trajectory in the same landscape, although the associated reaction coordinate would be different. And, finally, another corresponds to the eventual binding of the regazyme (in absence of cleavage) to the mRNA (dashed line).

Supplementary Figure 5: Energy landscape in terms of thermodynamic works (W, see **Supplementary Materials and Methods**). We represent planar projections of main **Figure 1d**, together with the distributions of the different works (to get the seed paired and the aptamer formed before cleavage, and the seed unpaired after cleavage).

Supplementary Figure 6: Scheme of the algorithm for regazyme sequence design. (**a**) Modular sequence of a regazyme. It is composed of prefix and suffix sequences flanking an aptazyme plus a riboregulator. The aptazyme and riboregulator sequences are kept fixed. The prefix and suffix are designed to get the intended regulation: in our case, to produce a functional riboregulator after ligand-induced cleavage. (**b**) Optimization scheme for sequence design. Starting from random sequences for the prefix and suffix, the algorithm implements a heuristic optimization (based on Monte Carlo Simulated Annealing) where random mutations (involving replacements, additions, or deletions) are applied and selected with an objective function.

Supplementary Figure 7: Sequences and secondary structures of the aptamers considered in this work. We designed regazymes with (**a**) theophylline and (**b**) thiamine pyrophosphate aptamers. In addition, we constructed three different types of mutants in the theophylline aptamer, indicated as arrows in (**a**), as a negative control and also to study the natural self-cleavage of the regazyme. This way, we affect the core catalytic activity of ribozyme (blue arrow A to G mutation in **a**), inactivating ribozyme (red arrow U to G mutation in **a**) and binding affinity to the ligand (orange arrow U, A to C mutation in **a**).

Supplementary Figure 8: Structural detail of the catalytic reaction of the regazymes. The catalytic site (for self-cleavage) was shown in blue, and marked with an arrow. In the OFF state, the seed (shown in red) is complementary paired. However, in the ON state (after cleavage), it is unpaired and then exposed to the solvent. The secondary structures were predicted by NUPACK (without pseudoknots)[12] and then plotted with VARNA[13]. (**a**) theoHHAzRAJ11; (**b**) theoHHAzRAJ12; and (**c**) theoHHAzRR12.

Supplementary Figure 9. The complete gel images of *in vitro* northern blot corresponding to Figures 2 and 3. RNA extracts from bacterial cells treated with ligands for indicated time points were processed with [^{32}P]-labelled RNA probes that recognize regazyme sequences, and separated on northern blot for quantification of the cleavage detailed in **Supplementary Materials and Methods**. (**a**) theoHHAzRAJ12; (**b**) breakHHRzRAJ12. The strong secondary structure of the RNAs provokes a slight displacement with respect to the markers. Note that the probe is the same for the two gels and corresponds to the reverse complement of regazyme theoHHAzRAJ12. In (**a**), comparison with *in vitro* results suggests that the 5' fragment is quickly degraded *in vivo*. In (**b**), only one product is detected (riboregulator) as the probe cannot reveal the 5' fragment. The products were shown by red dashed box. Note that these gels have been repeated three times each, obtaining always the same results. On the one hand, regazyme theoHHAzRAJ12 has a length of 194 nt. The band corresponding to the riboregulator released after cleavage, of 114 nt, appears to migrate faster. On the other hand, regazyme breakHHRzRAJ12 has a length of 196 nt. The band corresponding to the riboregulator released after cleavage, of 112 nt, migrates a bit slower. Our results suggest nevertheless that these bands correspond to products of the cleavage reactions in the presence of the ligands (no bands are seen in absence of them).

Supplementary Figure 10. *In vitro* transcribed regazyme self-cleavage assay. (**a**) Regazymes theoHHAzRAJ12 and breakHHRzRAJ12 were *in vitro* transcribed with or without corresponding ligands (theo: 4 mM theophylline; oligo: 3 μM Break1 oligo). The products were separated on PAGE gels followed by ethidium bromide. For theoHHAzRAJ12 two bands (85nt and 92nt) were expected as cleaved products, however, for breakHHRzRAJ12 two bands (89nt and 90nt) were expected as cleaved products. Quantification was shown in the right panel based on the mass ratio between cleaved products

and precursors. M: RNA ladders. (**b,c**) *In vitro* time-course of self-cleavage activity assay for regazyme theoHHAzRAJ12 (**b**) and breakHHRzRAJ12 (**c**). The self-cleavage activity was monitored with denaturing PAGE in the presence or absence of ligands at indicated time points. The quantification of cleavage ratio (cleaved / total) is shown here. In the *in vitro* assay, RNA is not produced nor degraded during the cleavage, whereas *in vivo* RNA is produced, degraded and cleaved at the same time. Therefore, the expected kinetics are different. The cleaved fraction increases with time upon the addition of the ligand. Without the presence of ligand, the regazyme is still cleaved *in vitro* (leakage activity), but *in vivo* system is in a steady state. This is also shown by our mathematical model for the dynamics of the system species *in vivo*.

Supplementary Figure 11: Digital diagram of the regazyme theoHHAzRAJ12, theoHHAzRR12 and theoHHAzRAJ11 together with their characterizations. We present the results of GFP expression (population level) of MG1655Z1 cells expressing Regazyme or Regazyme mutation as control. These were characterized under different combinations of inducers. The truth table is shown in agreement to the experimental data. (**a**) theoHHAzRAJ12 and theoHHAzRAJ12Cm (corresponding to inactivating mutation as shown in **Supplementary Figure 7a**). (**b**) theoHHAzRR12 and theoHHAzRR12Cm. (**c**) Core catalytic mutation of theoHHAzRAJ12 (theoHHAzRAJ12AGm, see also **Supplementary Figure 7a**) was analyzed in JS006 strain with different concentrations of theophylline. (**d**) theoHHAzRAJ11 and theoHHAzRAJ11mut (corresponding to theophylline binding activity mutation as shown in **Supplementary Figure 7a**). The concentrations of inducers were: aTc (100 ng/mL), IPTG (1 mM), and Theo (4 mM). Error bars were standard deviations of three replicates.

Supplementary Figure 12: Characterization of regazyme theoHHAzRAJ12 with LacZα reporter. (**a**) The regazyme and reporter gene were synthesized in the commercial plasmid vector pSynth. DH5αZ1 strain was transformed with the plasmid. (**b**) Experimental results. The expression of the reporter gene was characterized thanks to the LacZ enzymatic activity by using the 4-Methylumbelliferyl-β-D-galactopyranoside (4-MUG, Sigma) fluorescence assay[14]. aTc: 100 ng/mL, IPTG: 1 mM, Theo: 4 mM. Error bars were standard deviations of three replicates.

Supplementary Figure 13: Scheme of the sRNA-sensing regazyme breakHHRzRAJ12 and its dysfunctional mutant. (**a**) The regazyme was synthesized in the commercial plasmid vector pSynth, and it was directly co-transformed with the pSTC2 plasmid expressing the *cis*-repressed sfGFP reporter in MG1655Z1 cells. (**b**) The gene expression was characterized through using the reporter sfGFP fluorescence for cells co-transformed with the two plasmids. We observe lower activity than for a system expressed from a single plasmid which is consistent to reduced interaction ability between two RNAs expressed from two different plasmids. aTc: 100 ng/mL; IPTG: 1 mM. (**c**) To exclude the possibility that the sRNA *Break1* could directly activate the *cis*-repressed sfGFP, we generated a dysfunctional mutant by removing the regazyme (breakHHRzRAJ12) from the plasmid by PCR-based mutagenesis. The corresponding characterization at population level was shown in **Figure 3b.**

Supplementary Figure 14: Dose-dependent activation of the three theophylline sensing regazymes. The left panel shows the construct that has been used for the characterization, while the right panel shows the GFP expression in MG1655Z1 cells expressing the corresponding plasmid. (**a**) theoHHAzRAJ12; (**b**) theoHHAzRAJ11; (**c**) theoHHAzRR12. Theo: theophylline. Error bars were standard deviations of six replicates.

Supplementary Figure 15: Characterization of regazyme tppHHAzRAJ12. We present the results of GFP expression (population level) of T7 express cells (New England bioLAB, USA) expressing tppHHAzRAJ12. The construction being used for the characterization was shown in the upper panel. T7 express cells (New England BioLAB, USA) transformed with the construct were exposed to different concentration of thiamine as indicated in the figure. Error bars were standard deviation of three replicates.

Supplementary Figure 16: Microfluidics device constructed for this study. (**a**) Scheme of the device used for the single cell dynamic analysis in the left panel. The blue part is the microchamber region. An exemplifying image of the microchamber is shown in the middle panel. The microchamber is about 30 μm x 30 μm width and 1 μm height. Cells are loaded from the cell inlet and trapped in the microchamber. Exemplifying images of cell trapping are shown in the right panel. Images are then analyzed (thresholding and segmentation, see **Supplementary Materials and Methods**). (**b**) Cell images from the bright-field and fluorescence (green and red) channels. Bright-field images can serve for segmentation and tracking. In our experiments, the medium containing the inducer (e.g., theophylline) also contained the red fluorescent dye sulforhodamine B. The dynamics of red fluorescence was therefore the same as the one of the inducer, which serves to account for the diffusion of the molecules in the microchamber. (**c**) Exemplifying images to demonstrate that GFP expression correlates well with the inducer amount into the microchambers. On the top, we show a time-dependent measurement of the red fluorescence intensity in the microchamber. On the bottom, we show cell images of green fluorescence and bright field in the same time scale. Bar: 10 μm.

Supplementary Figure 17: Microfluidics-based single cell analysis of the theophylline-sensing regazyme theoHHAzRAJ12. (**a**) Scheme of the genetic circuit in JS006 cells.

Although the regazyme and mRNA were under the control of inducible promoters (P_{LtetO1} and P_{LlacO1} respectively), in JS006 strain there is no expression of repressors LacI and TetR. Therefore, cells could be induced with theophylline directly. A square wave of theophylline (25 mM) with period T = 8 h (i.e., 4 h induction and 4 h relaxation) was applied. (**b**) Single cell tracking in two different and independent microchambers. Data and plots were generated with MATLAB (MathWorks).

Supplementary Figure 18: Microfluidics-based single cell analysis of the sRNA-sensing regazyme breakHHRzRAJ12. (**a**) Scheme of the genetic circuit in MG1655Z1 cells. A constant amount of IPTG (1 mM) was established during the whole experiment. A square wave of aTc (100 ng/mL) with period T = 8 h (i.e., 4 h induction and 4 h relaxation) was applied. (**b**) Single cell tracking in two different and independent microchambers. Data and plots were generated with MATLAB (MathWorks).

Supplementary Figure 19: Microfluidics-based single cell analysis of the regazyme theoHHAzRAJ11. (**a**) Scheme of the genetic circuit. (**b**) Distributions of fluorescence with time across a population of cells and fitted with a Gaussian model. (**c**) Single cell tracking as done as in **Supplementary Figure 17**. Data and plots were generated with MATLAB (MathWorks, USA). JS006 cells were transformed with the corresponding plasmid and were characterized.in the microfluidics device.

Supplementary Figure 20: Microfluidics-based single cell analysis of the regazyme theoHHAzRR12. (**a**) Scheme of the genetic circuit. (**b**) Distributions of fluorescence with time across a population of cells and fitted with a Gaussian model. (**c**) Single cell tracking as done in **Supplementary Figure 17**. Data and plots were generated with MATLAB

(MathWorks, USA). JS006 cells were transformed with the corresponding plasmid and were characterized in the microfluidics device.

Supplementary Figure 21: Model simulations for different parameter values. (**a**) We show how the dynamic response of the system changes with the self-cleavage rate of the regazyme (λ). (**b**) We show that the dynamic response is not affected by the fraction of regazyme cleaved (α) or the copy number (N). These parameters (in addition to K_{reg}) only affect the level of the steady state. These dynamics collapse all in a single one when using a normalized variable as in panel (**a**). Theophylline is introduced at time 100 min. If not specified, the parameters take the values shown in **Supplementary Table 5**.

Supplementary Figure 22. Orthogonality analysis between regazymes. (**a**) Computational prediction of interaction between the released riboregulator and the 5' UTR (for cognate and non-cognate pairs) [1]. (**b**) Experimental orthogonal analysis between regazymes. JS006 cells were transformed with corresponding constructs for characeterization of the cross-talk between regazymes. Theo : 4mM. Error bars represent standard deviations of three replicates. (**c**) Scheme of the composability of reazymes to implement several circuits.

Supplementary Figure 23: Schemes of expanded application of regazyme-based circuits. We show the implementation of cascades (of small molecule-sensing regazymes coupled with sRNA-sensing regazymes), how to increase the fan-in or fan-out of a regazyme-based system, and also the implementation of feedback and feedforward loops (with sRNA-sensing regazymes).

Supplementary Tables

Supplementary Table 1: Sequences of regazymes theoHHAzRAJ11, theoHHAzRAJ12 and theoHHAzRR12 designed in this work. Here, theoHHAzX (where X = RAJ11, RAJ12 or RR12) is the chimera between the aptazyme theoHHAz [15] and the riboregulator X [1,16], which can initiate protein translation of an mRNA through activating the appropriate 5' UTR [1,16]. The aptazyme sequence is bold-faced with the cleavage site underlined (CC). The theophylline aptamer is shown in yellow. The seed region of the riboregulator is shown in cyan (riboregulators have different seed sequences). The transcription terminator T500 was used in this work [17], and it is shown in magenta (efficiency > 90%).

>theoHHAzRAJ11<br><br>UUCUUUCCCGG**GUACAUCCAGCUGAUGAGUCCCAAAUAGGACGAAAUACAUACCAGCCGAAAGGCCCUUGGCAGG**<br>**UGUCCUGGAUUCCA**CCGGGGAGGGUUGAUUGUGUGAGUCUGUCACAGUUCAGCGGAAACGUUGAUGCUGUGACAG<br>AUUUAUGCGAGGCCAAAGCCCGCCGAAAGGCGGGCUUUUCUGU
>theoHHAzRAJ12<br><br>GGCGCUGCCUUC**GUACAUCCAGCUGAUGAGUCCCAAAUAGGACGAAAUACAUACCAGCCGAAAGGCCCUUGGCAG**<br>**GUGUCCUGGAUUCCA**GACGGGCAGGAAGAAGGGUUCCUUUGAGCGAAUCUAGCGGCACCUCGCUAGGAUUUGCUC<br>GAAGGGAUUCUGGGCAAAGCCCGCCGAAAGGCGGGCUUUUCUGU
>theoHHAzRR12<br><br>UUGGGUA**GUACAUCCAGCUGAUGAGUCCCAAAUAGGACGAAAUACAUACCAGCCGAAAGGCCCUUGGCAGGUGUC**<br>**CUGGAUUCCA**AAACCCAAAUCCAGGAGGUGAUUGGUAGUGGUGGUUAAUGAAAAUUAACUUACUACUACCAUAUA<br>UCUCUAGAAUCAAAGCCCGCCGAAAGGCGGGCUUUUCUGU

Supplementary Table 2: Sequences of regazymes tppHHAzRAJ12 and breakHHRzRAJ12 designed in this work. The tppHHAzRAJ12 is the chimera between the aptazyme tppHHAz [18] and the riboregulator RAJ12 [1]; The breakHHRzRAJ12 is the chimera between a minimal ribozyme [19] and the riboregulator RAJ12. The riboregulator can activate protein translation of an mRNA with the appropriate 5' UTR [1]. The aptazyme/ribozyme sequence is bold-faced, and the cleavage site is underlined (CC or AG). The thiamine pyrophosphate (TPP) aptamer in tppHHAzRAJ12 and the binding sequence to a small RNA (Break1) in breakHHRzRAJ12 are shown in yellow. The seed region of the riboregulator is shown in cyan. The transcription terminator T500 was used in this work [17], and it is shown in magenta (efficiency > 90%).

>tppHHAzRAJ12<br><br>GGCGCUGCCUUC**GUACAUCCAGCUGAUGAGUCCCAAAUAGGACGAAA*****ACA*****UCGGGGUGCCCUUCUGCGUGAAGGC**<br>**UGAGAAAUACCCGUAUCACCUGAUCUGGAUAAUGCCAGCGUAGGGAU*****UAU*****UCCUGGAUUCCA**GACGGGCAGGAAG<br>AAGGGUUCCUUUGAGCGAAUCUAGCGGCACCUCGCUAGGAUUUGCUCGAAGGGAUUCUGGGCAAAGCCCGCCGAA<br>AGGCGGGCUUUUCUGU
>breakHHRzRAJ12<br><br>GGCGCTGCCTTC**GGGCGACCCTGATGAGCTTGAGTTTAGCTCGTCACTGTCCAGGTTCAATCAGGCGAAACGGTG**<br>**AAAGCCGTAGGTTGCCC**GACGGGCAGGAAGAAGGGTTCCTTTGAGCGAATCTAGCGGCACCTCGCTAGGATTTGC<br>TCGAAGGGATTCTGGGCAAAGCCCGCCGAAAGGCGGGCTTTTCTGT
>Break1<br><br>GGGAAACCCUGAACCUGGACAGUGACGAGCUAUCCUUAGCGAAAGCUAAGGAUUUUUUUU

Supplementary Table 3: Sequences of dysfunctional mutant regazymes. The theophylline aptamer is shown in yellow (see details in **Supplementary Table 1**). The mutations U23C and A27C in the aptamer (local numbering) [20], which destroy the binding to the ligand, are shown in red. The mutation U to G that inactivates the apatzyme is also shown in red, as well as the mutation A to G in the core catalytic domain destroying the activity.

>theoHHAzRAJ11mut<br><br>UUCUUUCCCGG**GUACAUCCAGCUGAUGAGUCCCAAAUAGGACGAAAUACAUACCAGCCGAAAGGCCCUCGGCCGG**<br>**UGUCCUGGAUUCCA**CCGGGGAGGGUUGAUUGUGUGAGUCUGUCACAGUUCAGCGGAAACGUUGAUGCUGUGACAG<br>AUUUAUGCGAGGCCAAAGCCCGCCGAAAGGCGGGCUUUUCUGU
>theoHHAzRAJ12mut<br><br>GGCGCUGCCUUC**GUACAUCCAGCUGAUGAGUCCCAAAUAGGACGAAAUACAUACCAGCCGAAAGGCCCUCGGCCG**<br>**GUGUCCUGGAUUCCA**GACGGGCAGGAAGAAGGGUUCCUUUGAGCGAAUCUAGCGGCACCUCGCUAGGAUUUGCUC<br>GAAGGGAUUCUGGGCAAAGCCCGCCGAAAGGCGGGCUUUUCUGU
>theoHHAzRAJ12Cm<br><br>GGCGCUGCCUUC**GUACAUCCAGCUGAUGAGUCCCAAAUAGGACGAAAGACAUACCAGCCGAAAGGCCCUUGGCAG**<br>**GUGUCCUGGAUUCCA**GACGGGCAGGAAGAAGGGUUCCUUUGAGCGAAUCUAGCGGCACCUCGCUAGGAUUUGCUC<br>GAAGGGAUUCUGGGCAAAGCCCGCCGAAAGGCGGGCUUUUCUGU
>theoHHAzRR12mut<br><br>UUGGGUA**GUACAUCCAGCUGAUGAGUCCCAAAUAGGACGAAAUACAUACCAGCCGAAAGGCCCUCGGCCGGUGUC**<br>**CUGGAUUCCA**AAACCCAAAUCCAGGAGGUGAUUGGUAGUGGUGGUUAAUGAAAAUUAACUUACUACUACCAUAUA<br>UCUCUAGAAUCAAAGCCCGCCGAAAGGCGGGCUUUUCUGU
>theoHHAzRR12Cm<br><br>UUGGGUA**GUACAUCCAGCUGAUGAGUCCCAAAUAGGACGAAAGACAUACCAGCCGAAAGGCCCUUGGCAGGUGUC**<br>**CUGGAUUCCA**AAACCCAAAUCCAGGAGGUGAUUGGUAGUGGUGGUUAAUGAAAAUUAACUUACUACUACCAUAUA<br>UCUCUAGAAUCAAAGCCCGCCGAAAGGCGGGCUUUUCUGU
>theoHHAzRAJ12AGm<br><br>GGCGCUGCCUUC**GUACAUCCAGCUGAUGAGUCCCAAAUAGGACGAGAUACAUACCAGCCGAAAGGCCCUUGGCAG**<br>**GUGUCCUGGAUUCCA**GACGGGCAGGAAGAAGGGUUCCUUUGAGCGAAUCUAGCGGCACCUCGCUAGGAUUUGCUC<br>GAAGGGAUUCUGGGCAAAGCCCGCCGAAAGGCGGGCUUUUCUGU

Supplementary Table 4: Sequences and structures of the 5' UTRs. The *cis*-repressed 5' UTR sequences of the mRNA reporter and the corresponding active complex (5' UTR + riboregulator) are shown with RBS (Shine-Dalgarno sequence) marked as yellow. The corresponding secondary structures are shown, where RBS is exposed for ribosome binding upon interaction between riboregulator and 5' UTR [1,16]. The start codon is shown in green.

```
>cisRAJ11

CCUCGCAUAAUUUCACUUCUUCAAUCCUCCCGUUAAAGAGGAGAAAUUAUGAAUG
......((((((((.((((((.(((......))).)))))).)))))))).....
```

```
>cisRAJ12

ACCCAGUAUCAUUCUCUUCUUCCUGCCCACGCGGAAAGAGGAGAAAGGUGUAAUG
......((((.((((((((((.(((......))).)))))))))).)))).....
```

```
>cisRR12

CAAUUCUACCAUUCACCUCUUGGAUUUGGGUAUUAAAGAGGAGAAAGGUACCAUG
......((((.(((.((((((.(((......))).)))))).))).)))).....
```

```
>cisRAJ11+transRAJ11

CCUCGCAUAAUUUCACUUCUUCAAUCCUCCCGUUAAAGAGGAGAAAUUAUGAAUG +
((((((((((..((((....(((((((((((........................ +

GGGAGGGUUGAUUGUGUGAGUCUGUCACAGUUCAGCGGAAACGUUGAUGCUGUGACAGAUUUAUGCGAGGC
)))))))))))....)))).(((((((((((((((((....)))))).))))))))))).)))))))))).
```

```
>cisRAJ12+transRAJ12

ACCCAGUAUCAUUCUCUUCUUCCUGCCCACGCGGAAAGAGGAGAAAGGUGUAAUG +
.(((((.(((...(((((((((((((((........................... +

GGGCAGGAAGAAGGGUUCCUUUGAGCGAAUCUAGCGGCACCUCGCUAGGAUUUGCUCGAAGGGAUUCUGGG
)))))))))))))))..((((((((((((((((((((....)))))).))))))))))))))))).)))))
```

```
>cisRR12+transRR12

CAAUUCUACCAUUCACCUCUUGGAUUUGGGUAUUAAAGAGGAGAAAGGUACCAUG +
....((((....(((((((((((((((((((........................ +

ACCCAAAUCCAGGAGGUGAUUGGUAGUGGUGGUUAAUGAAAAUUAACUUACUACUACCAUAUAUCUCUAGA
))))))))))))))))))).(((((((((((((((((....)))))).)))))))))))........))))
```

Supplementary Table 5: Parameter values for the mathematical model.

Parameter	Value	Parameter	Value
Π_m	11 nM/min [5,21]	K_{theo}	1 mM [b]
Π_r	11 nM/min [5,21]	α_0	0.2 [b]
K_i	15 μM [1]	α	0.8 [b]
K_a	3 ng/mL [1]	K_{reg}	1000 nM [a]
n_i	2 [a]	β_0	0 nM/min [1]
n_a	2 [a]	β	0.1 nM/min [1]
f_i	600 [5]	δ_g	0.08 min^{-1} [b, c]
f_a	2500 [5]	δ_m	0.35 min^{-1} [a]
N	100 copies [a]	δ_r	0.35 min^{-1} [a]
λ	0.15 min^{-1} [b]	μ	0.005 – 0.01 min^{-1} [b]

[a] Value assumed from this work.

[b] Value estimated from data obtained in this work.

[c] This value corresponds to a protein with LAA degradation tag. According to Wong et al. [4], $\delta_g = 0.04\ \mathrm{min}^{-1}$, but with our data we obtained a higher value. Without tag, it could be neglected ($\delta_g = 0$).

Supplementary Table 6: Strains and plasmids used in this work.

**Strains or plasmids**	**Features**	**Ref.**
E. coli DH5α	Commercial	Invitrogen
E. coli DH5αZ1	Commercial (DH5α, *lacI*Q, PN25-*tetR*, SpR)	Clontech
E. coli JS006	K-12 MG1655, Δ*araC*, Δ*lacI*	Stricker et al [7]
E. coli MG1655Z1	K-12 MG1655, *lacI*Q, PN25-*tetR*, SpR	Cox et al [8]
E. coli T7 Express	Commercial (#C3009I)	NEB
pSynth	Commercial plasmid pIDTSMART, pUC ori, AmpR	IDT
pSTC1	pSC101m ori, KanR	Rodrigo et al [1]
pSTC2	pSC101m ori, KanR	This work
pSCKtheoRAJ11	pSTC1, theoHHAzRAJ11, cisRAJ11-sfGFP	This work
pSCKtheoRAJ11m	pSTC1, theoHHAzRAJ11mut, cisRAJ11-sfGFP	This work
pSCKtheoRAJ12	pSTC2, theoHHAzRAJ12, cisRAJ12-sfGFP	This work
pSCKtheoRAJ12m	pSTC2, theoHHAzRAJ12mut, cisRAJ12-sfGFP	This work
pSCKtheoRAJ12Cm	pSTC2, theoHHAzRAJ12Cm, cisRAJ12-sfGFP	This work
pSCKtheoRAJ12AGm	pSTC2, theoHHAzRAJ12AGm, cisRAJ12-sfGFP	This work
pSCKtheoRR12	pSTC2, theoHHAzRR12, cisRR12-sfGFP	This work
pSCKtheoRR12m	pSTC2, theoHHAzRR12mut, cisRR12-sfGFP	This work
pSCKtheoRR12Cm	pSTC2, theoHHAzRR12Cm, cisRR12-sfGFP	This work
pSCKtppRAJ12	pSTC2, tppHHAzRR12, cisRAJ12-sfGFP	This work
pUAbreak12	pIDTSMART, breakHHRzRAJ12, Break1	This work
pUAbreak12m	pIDTSMART, Break1 only	This work
pSCKbreak12	pSTC2, cisRAJ12-sfGFP	This work
pSCKtheoRAJ11-o12	pSTC2, theoHHAzRAJ11, cisRAJ12-sfGFP	This work
pSCKtheoRAJ11-oIsa	pSTC2, theoHHAzRAJ11, cisRR12-sfGFP	This work
pSCKtheoRAJ12-o11	pSTC1, theoHHAzRAJ12, cisRAJ11-sfGFP	This work
pSCKtheoRAJ12-oIsa	pSTC2, theoHHAzRAJ12, cisRR12-sfGFP	This work
pSCKtheoRR12-o11	pSTC2, theoHHAzRR12, cisRAJ11-sfGFP	This work
pSCKtheoRR12-o12	pSTC2, theoHHAzRR12, cisRAJ12-sfGFP	This work

pUAtheoRAJ12	pIDTSMART, theoHHAzRAJ12, cisRAJ12-LacZα	This work

Supplementary References

1. Rodrigo, G., Landrain, T.E. & Jaramillo, A. De novo automated design of small RNA circuits for engineering synthetic riboregulation in living cells. *Proc Natl Acad Sci U S A* **109**, 15271-6 (2012).
2. Pedelacq, J.D., Cabantous, S., Tran, T., Terwilliger, T.C. & Waldo, G.S. Engineering and characterization of a superfolder green fluorescent protein. *Nat Biotechnol* **24**, 79-88 (2006).
3. Hersch, G.L., Baker, T.A. & Sauer, R.T. SspB delivery of substrates for ClpXP proteolysis probed by the design of improved degradation tags. *Proc Natl Acad Sci U S A* **101**, 12136-41 (2004).
4. Wong, W.W., Tsai, T.Y. & Liao, J.C. Single-cell zeroth-order protein degradation enhances the robustness of synthetic oscillator. *Mol Syst Biol* **3**, 130 (2007).
5. Lutz, R. & Bujard, H. Independent and tight regulation of transcriptional units in Escherichia coli via the LacR/O, the TetR/O and AraC/I1-I2 regulatory elements. *Nucleic Acids Res* **25**, 1203-10 (1997).
6. Sambrook, J., Fritsch, E.F. & Maniatis, T. Molecular Cloning: A Laboratory Manual (Cold Spring Harbor Laboratory Press, New York, 1989).
7. Stricker, J. et al. A fast, robust and tunable synthetic gene oscillator. *Nature* **456**, 516-9 (2008).
8. Cox, R.S., 3rd, Surette, M.G. & Elowitz, M.B. Programming gene expression with combinatorial promoters. *Mol Syst Biol* **3**, 145 (2007).
9. Danino, T., Mondragon-Palomino, O., Tsimring, L. & Hasty, J. A synchronized quorum of genetic clocks. *Nature* **463**, 326-30 (2010).
10. Kirkpatrick, S., Gelatt, C.D., Jr. & Vecchi, M.P. Optimization by simulated annealing. *Science* **220**, 671-80 (1983).

11. Hofacker, I.L., Fontana, W., Stadler, P.F., Bonhoeffer, L.S., Tacker, M., S & Schuster, P. . Fast folding and comparison of RNA secondary structures. . *Monatshefte für Chemie* **125**, 167-188 (1994).

12. Zadeh, J.N. et al. NUPACK: Analysis and design of nucleic acid systems. *J Comput Chem* **32**, 170-3 (2011).

13. Darty, K., Denise, A. & Ponty, Y. VARNA: Interactive drawing and editing of the RNA secondary structure. *Bioinformatics* **25**, 1974-5 (2009).

14. Vidal-Aroca, F. et al. One-step high-throughput assay for quantitative detection of beta-galactosidase activity in intact gram-negative bacteria, yeast, and mammalian cells. *Biotechniques* **40**, 433-4, 436, 438 passim (2006).

15. Wieland, M. & Hartig, J.S. Improved aptazyme design and in vivo screening enable riboswitching in bacteria. *Angew Chem Int Ed Engl* **47**, 2604-7 (2008).

16. Isaacs, F.J. et al. Engineered riboregulators enable post-transcriptional control of gene expression. *Nat Biotechnol* **22**, 841-7 (2004).

17. Larson, M.H., Greenleaf, W.J., Landick, R. & Block, S.M. Applied force reveals mechanistic and energetic details of transcription termination. *Cell* **132**, 971-82 (2008).

18. Wieland, M., Benz, A., Klauser, B. & Hartig, J.S. Artificial ribozyme switches containing natural riboswitch aptamer domains. *Angew Chem Int Ed Engl* **48**, 2715-8 (2009).

19. Penchovsky, R. & Breaker, R.R. Computational design and experimental validation of oligonucleotide-sensing allosteric ribozymes. *Nat Biotechnol* **23**, 1424-33 (2005).

20. Zimmermann, G.R., Wick, C.L., Shields, T.P., Jenison, R.D. & Pardi, A. Molecular interactions and metal binding in the theophylline-binding core of an RNA aptamer. *RNA* **6**, 659-67 (2000).

21. Rodrigo, G. & Jaramillo, A. AutoBioCAD: full biodesign automation of genetic circuits. *ACS Synth Biol* **2**, 230-6 (2013).

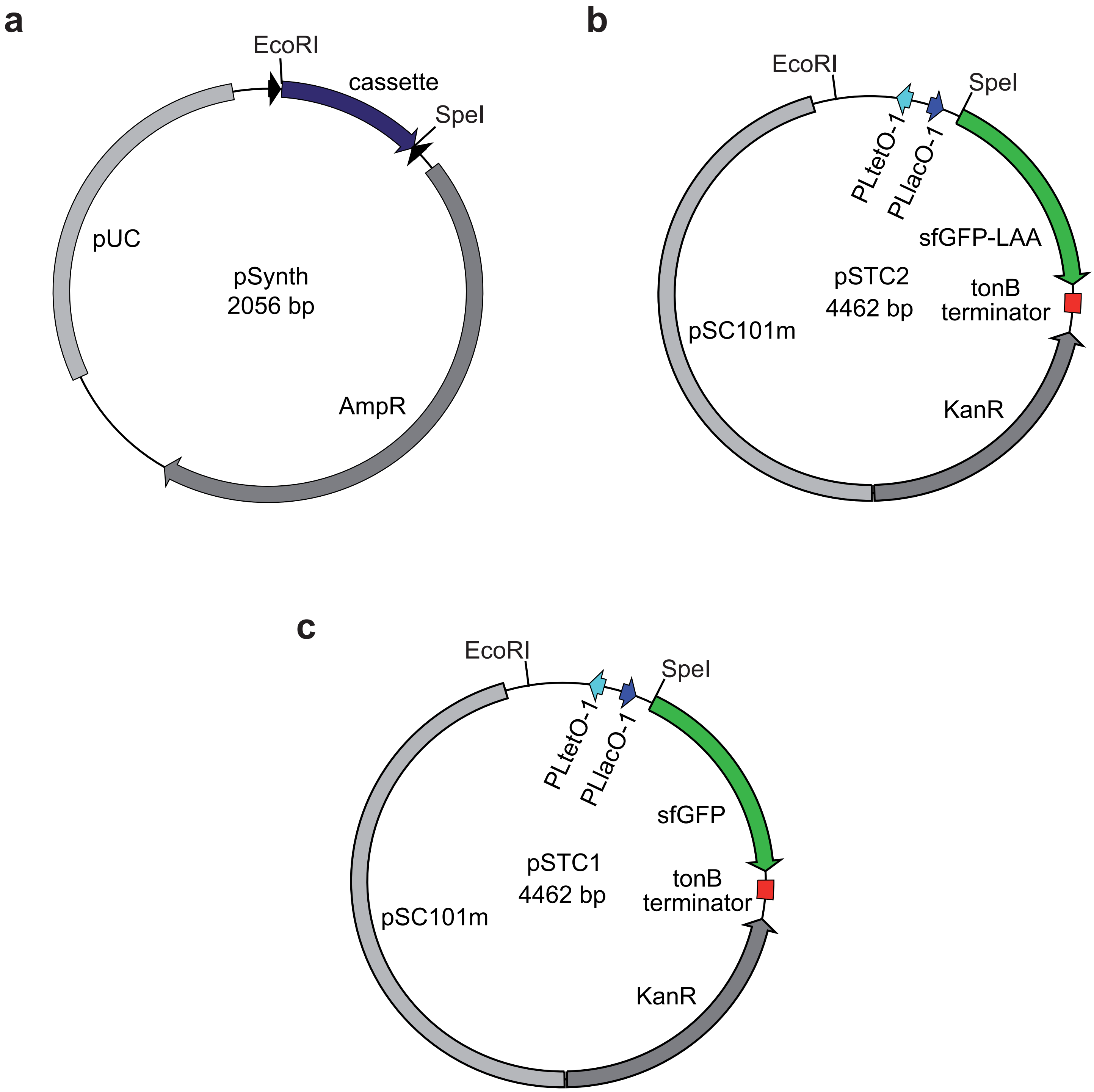

Supplementary Figure 1

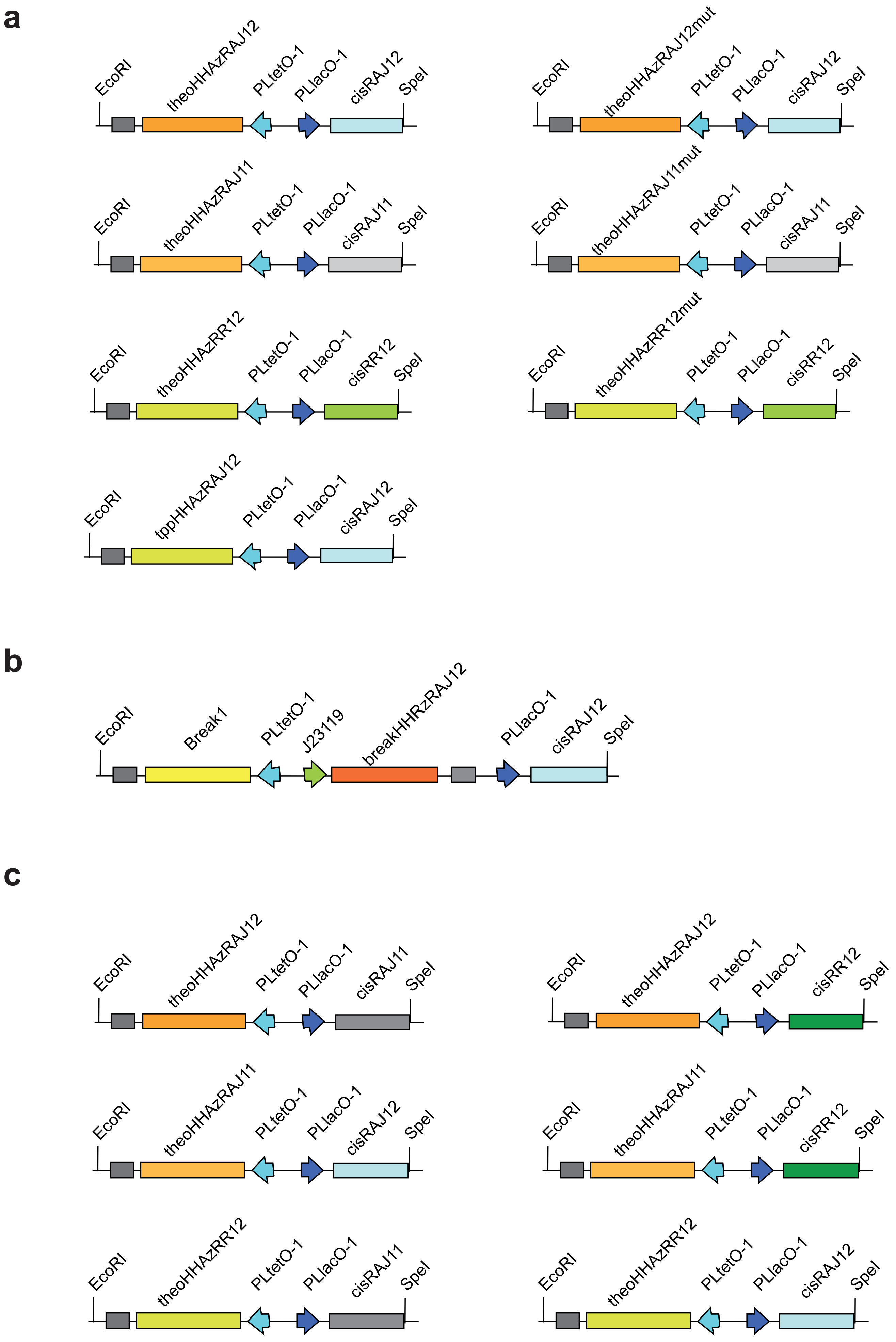

Supplementary Figure 2

a

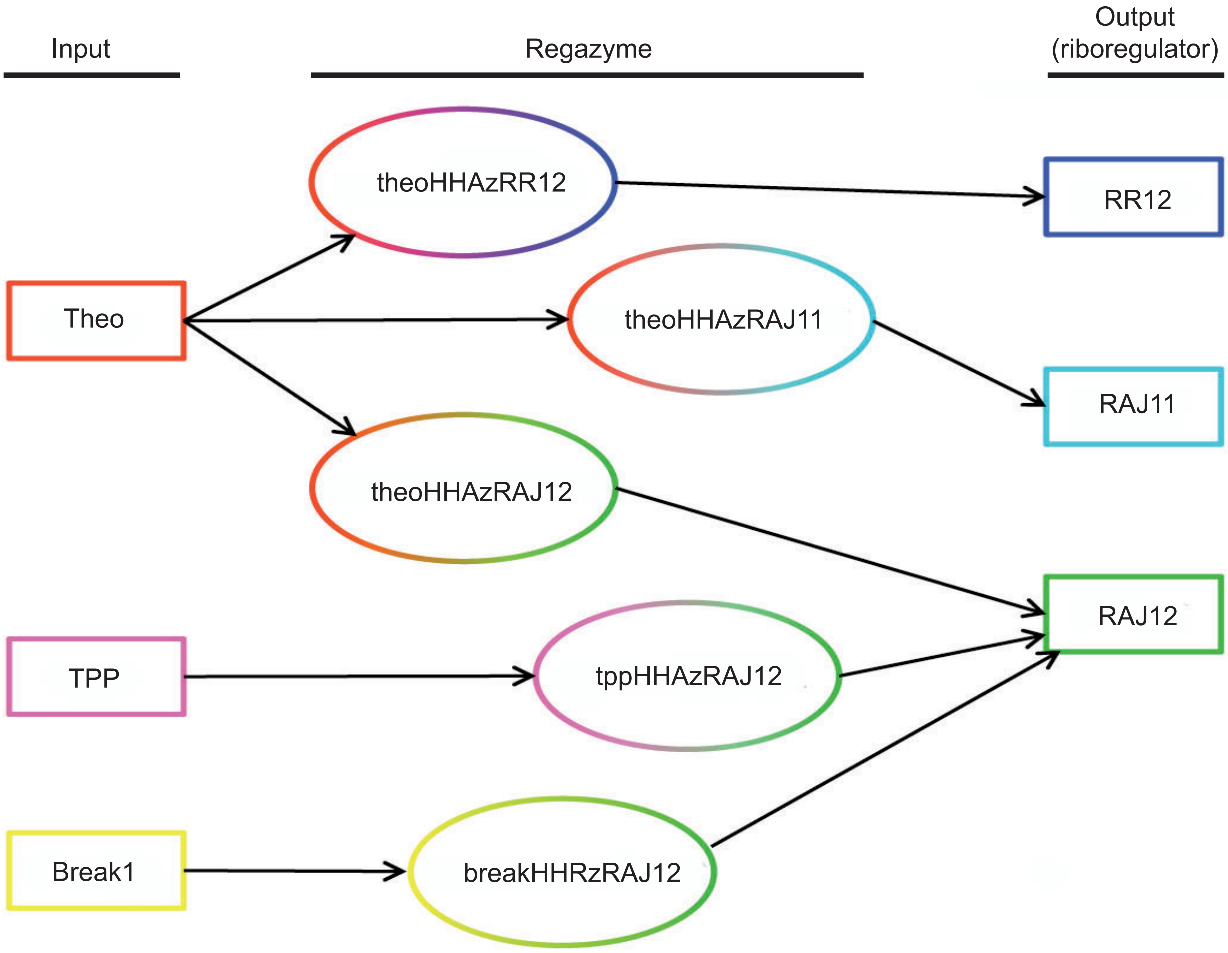

b

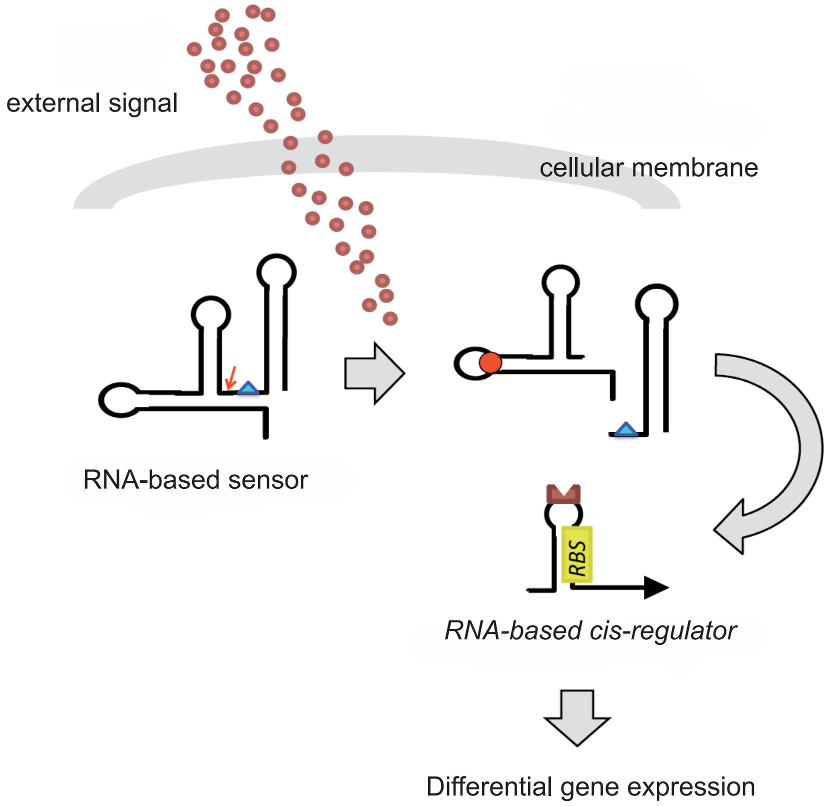

Supplementary Figure 3

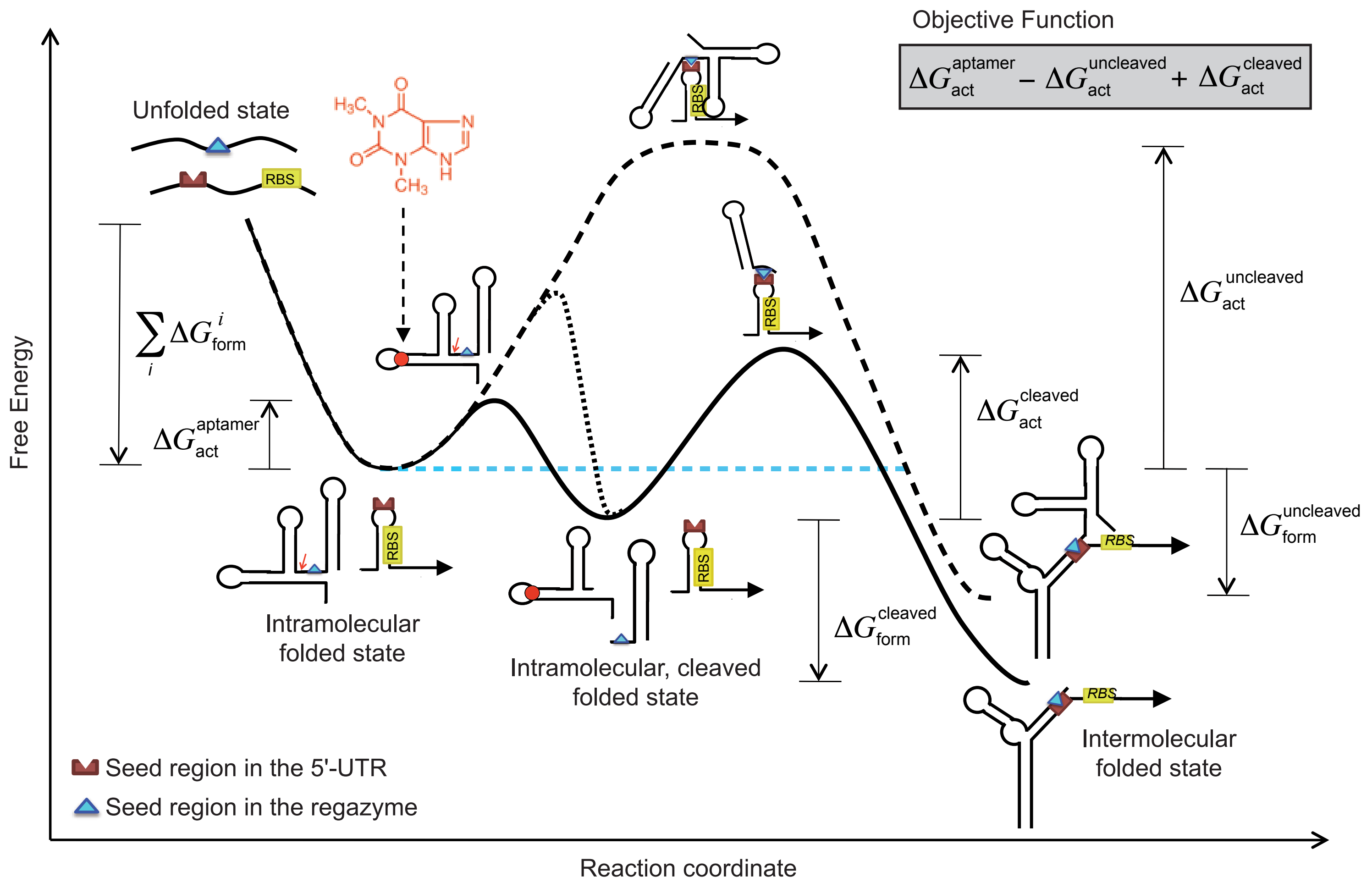

Supplementary Figure 4

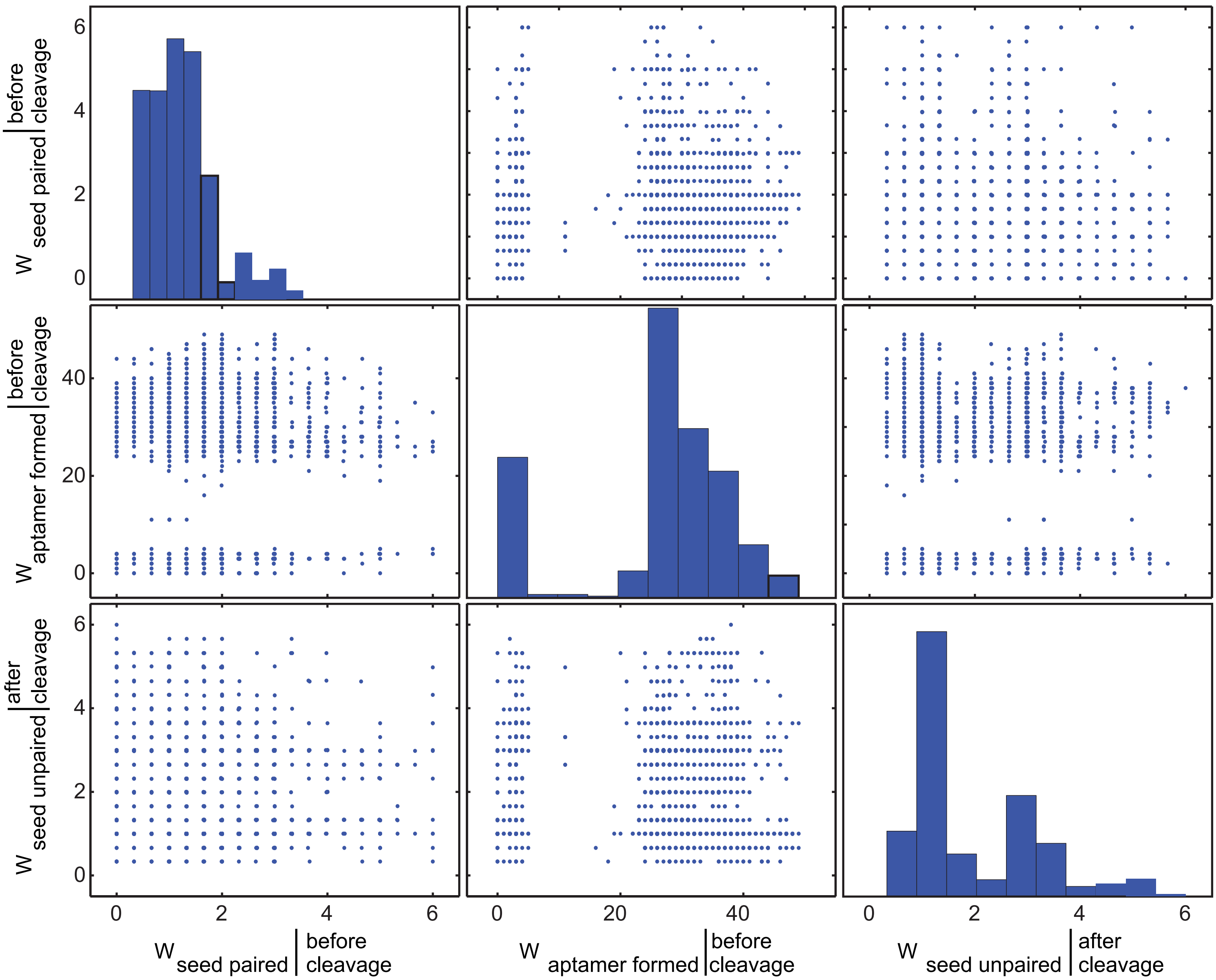

Supplementary Figure 5

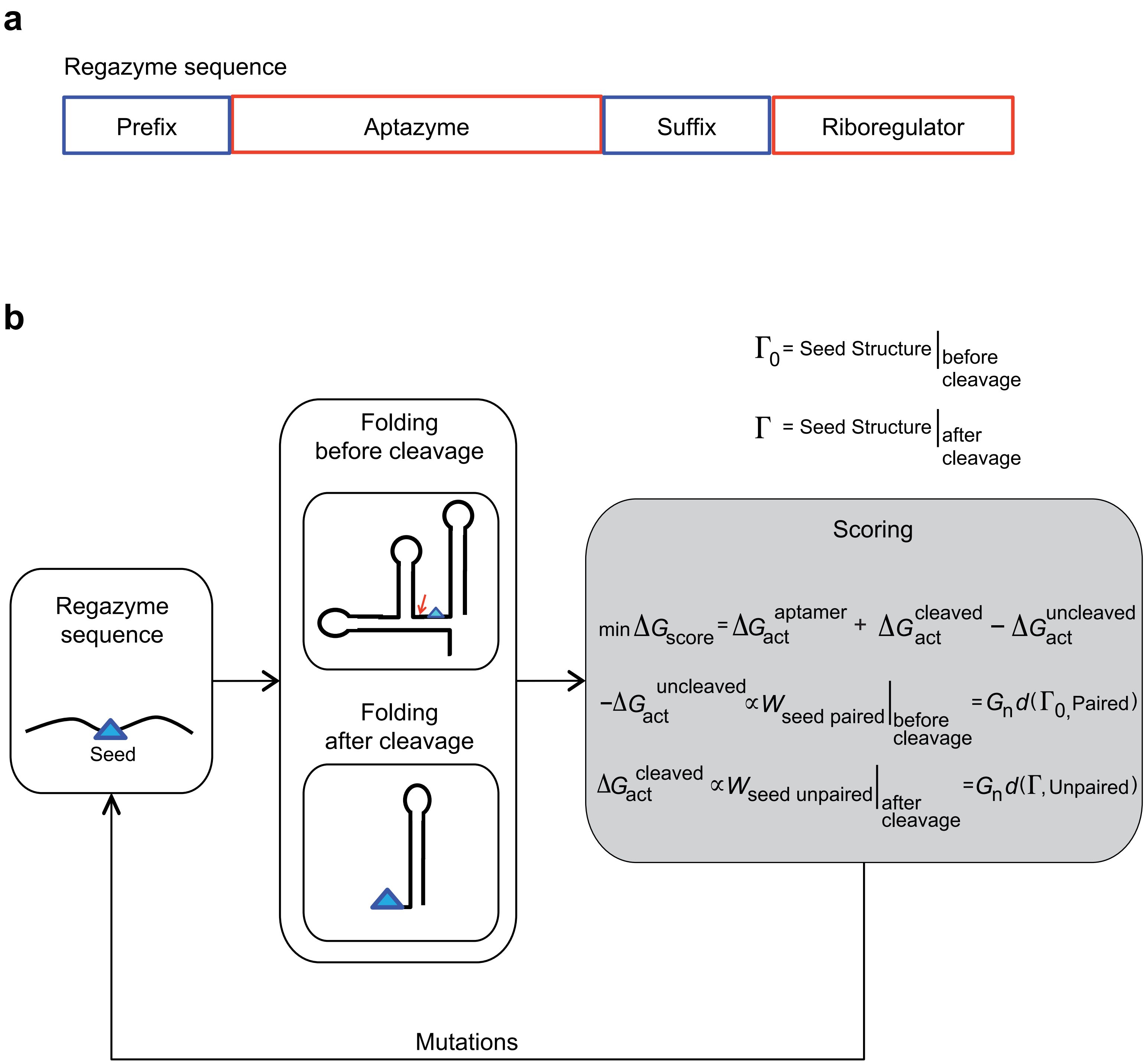

Supplementary Figure 6

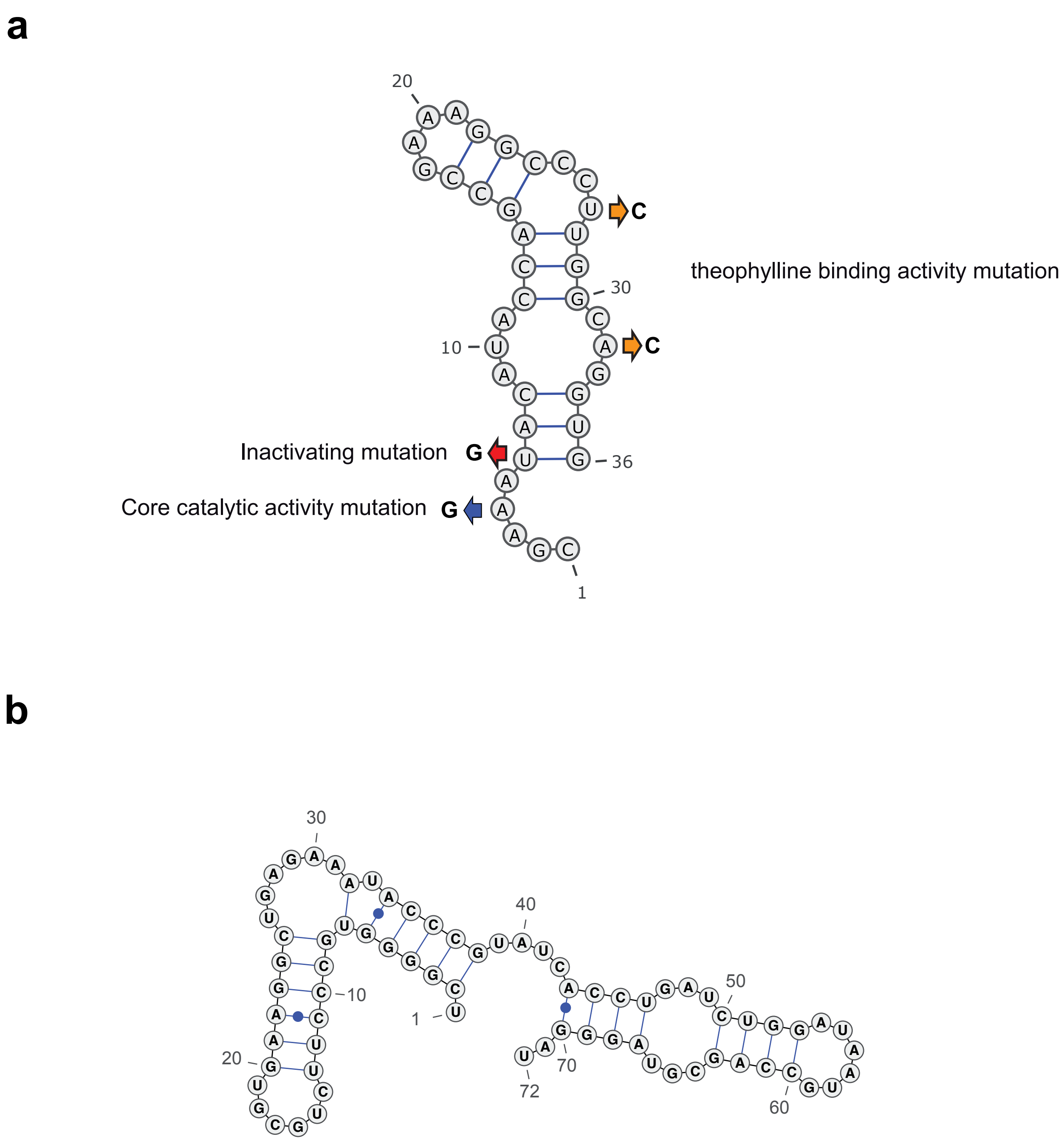

Supplementary Figure 7

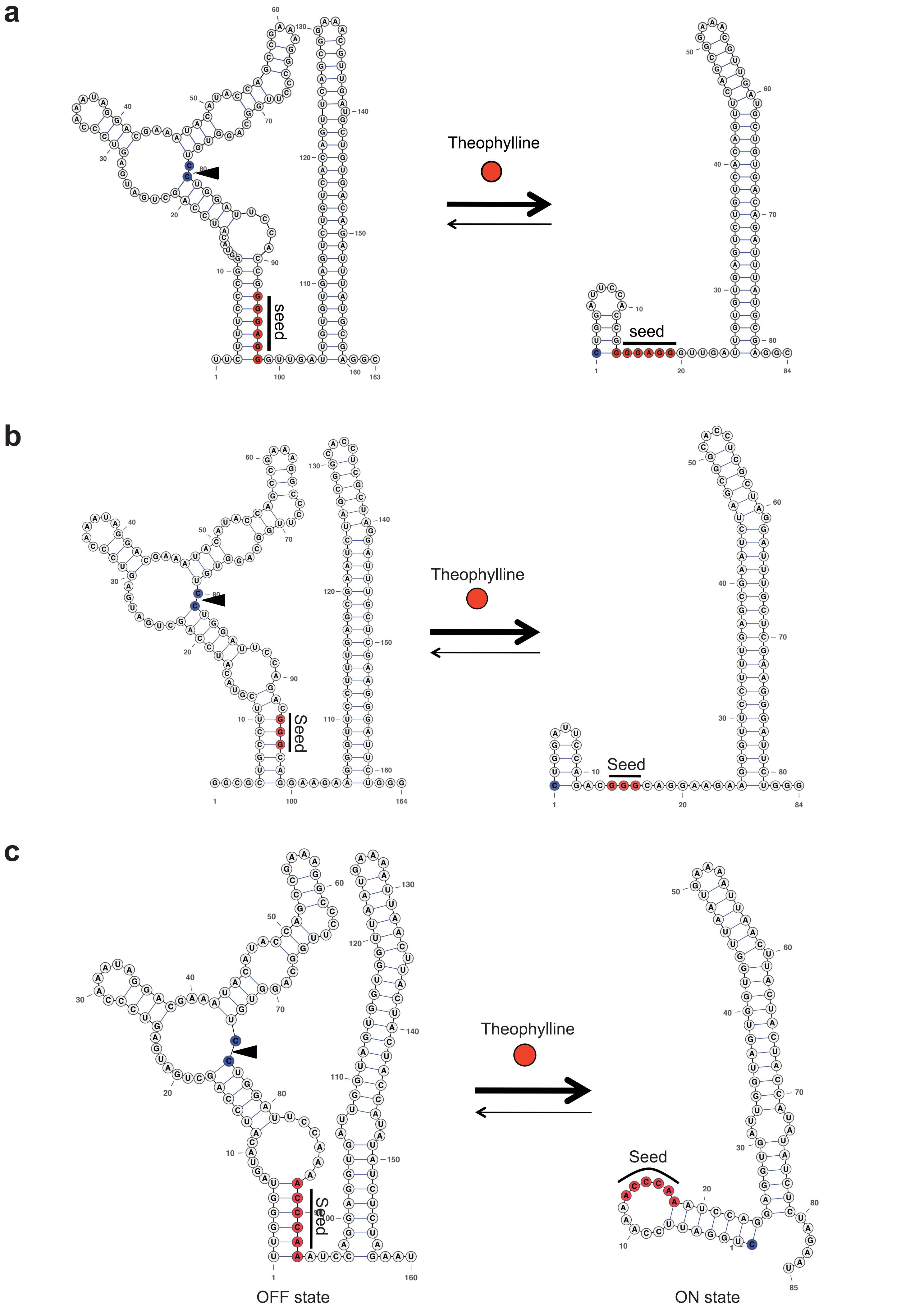

Suppelmentary Figure 8

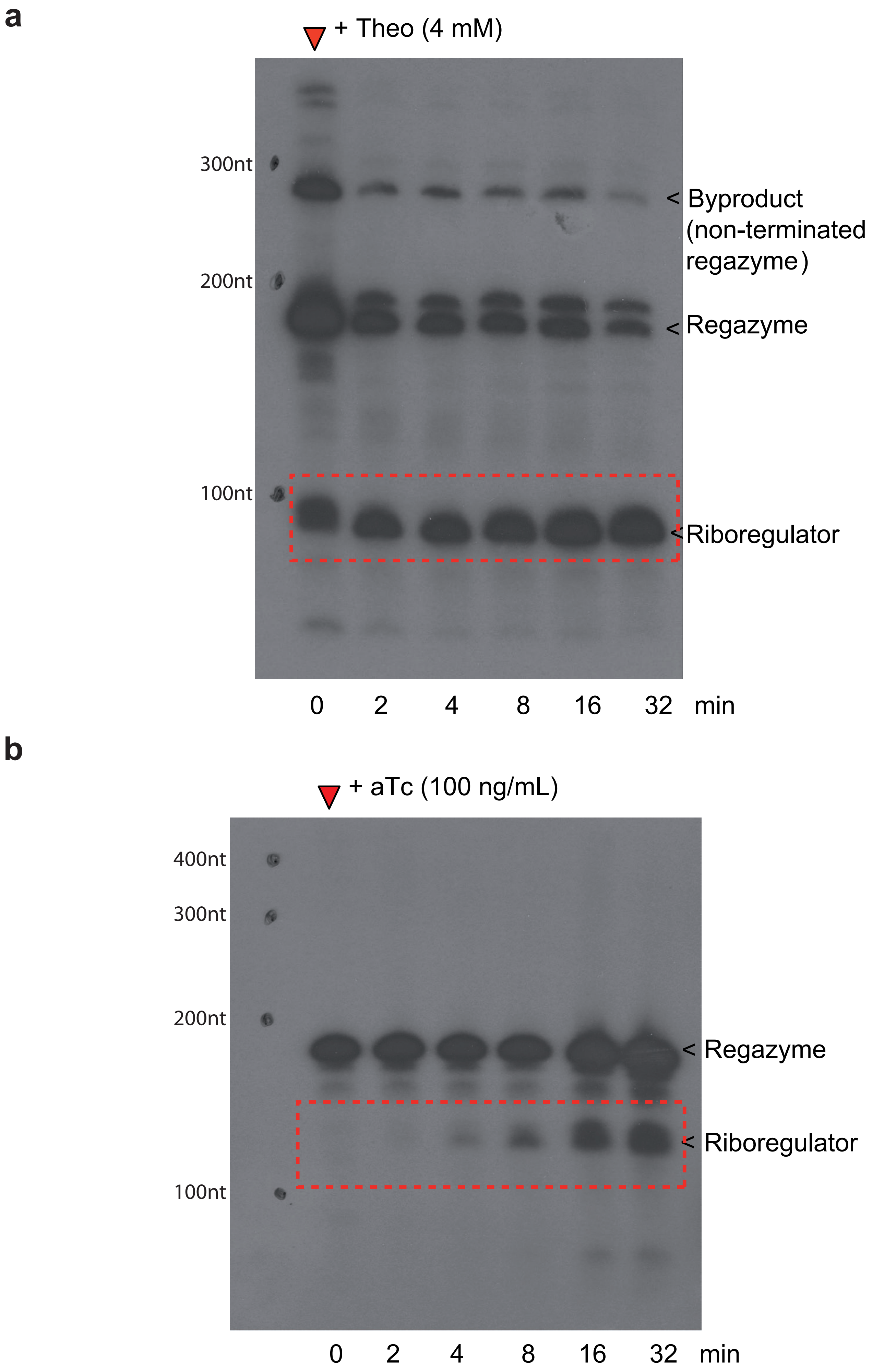

Supplementary Figure 9

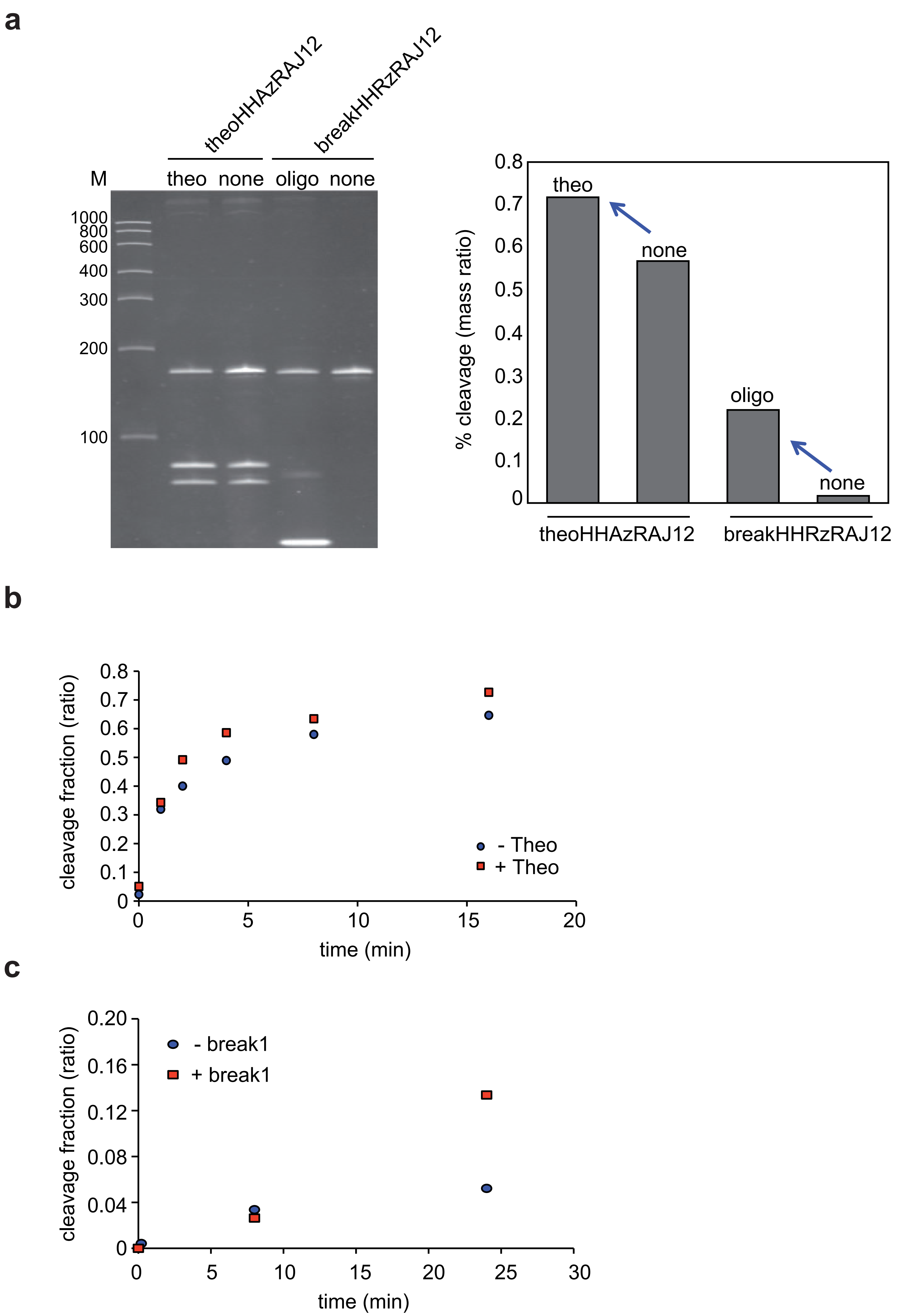

Supplementary Figure 10

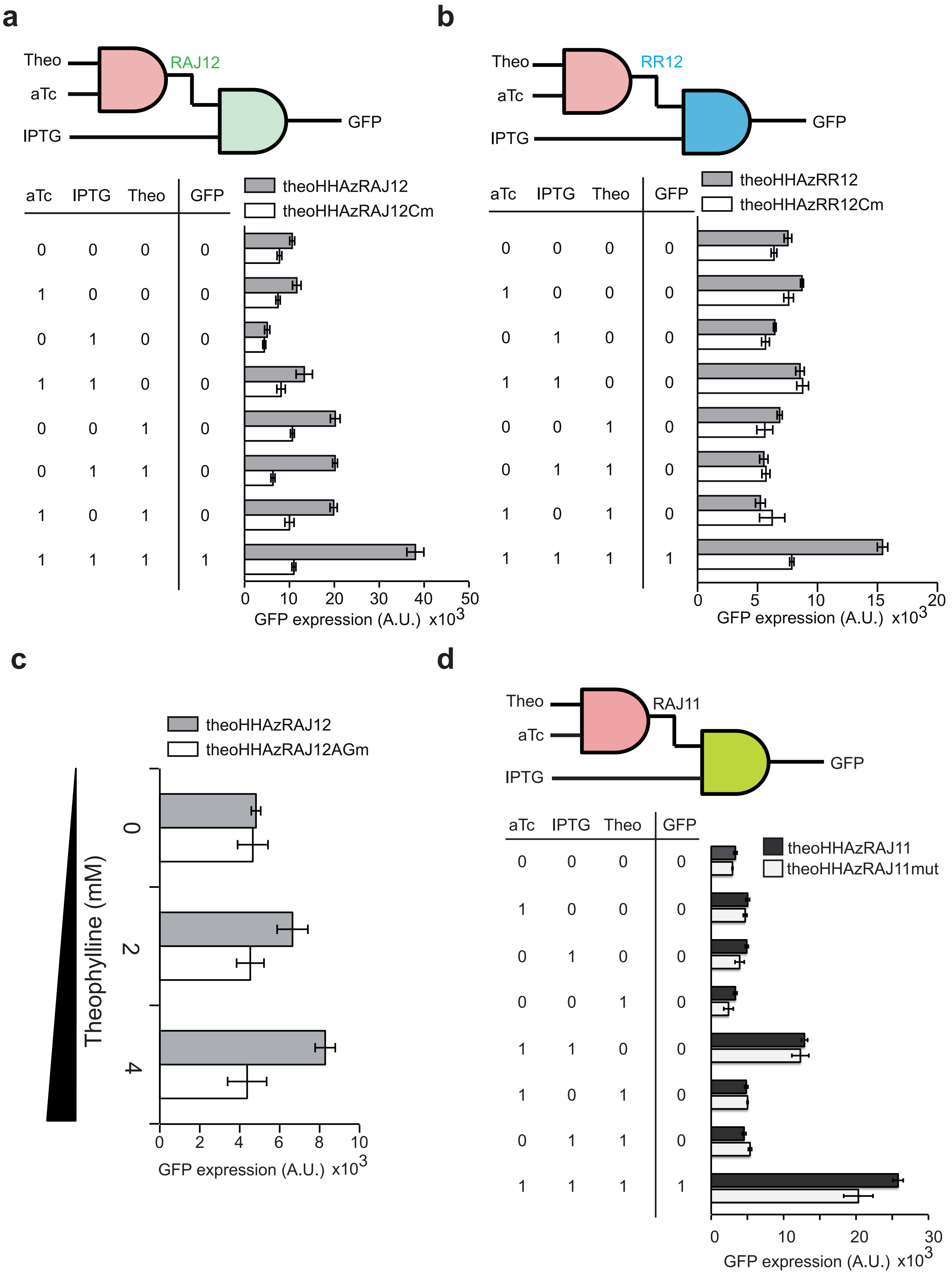

Supplementary Figure 11

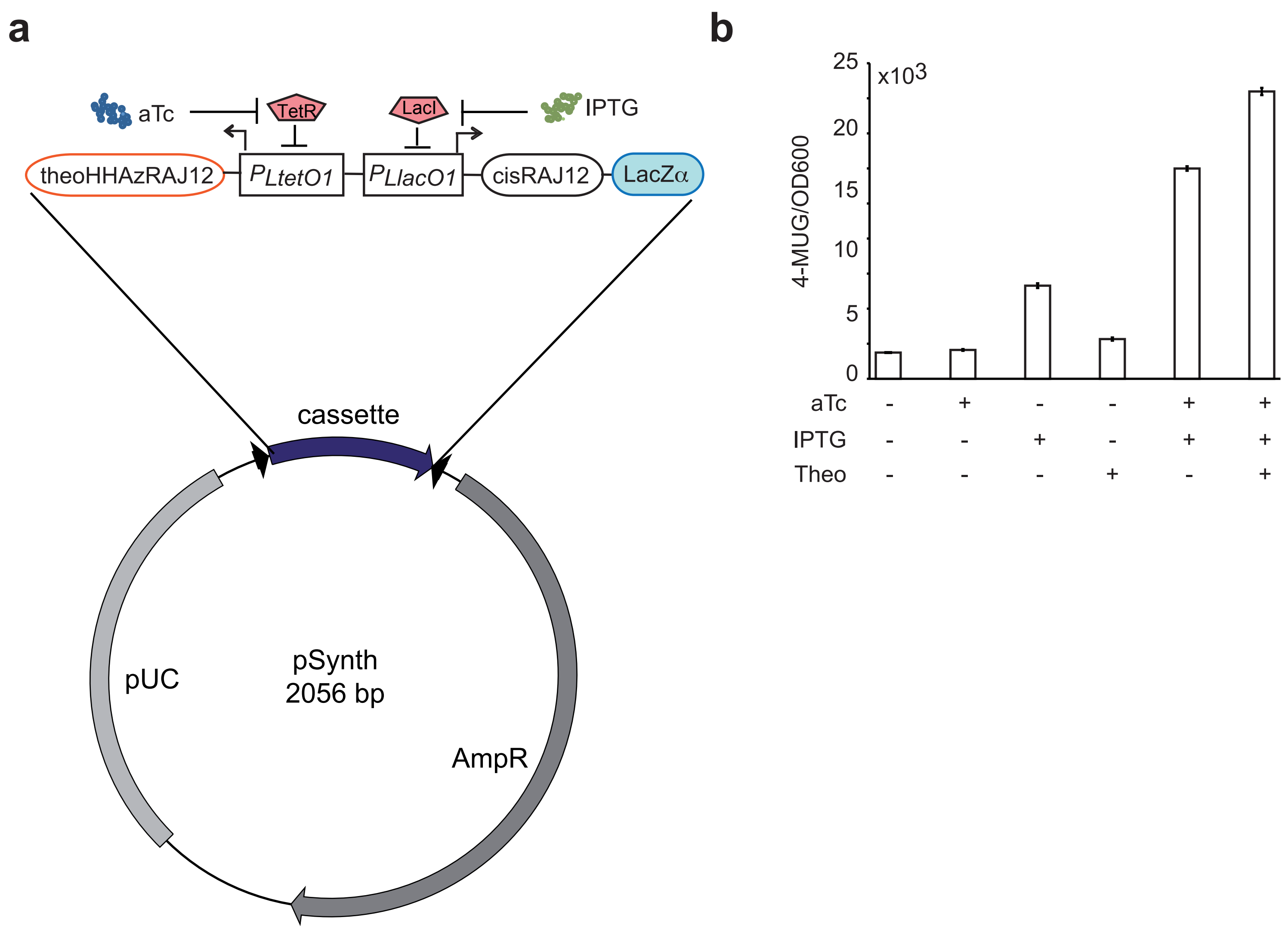

Supplementary Figure 12

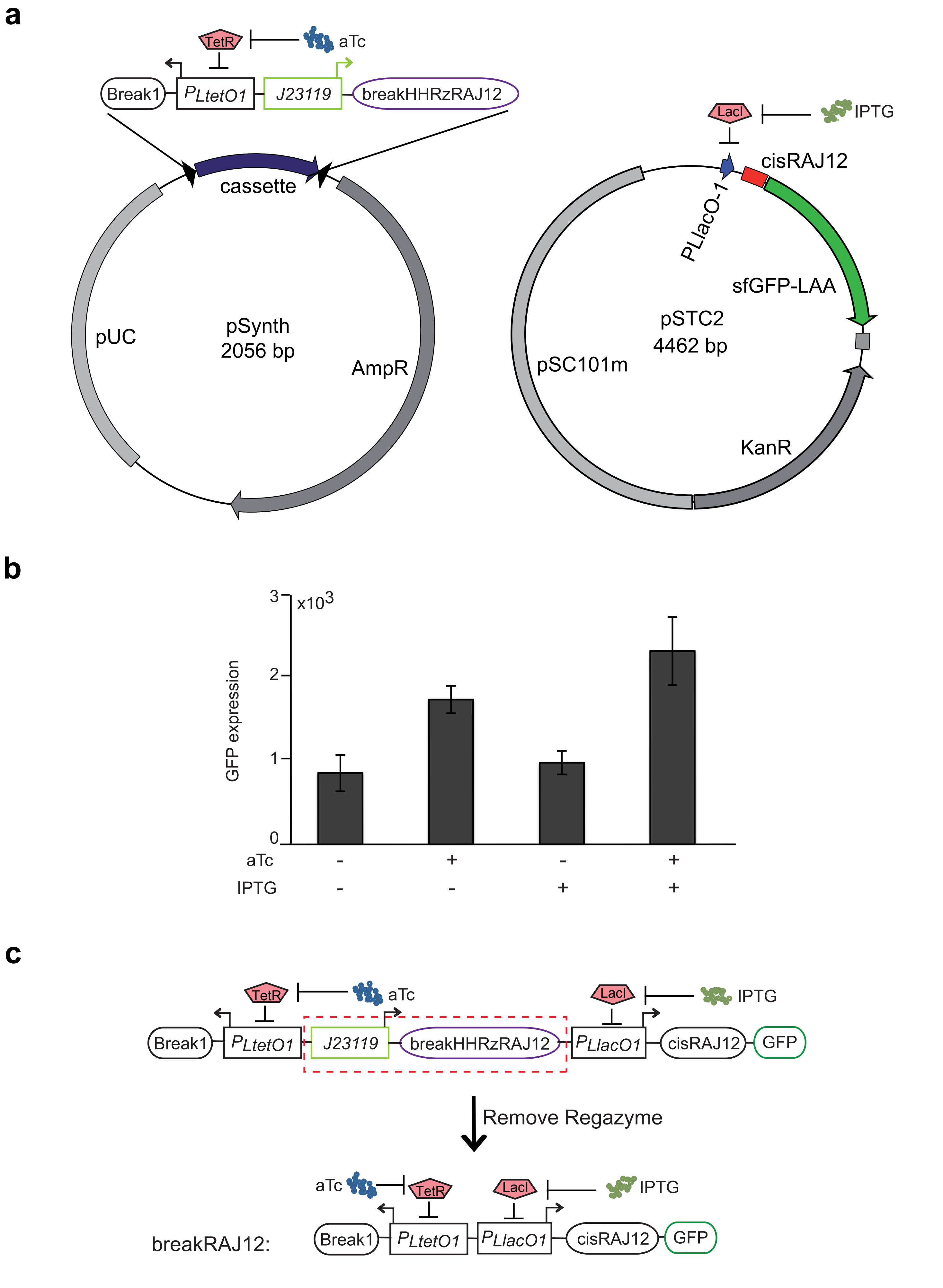

Supplementary Figure 13

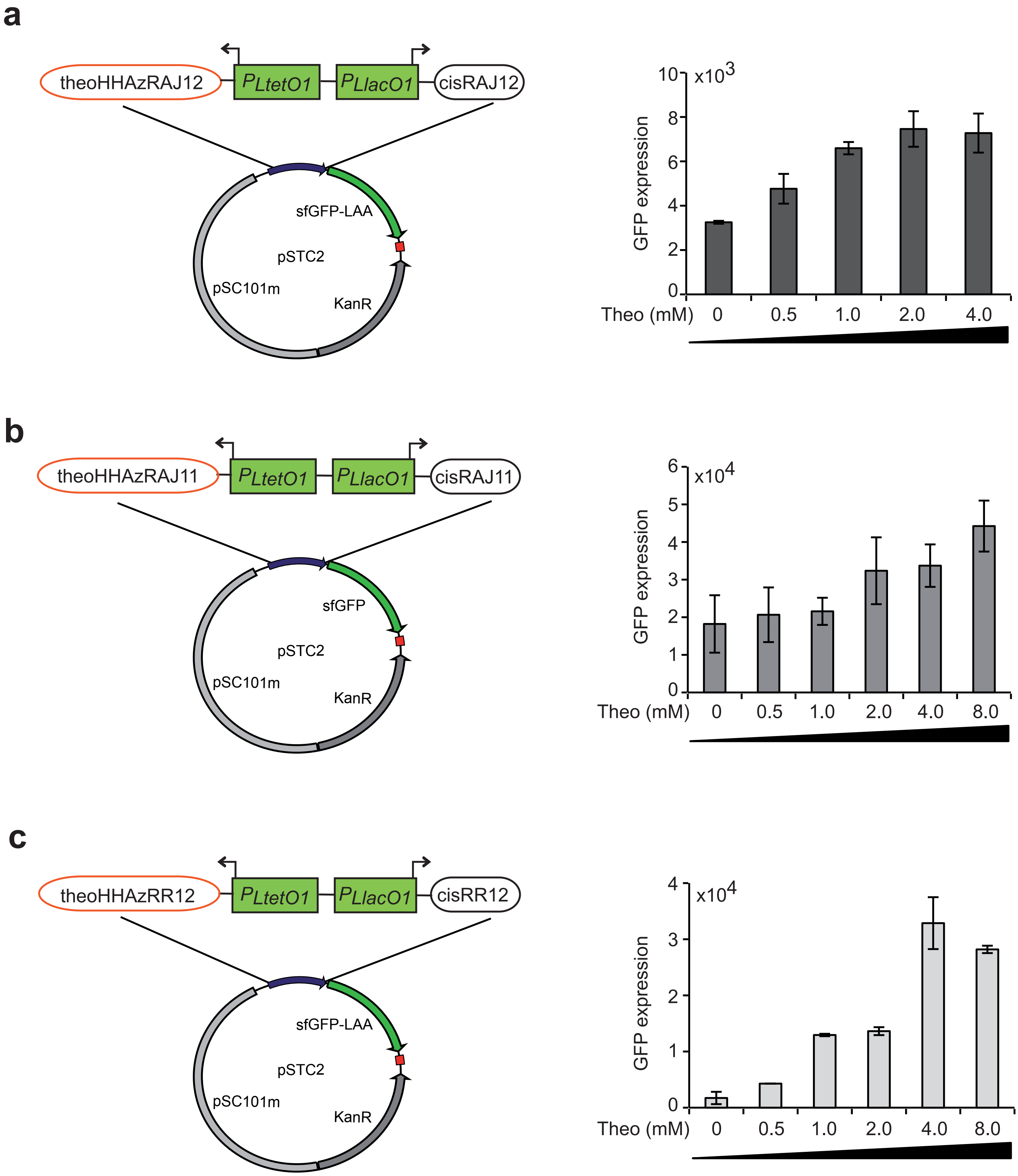

Supplementary Figure 14

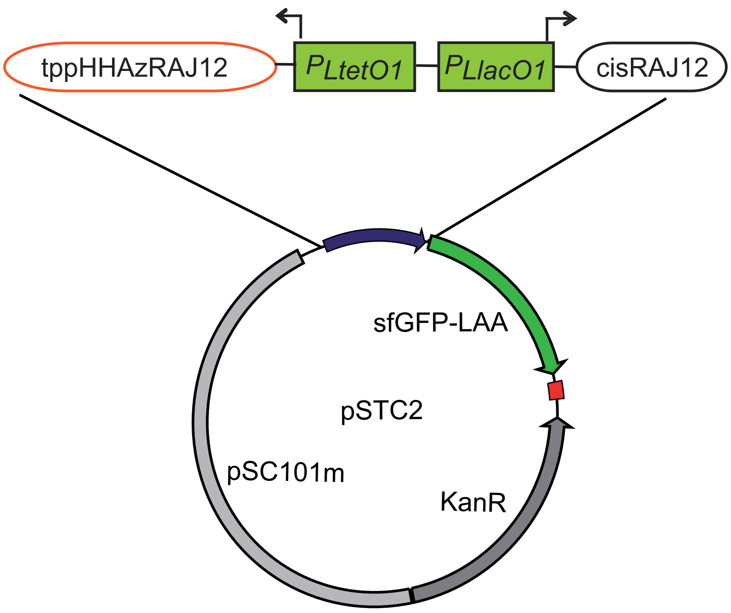

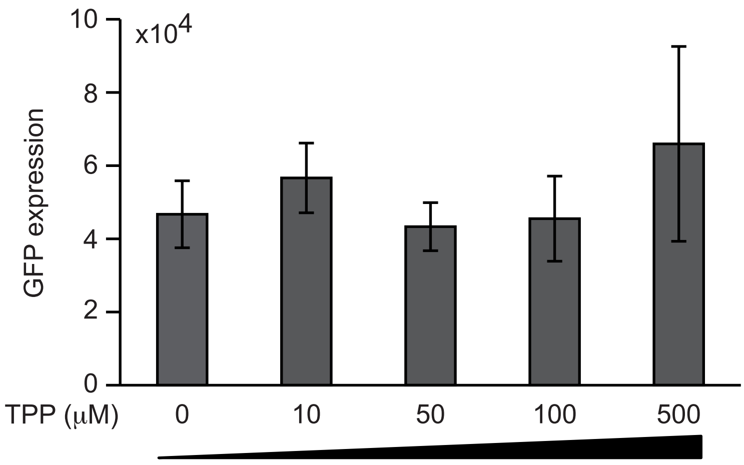

Supplementary Figure 15

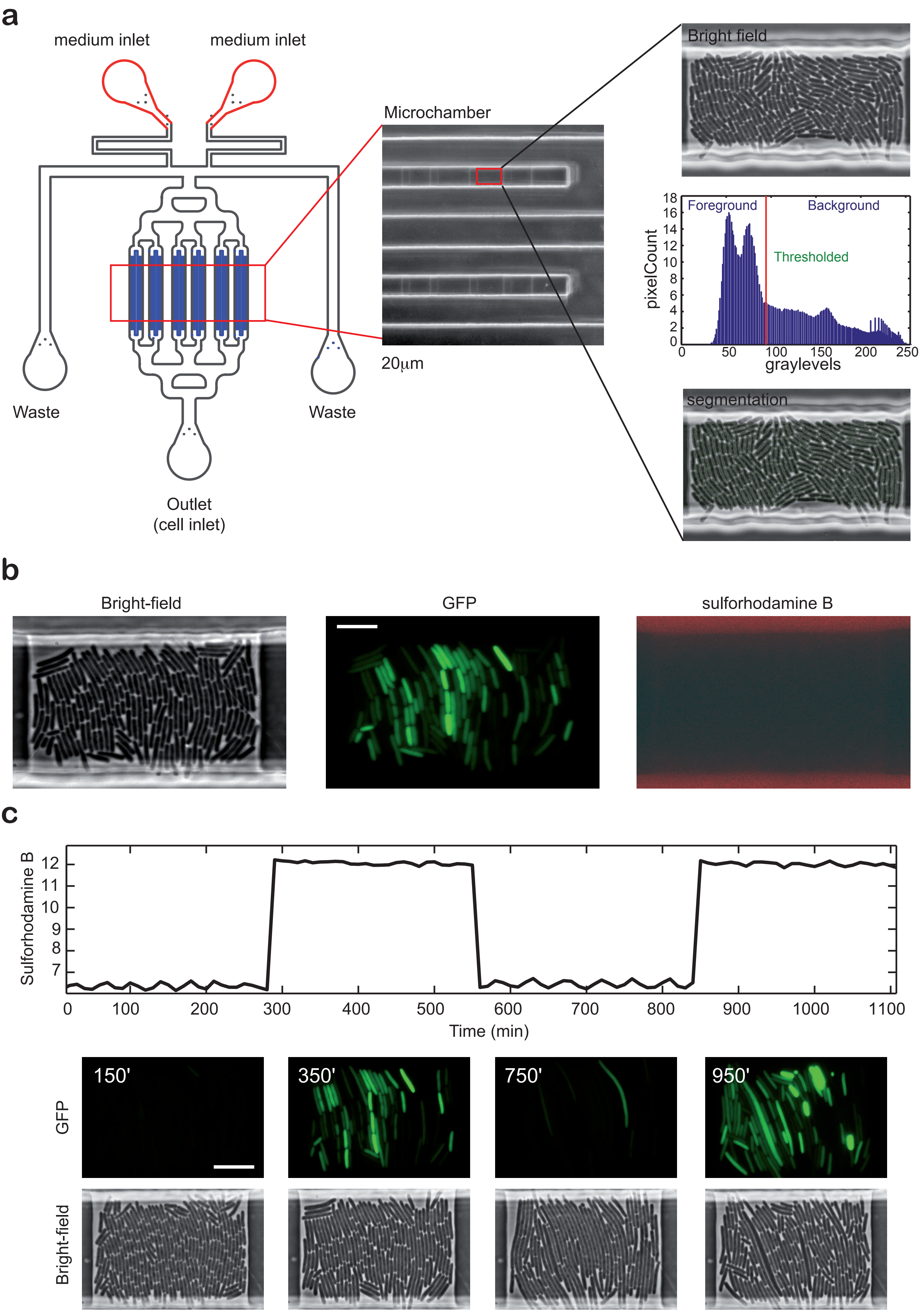

Supplementary Figure 16

a

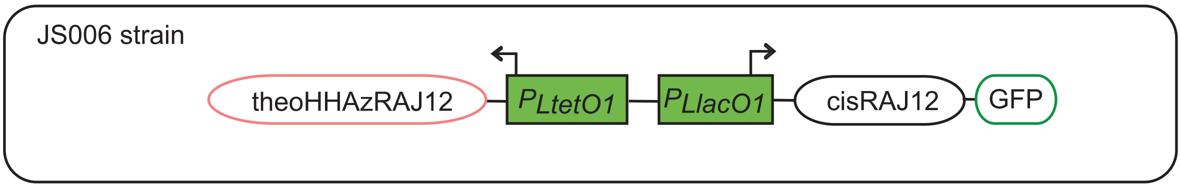

Theophylline (25 mM) forcing T = 8 h

b

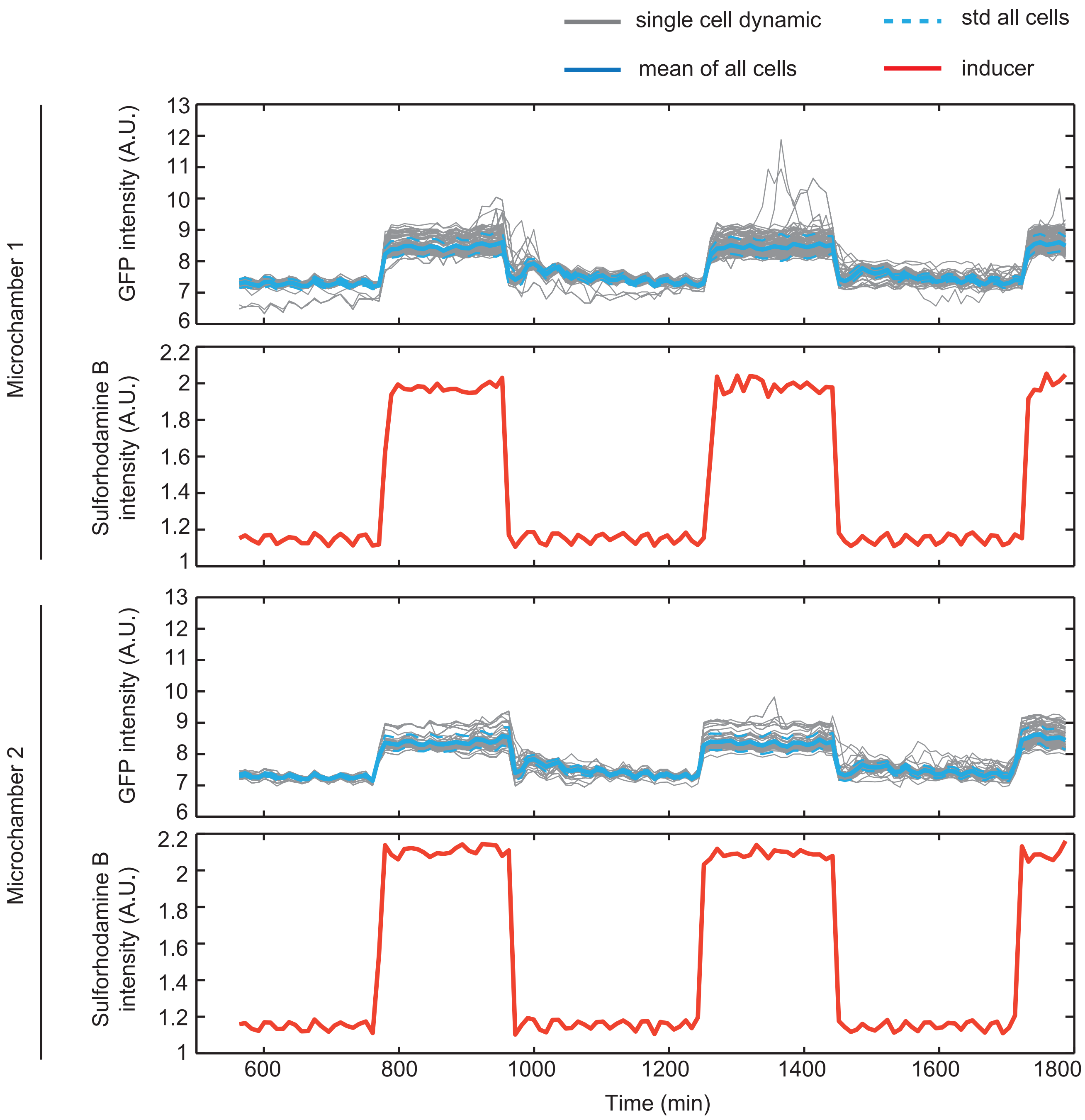

Supplementary Figure 17

a

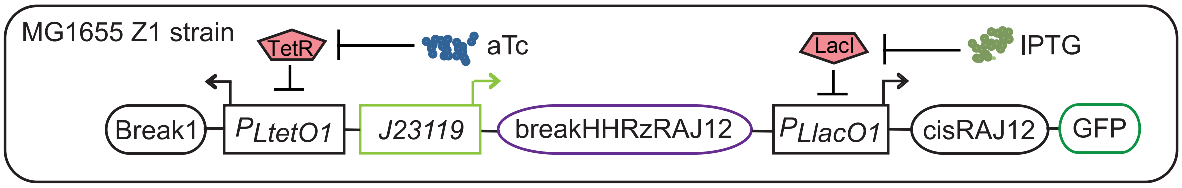

IPTG (1mM) constant

aTc (100ng/mL) forcing T = 8 h

b

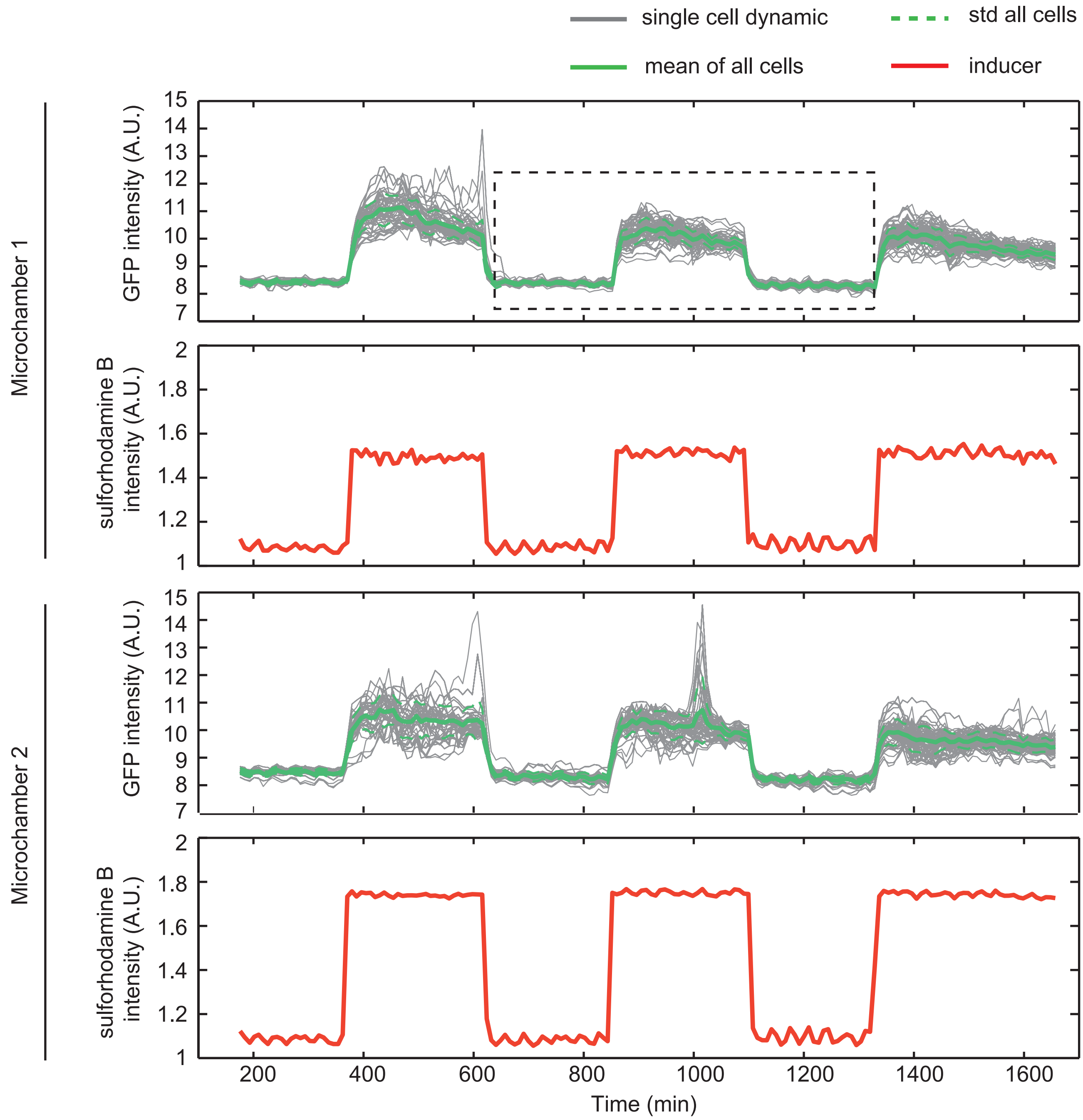

Supplementary Figure 18

a

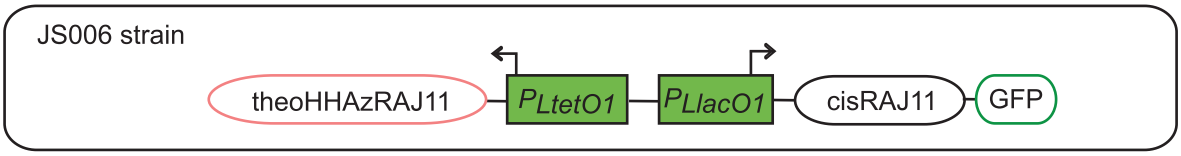

b

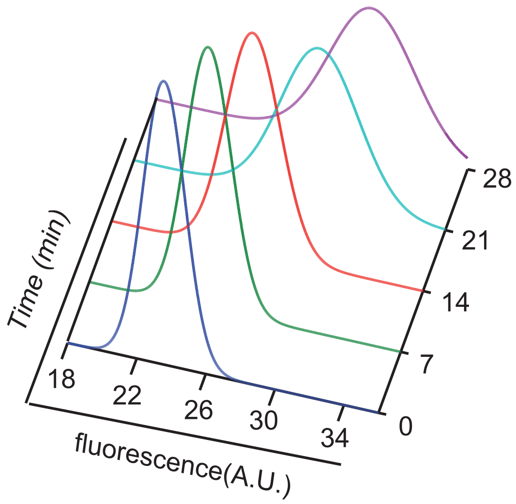

c

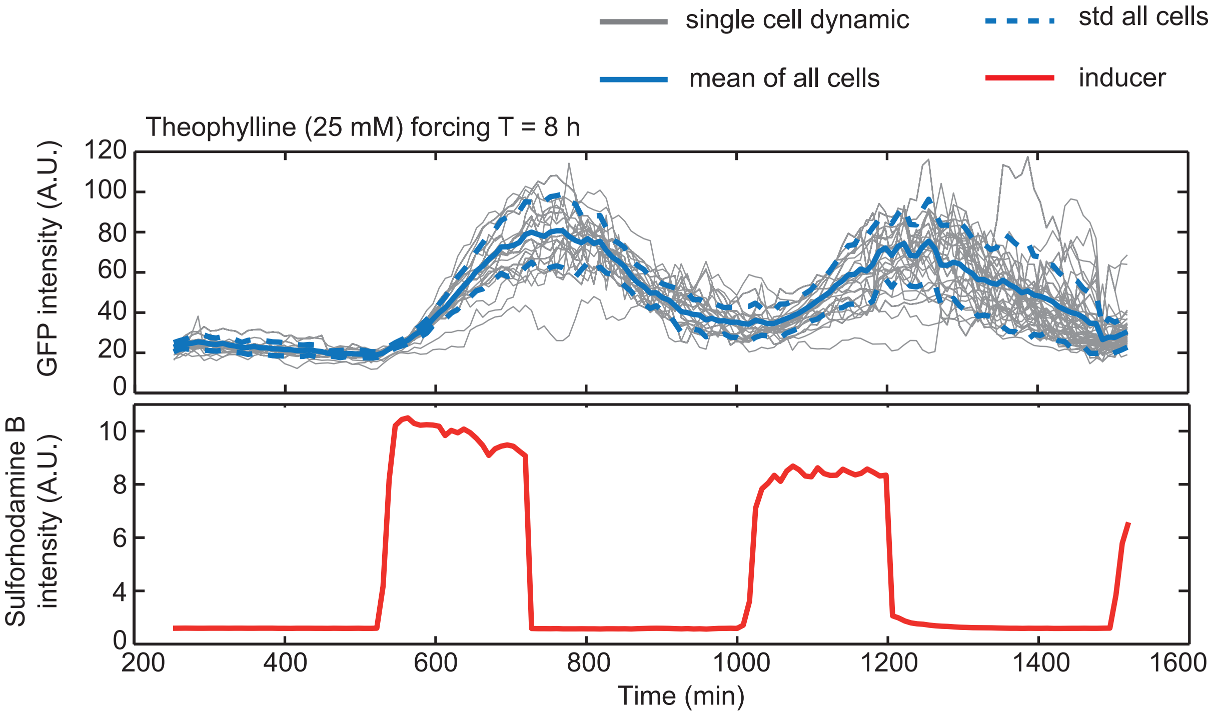

Supplementary Figure 19

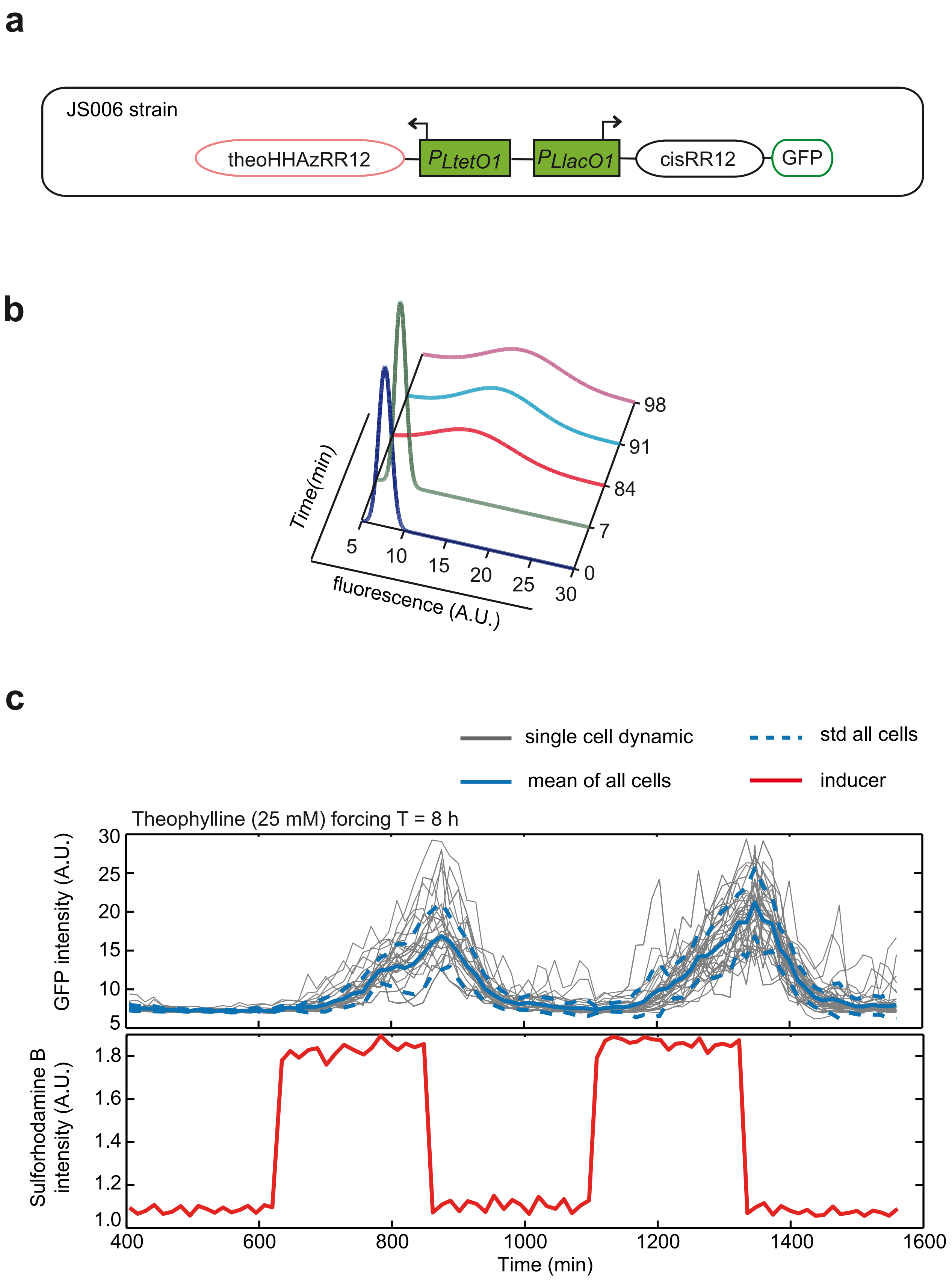

Supplementary Figure 20

a

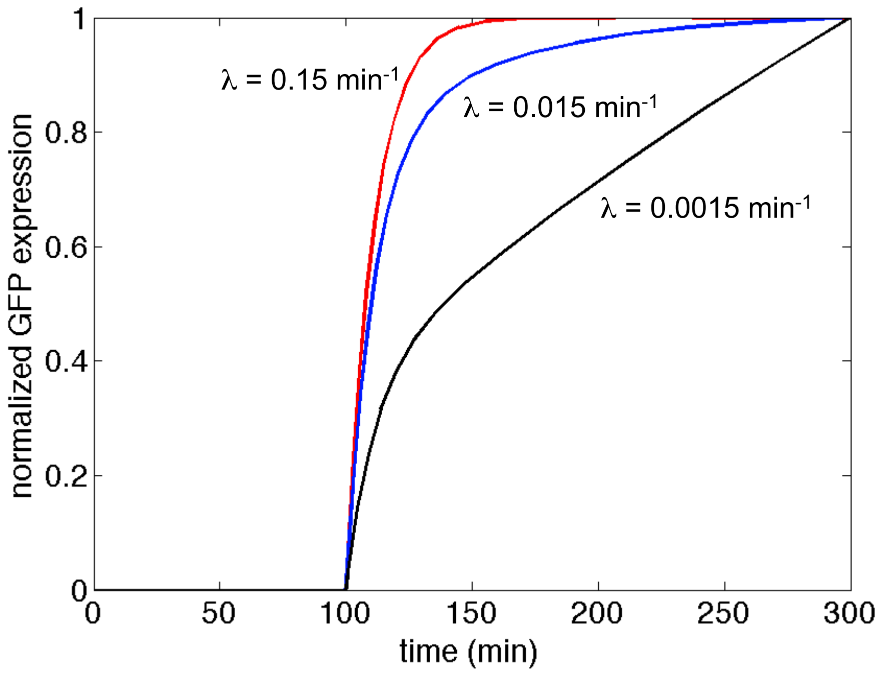

b

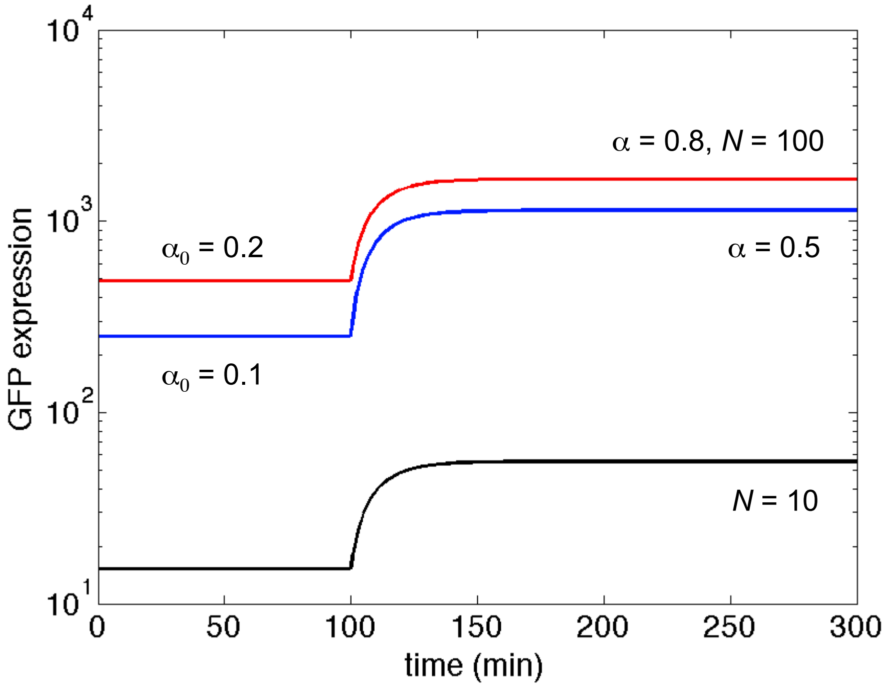

Supplementary Figure 21

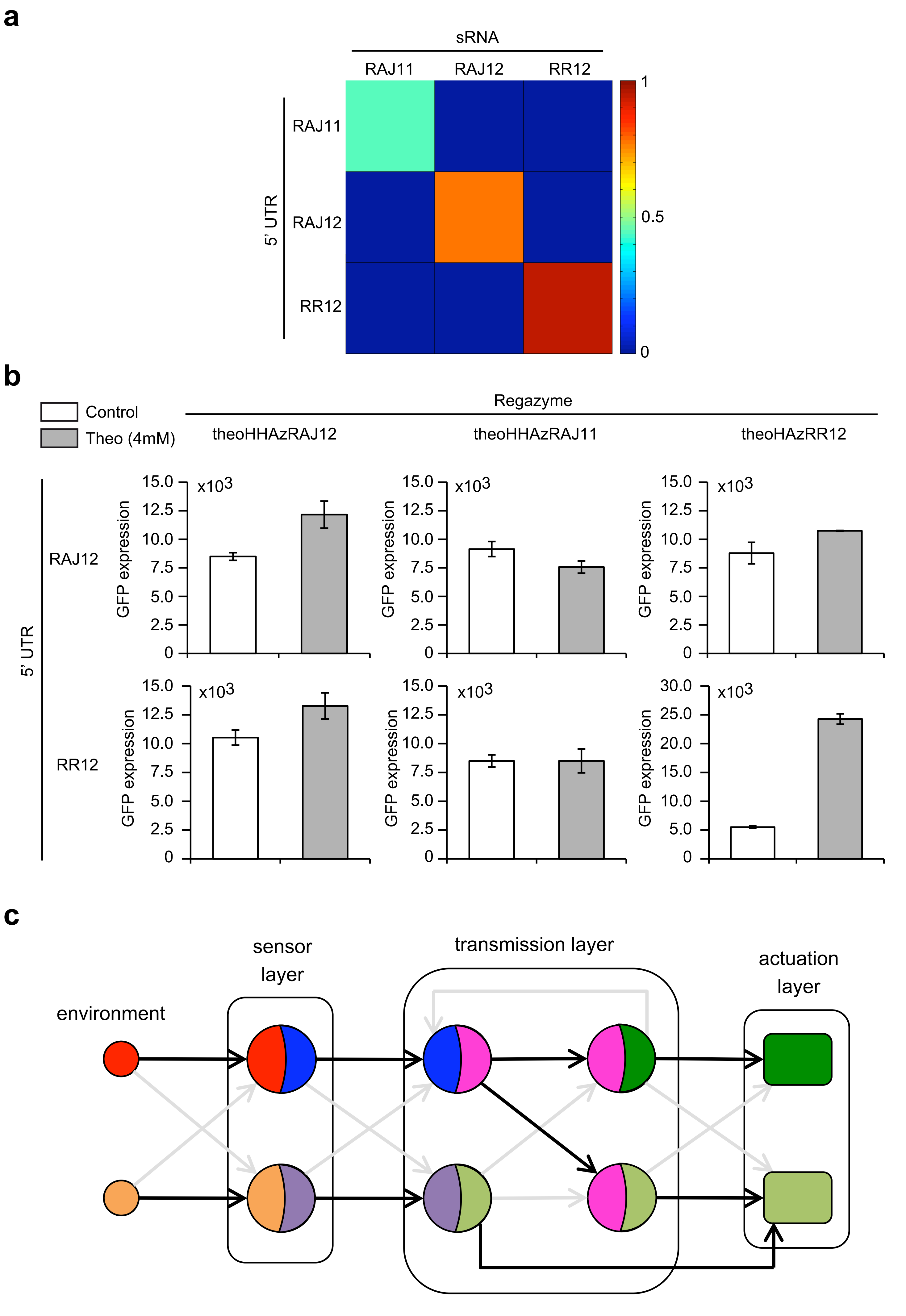

Supplementary Figure 22

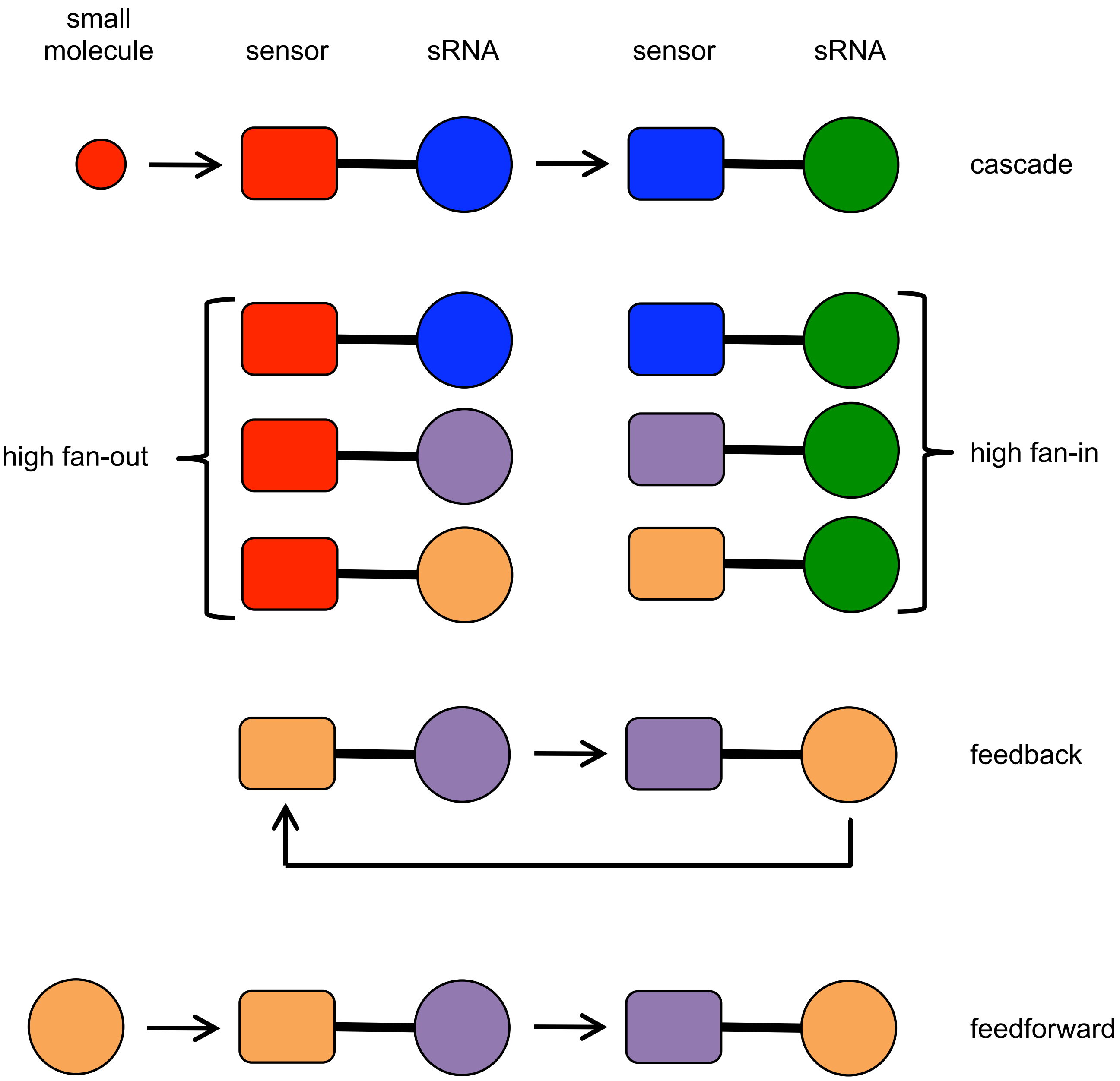

Supplementary Figure 23
